# RUNX1-induced upregulation of PTGS2 enhances cell growth, migration and invasion in colorectal cancer cells

**DOI:** 10.1038/s41598-024-60296-z

**Published:** 2024-05-22

**Authors:** Weiwei Zheng, Yingchang Guo, Aihemaiti Kahar, Junwei Bai, Qinhui Zhu, Xinli Huang, Yuan Li, Bingyi Xu, Xueshan Jia, Gang Wu, Chao Zhang, Yuanzeng Zhu

**Affiliations:** 1 Department of Gastrointestinal Surgery, Henan Provincial People’s Hospital, People’s Hospital of Zhengzhou University, No. 7 Weiwu Road, Zhengzhou, 450003 Henan China; 2 Hepatobiliary Gastrointestinal Surgery Department, Red Star Hospital of the 13th Division of Xinjiang Production and Construction Corps, Hami, 839000 The Xinjiang Uygur Autonomous Region, China China; 3 The Affiliated People’s Hospital of Xinxiang Medical College, Xinxiang, 453000 Henan China; 4 Department of Interventional Therapy, The First Affiliated Hospital of Xinxiang Medical College, Xinxiang, 453000 Henan China; 5 Department of General Surgery, Shangcai People’s Hospital, Zhumadian, 463800 Henan China; 6 Department of General Surgery, Suiping People’s Hospital, Zhumadian, 463100 Henan China; 7 Department of Anesthesiology, Henan Provincial People’s Hospital, People’s Hospital of Zhengzhou University, Zhengzhou, 450003 Henan China; 8 Weihui People’s Hospital, Weihui, 453100 Henan China; 9 Development Department, Henan Provincial People’s Hospital, People’s Hospital of Zhengzhou University, Zhengzhou, 450003 Henan China

**Keywords:** Colorectal cancer, PTGS2, Transcription factor (TF), RUNX1, Cancer, Cell biology, Molecular biology, Diseases, Gastroenterology, Oncology

## Abstract

Colorectal cancer (CRC) arises via the progressive accumulation of dysregulation in key genes including oncogenes and tumor-suppressor genes. Prostaglandin-endoperoxide synthase 2 (PTGS2, also called COX2) acts as an oncogenic driver in CRC. Here, we explored the upstream transcription factors (TFs) responsible for elevating PTGS2 expression in CRC cells. The results showed that PTGS2 silencing repressed cell growth, migration and invasion in HCT116 and SW480 CRC cells. The two fragments (499–981 bp) and (1053–1434 bp) were confirmed as the core TF binding profiles of the *PTGS2* promoter. PTGS2 expression positively correlated with RUNX1 level in colon adenocarcinoma (COAD) samples using the TCGA-COAD dataset. Furthermore, RUNX1 acted as a positive regulator of PTGS2 expression by promoting transcriptional activation of the *PTGS2* promoter via the 1086–1096 bp binding motif. In conclusion, our study demonstrates that PTGS2 upregulation induced by the TF RUNX1 promotes CRC cell growth, migration and invasion, providing an increased rationale for the use of PTGS2 inhibitors in CRC prevention and treatment.

## Introduction

Colorectal cancer (CRC) is the third most prevalent malignancy and ranks second in terms of mortality around the world^1^. There are many risk factors for CRC, including hereditary factors, environmental factors and inflammatory stimuli^2^. This cancer is a heterogeneous disease that has close relevance to a number of somatic and genetic alterations^3,4^. Recent studies have focused on investigating the molecular products, such as proteins and RNAs, that drive colorectal tumorigenesis and CRC cell malignant phenotype^3,5,6^. These molecular researches would contribute to the development of personalized medicine in the future.

Prostaglandin-endoperoxide synthase 2 (PTGS2, also called COX2), a rate-limiting cyclooxygenase that can be induced by inflammatory stimuli, is crucial for the production of inflammatory prostaglandin and possesses essential activity from normal development to human disease^7–9^. As an example, Zhou et al*.* established the role of *PTGS2* as the hub gene in promoting atherosclerosis progression^10^. Moreover, PTGS2 has been reported to strongly participate in inflammatory responses and thus contributes to liver fibrosis and chronic periodontitis^11,12^. Importantly, recent work has revealed the significant involvement of PTGS2 in cancer initiation, progression and metastasis^13,14^. Indeed, elevated PTGS2 has been implicated in enhanced angiogenesis, increased tumor invasion and reduced cell apoptosis, and inhibitors of PTGS2 have been proposed as promising therapeutic agents against cancer^15^. In CRC, PTGS2 expression is markedly promoted in tumor samples versus normal colorectal tissues and tightly associated with poorer CRC-specific survival^16,17^. Furthermore, PTGS2 has an established role in promoting colorectal tumorigenesis and CRC progression^18–20^. These studies suggest that inhibition of PTGS2 is a potential therapeutic approach in CRC; however, the upstream regulators of PTGS2 abnormal expression have not yet been explored.

Transcription factors (TFs) have the capacity of controlling the rate of gene transcription by binding to specific DNA sequences in the promoters and thus have been identified as vital regulators in cancer biology^21,22^. Runt-related TF 1 (RUNX1), a member of the core-binding factor family of TFs, is involved in the regulation of a series of cellular processes, including cell growth, differentiation, survival, and death^23,24^. There is ample evidence that RUNX1 functions as a potent driver in various human cancers^23,25,26^. Importantly, RUNX1 can promote CRC cell migration and epithelial-mesenchymal transition (EMT) through TGF-β and Wnt/β-catenin pathways^27,28^. RUNX1 contributes to CRC cell growth and chemoresistance development via the Hedgehog signaling pathway^29^. In addition, RUNX1-activated upregulation of long non-coding RNA RNCR3 drives CRC cell malignant behaviors^30^. Nonetheless, the molecular basis of the oncogenic effect of RUNX1 on CRC development is still not fully understood.

In the current study, our data support the oncogenic role of PTGS2 in CRC in vitro. Further, we focused on elucidating the TFs that regulate PTGS2 expression in CRC cells. We identify, for the first time, RUNX1 as a transcription activator of PTGS2 in CRC cells by binding to the *PTGS2* promoter and promoting its transcriptional activation.

## Materials and methods

### Cell culture and transfection

HCT116 (Cat#CL-0096, isolated from rectum tissues of patients with CRC) and SW480 (Cat#CL-0223B, isolated from colon tissues of patients with colorectal adenocarcinoma) CRC cells (Procell, Wuhan, China) were used in this study. HCT116 and SW480 cells were maintained in DMEM (Cat#G4510-500ML, Servicebio, Wuhan, China) enriched with 1% antibiotics (Cat#C0222, penicillin/streptomycin, Beyotime, Shanghai, China) and 10% fetal bovine serum (FBS) (Cat#G8001-100ML, Servicebio). We maintained the two cell lines at 37 °C in a cell culture incubator containing 5% CO_2_.

Short hairpin RNAs (shRNAs) to human *RUNX1* (RUNX1-shRNA1, RUNX1-shRNA2, RUNX1-shRNA3), *PTGS2* (sh-PTGS2#1 and sh-PTGS2#2) and a scrambled control sh-NC were obtained from Tsingke Biotechnology (Beijing, China). Sequences of shRNAs are displayed in Table 1. Human RUNX1 expression plasmid pLV3-RUNX1 and PTGS2 expression plasmid LV-PTGS2 were procured from Miaolingbio (Wuhan, China), pLV3-NC was used as a control. For plasmid transfection, HCT116 and SW480 cells were plated in 6-well culture dishes until the cell confluence reached 80–90%. The mixture of 3 μg plasmids and 5 μL RFect Plasmid DNA Transfection Reagent (Cat#21015, Baidai, Changzhou, China) was prepared and added into each well for 12 h. Then cells were harvested for following cell functional assays.

**Table 1:** Sequences for shRNAs.

Name	Sequences of shRNA (5’-3’)
shPTGS2#1	GATTGAAGATTATGTGCAA
shPTGS2#2	GAAATGCAATTATGAGTTA
RUNX1-shRNA1	CTACGATCAGTCCTACCAATA
RUNX1-shRNA2	TCGCCCTGTTTGGCATCTAAT
RUNX1-shRNA3	GAACCAGGTTGCAAGATTTAA

### Cell viability analysis

Transfected HCT116 and SW480 cells were seeded at 1 × 10^4^ cells/well in 96-well cell culture dishes. After a 48-h culture, metabolically active cells were examined by adding 10 µL of Cell Counting Kit-8 (CCK-8, Cat#C0038) solution to each well, as per manufacturing protocol (Beyotime). The plates were analyzed in a microplate reader after 1 h incubation. Cell viability was assessed as fold change of control group.

### Calcein/PI Cell Viability/Cytotoxicity Assay

Transfected HCT116 and SW480 cells were seeded at 2 × 10^4^ cells/well in 96-well cell culture dishes. After a 48-h culture, metabolically active cells were examined byusing Calcein/PI Cell Viability/Cytotoxicity Assay Kit (Cat#C2015M), as per manufacturing protocols (Beyotime). The cells were observed under a fluorescence microscope (Olympus, Tokyo, Japan), and images of three random fields per each sample were obtained. The number of stained cells was quantified by ImageJ (NIH, Bethesda, MA, USA) and cell viability was calculated by using the method: cell viability (%) = (number of Calcein AM^+^ cells)/(number of Calcein AM^+^ cells + number of PI^+^ cells) × 100.

### Cell proliferation analysis

HCT116 and SW480 cells transfected as indicated were seeded in 96-well dishes at 1 × 10^4^ cells/well and incubated in standard growth media for 48 h. Cell growth was evaluated by detecting proliferation with Click-iT EdU-555 or EdU-488 Cell Proliferation Kit (Cat#G1602 or Cat#G1601) as recommended by the manufacturer (Servicebio). Briefly, EdU solution was added into each well at a final concentration of 10 µM and incubation was allowed for 2–3 h. The cells were then fixed with 4% paraformaldehyde and stained with iF555 solution (for EdU incorporation) for 30 min at room temperature, followed by Hoechst 33,342 solution (for nucleus staining) for 5 min. Images of three random fields per each sample were captured in an Olympus fluorescence microscope, and the EdU positive cells were defined as a percentage of total nuclei.

### Immunofluorescence

The effect of sh-PTGS2, RUNX1-shRNA3, or sh-NC control on CRC cells was evaluated by detecting matrix metallopeptidase 9 (MMP9) and PCNA expression with an immunocytochemical method. 24 hh post transfection, HCT116 and SW480 cells were fixed, permeabilized by incubation in 0.5% Triton X-100, and washed in 1 ×  phosphate buffered saline (PBS). Following blocking in 3% BSA (Cat#ST2249-5g, Beyotime) for 30 min, probing was carried out with primary antibody (Servicebio) against PCNA (Cat#GB11010, 1:500 dilution) or MMP9 (Cat#GB11132, 1:1000 dilution) as described^31^. The Alexa Fluor 488 goat anti-rabbit IgG (Cat#GB25303, Servicebio) at 1:500 dilution in 1 ×  PBS was used as the secondary antibody. After nuclear staining with DAPI, the cells were visualized on the Olympus fluorescence microscope. Images of three random fields were obtained and the fluorescence intensity was quantified by ImageJ to get an average fluorescence intensity of PCNA or MMP9.

### Transwell migration and invasion assays

After being re-suspended in the serum-free medium, transfected HCT116 and SW480 cells were seeded on 24-transwell inserts (8 µm pore size, Corning, Shanghai, China) with (for invasion analysis) or without (for migration analysis) growth factor reduced Corning® Matrigel as previously reported^32^. The inserts were placed in appropriate wells containing complete cell culture media and incubated for 48 h, followed by staining with crystal violet (0.1%, Cat#GC307002-25g, Servicebio). Migratory or invaded cells on the lower surface were visualized using bright-field microscopy (Olympus) at 100× magnification. Pictures of at least three random fields from three replicate wells were obtained and the number of the migratory and invaded cells was quantified by ImageJ.

### JC-1 cell apoptosis assay

Apoptosis of transfected HCT116 and SW480 cells was evaluated by measuring cell mitochondrial membrane potential using JC-1 Assay Kit (Cat#C2006, Beyotime). Briefly, after being resuspended in DMEM growth medium, cells were incubated with JC-1 working solution for 20 min at 37 °C. The signals of red fluorescence (excitation/emission wavelengths: 525/590 nm) and green fluorescence (excitation/emission wavelengths: 490/530 nm) were observed under the Olympus fluorescence microscope. Images of three random fields were analyzed by ImageJ software, and the ratio of red fluorescence/green fluorescence was determined.

### Bioinformatics

The sequence of the human *PTGS2* promoter was retrieved from Ensemble database (http://uswest.ensembl.org/index.html). The data of all human TFs were downloaded from JASPAR database (https://jaspar.genereg.net/). The TF binding profiles of the *PTGS2* promoter were predicted by JASPAR database. The known TFs in regulating PTGS2 expression were retrieved from KnockTF database (http://www.licpathway.net/KnockTF/). To predict the binding sites between RUNX1 and the *PTGS2* promoter, we also utilized JASPAR database. To observe the expression correlation of PTGS2 and RUNX1 and the clinical significance of RUNX1 in CRC, we interrogated the TCGA-COAD dataset using the National Cancer Institute GDC Data Portal (https://portal.gdc.cancer.gov/).

### DNA pull-down assay and qualitative proteome analysis

The full-length sequence of the human *PTGS2* promoter was synthesized by Tsingke Biotechnology. The biotinylated promoter full-length sequence (P0: 0–2000 bp) and three promoter truncations labeled by biotin (P1: 0–981 bp, P2: 499–1434 bp and P3: 1052–2000 bp) were PCR-amplified using corresponding biotinylated primers (synthesized by Tsingke Biotechnology; shown in Table 2) with the synthetic *PTGS2* promoter sequence as template. The PCR reaction system (50 µL) consisted of 5 µL template, 2 µL Primer F, 2 µL Primer R, 16 µL ddH_2_O, and 25 µL Phanta Flash Mix (2 × , Cat#P520-01, Vazyme Biotech, Nanjing, China). PCR amplification was performed with the following conditions: 98 °C for 2 min, followed by 35 cycles at 98 °C for 10 s, and 50 °C for 15 s and 72 °C for 15 s. PCR productions were validated by 1.5% agarose gel eletrophoresis. The biotin labeling effect was examined using a biotin labeling efficiency assay with an HRP-conjugated streptavidin (Cat#G3431, 1:3000, Servicebio) and ECL Kit (Cat#P0018S, Beyotime). Briefly, PCR production was adjusted to a concentration of 20 ng/µL and added on the nylon membranes (0.45 µm pore size, Beyotime). After ultraviolet crosslinking for 10 min and incubation with blocking solution (Beyotime) for 20 min, the membranes were probed with the HRP-conjugated streptavidin for 1 h at room temperature and visualized by the ECL Kit.

**Table 2:** Sequences of primers used for PCR amplification.

Name		Primers for PCR (5′–3′)
P0 (1–2000 bp)	Forward	AGAAAGCTCTGGAAGATTTTTAAG
	Reverse	GAAGTCACGTCGGGACAGAC
P1 (1–981 bp)	Forward	AGAAAGCTCTGGAAGATTTTTAAG
	Reverse	TCTGATTCTTCATGAGACACGG
P2 (499–1434 bp)	Forward	GGGATAGATGGAGTTCAATTC
	Reverse	TGACCATGGATCAAAGTACAAC
P3 (1053–2000 bp)	Forward	ACTTCTACAAATTGAGGTACCTGG
	Reverse	GAAGTCACGTCGGGACAGAC
RUNX1	Forward	CACTGTGATGGCTGGCAATG
	Reverse	CCTCTTCCACTTCGACCGAC
PTGS2	Forward	GGAGGTCTTTGGTCTGGTGC
	Reverse	ACAACTGCTCATCACCCCAT
GAPDH	Forward	GACCACAGTCCATGCCATCAC
	Reverse	ACGCCTGCTTCACCACCTT

For DNA pull-down assay, the nuclear protein of HCT116 CRC cells was extracted using Nuclear and Cytoplasmic Protein Extraction Kit (Cat#0027, Beyotime). Streptomycin beads were obtained from Beyotime and washed three times with 1 ×  TBST. A total of 10 µg each of the four biotin-labeled DNA sequences (P0, P1, P2 and P3) dissolved in binding&washing buffer was added separately into 35 µL streptomycin beads and incubation was performed for 30 min at room temperature in a vertical rotating mixer. After that, the nuclear protein extracts were incubated with the generated DNA-bead complex for 1 h at room temperature. Beads were washed three times with PBST, and bound proteins were eluted with the protein eluent, followed by boiling for 5 min in SDS loading buffer. The precipitated proteins in each group were subsequently processed for qualitative proteome analysis by Qinglianbio Biotechnology Co., Ltd. (Beijing, China) with RIGOL L-3000 HPLC System (RIGOL, Beijing, China) and Proteome Discoverer2.4 software.

### Quantitative real-time PCR (qRT-PCR)

Transfected HCT116 cells were homogenized in RNAiso Plus (Cat#9109, TaKaRa, Dalian, China) after treatment of recombinant DNase I (Cat#2270A, TaKaRa). cDNA was randomly primed using PrimeScript™ RT Master Mix (Cat#RR036A, TaKaRa) from 2 µg of total RNA with random hexamers. Then, cDNA was diluted 15–20 fold and subjected to qRT-PCR using SYBR Premix ExTaq Kit (Cat#RR420A, TaKaRa) with primers specific for *RUNX1* and *PTGS2* (synthesized by Tsingke Biotechnology; shown in Table 2). Using *GAPDH* as a reference gene, we calculated mRNA expression with the 2^-ΔΔCt^ method^33^.

### Dual-luciferase reporter assay

The F4 fragment (1053–1434 bp) of the *PTGS2* promoter and its three mutants in the individual binding site (site 1, 1062–1072 bp: AATTGAGGTAC; site 2, 1086–1096 bp: ATTTCAGGTTT; site 3, 1373–1383 bp: TATTGGGGCTA) were synthesized by Tsingke Biotechnology and cloned into the pGL3-basic vector (Promega, Southampton, UK), respectively. For luciferase reporter assay to measure the effect of RUNX1 on the transcription activity of the *PTGS2* promoter, HCT116 cells (1 × 10^5^ cells/well) were seeded in the wells of 6-well dishes one day before transfection with a mixture of 200 ng pGL3 luciferase constructs, 50 ng of pRL-TK *Renilla* internal control vector (Promega), and pLV3-RUNX1 or pLV3-NC. Cells were lysed 24 h later, and luciferase activity was assayed with the Dual-luciferase Reporter System (Cat#E1910, Promega). Firefly luciferase activity was normalized to *Renilla* activity and expressed as relative luciferase activity.

### Statistical analysis

The results were representative of at least three separate experiments performed in triplicate. All error bars represent the SD of the mean. Differences were evaluated using GraphPad Prism 8 (GraphPad Inc., La Jolla, CA, USA) by one-way or two-way ANOVA with Tukey’s or Sidak’s multivariate comparison (for three or more groups) or a unpaired Student’s *t*-test (two-tailed, for two groups) and considered significant when *P* < 0.05. Pictures were plotted by GraphPad Prism 8 and R-ggplot2 package. For survival analysis, the R-survival and R-survminer were used.

## Results

### Silencing of PTGS2 represses growth, migration and invasion of HCT116 and SW480 CRC cells

**Figure 1:**
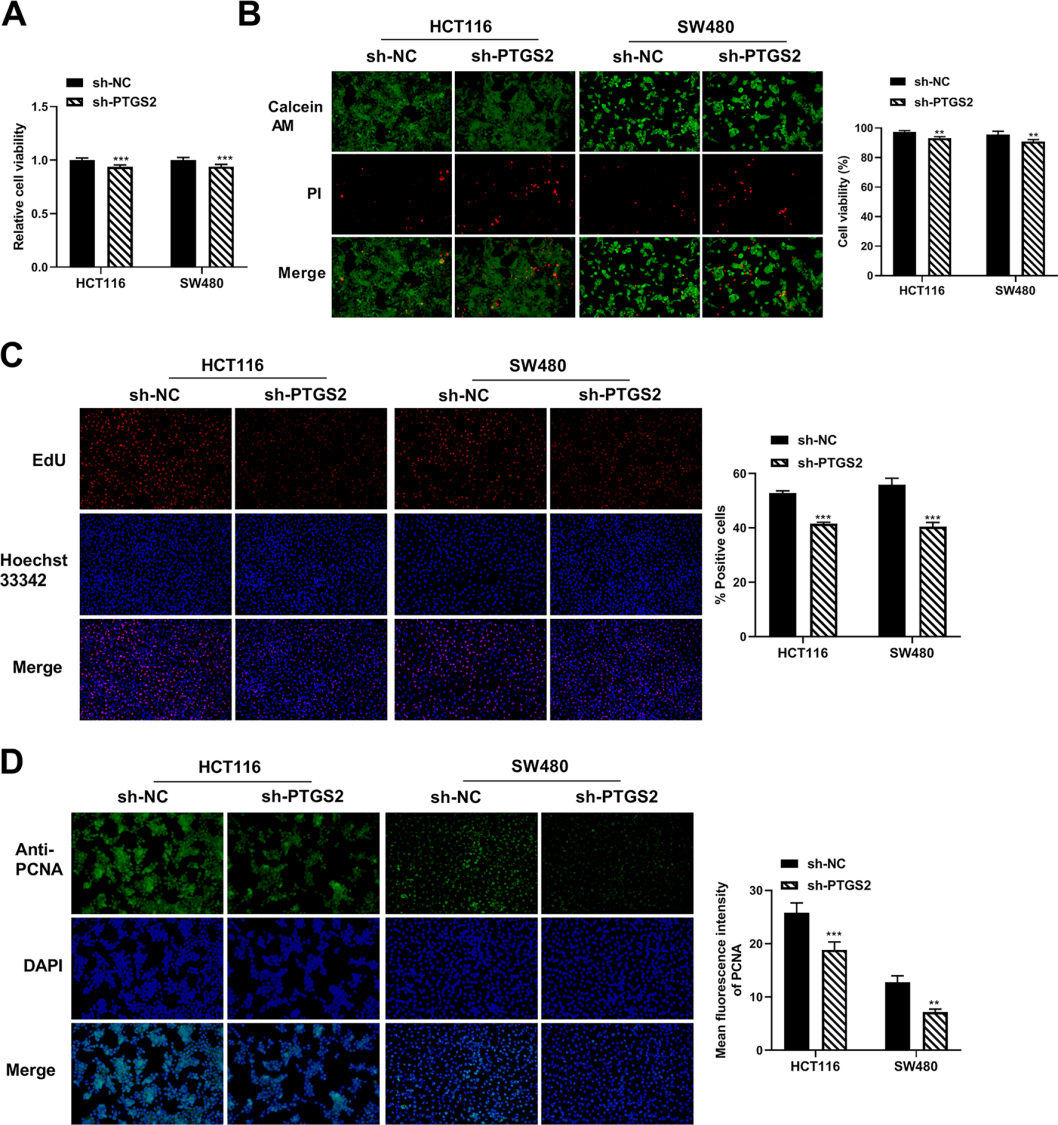
PTGS2 silencing impedes the growth of HCT116 and SW480 CRC cells in vitro. (**A**, **B**) Cell viability assay was performed with HCT116 and SW480 CRC cells after transfection by sh-NC or sh-PTGS2#2 (also called sh-PTGS2) using CCK8 and Calcein/PI Cell Viability/Cytotoxicity Assay Kit. Images of three random fields per each sample were obtained. The number of stained cells was quantified by ImageJ and cell viability was calculated by using the method: cell viability (%) = (number of Calcein AM^+^ cells)/(number of Calcein AM^+^ cells + number of PI^+^ cells). (**C**) EdU assay for cell proliferation was performed with HCT116 and SW480 CRC cells transfected with sh-PTGS2 or sh-NC. Cells were incubated with EdU solution and stained with iF555 solution and Hoechst 33,342 solution. Images of three random fields per each sample were captured using a fluorescence microscope and the EdU positive cells (%positive cells) were defined as a percentage of total nuclei. Representative images per each group are shown. (**D**) Immunofluorescence assay showing PCNA fluorescence intensity in HCT116 and SW480 cell lines transfected as indicated. Cells were incubated with anti-PCNA antibody and secondary antibody. Cell nucleus was stained with DAPI. Images of three random fields were obtained and the fluorescence intensity was quantified by ImageJ to get an average fluorescence intensity of PCNA. Representative images per each sample are shown. n = three independent experiments performed in triplicate. **P < 0.01; ***P < 0.001.

To test the effects of PTGS2 on cell growth, motility and invasion, we transfected HCT116 and SW480 CRC cells with two shRNAs specific for PTGS2 (sh-PTGS2#1 and sh-PTGS2#2) or a nontarget shRNA (sh-NC). The reduction efficacy of shRNAs in PTGS2 mRNA expression was validated by qRT-PCR (Supplementary Fig. 1A). Owing to the more significant inhibition of sh-PTGS2#2 on PTGS2 mRNA expression (Supplementary Fig. 1A), we used sh-PTGS2#2 (also named sh-PTGS2) for the subsequent silencing experiments. Silencing of PTGS2 by sh-PTGS2 significantly repressed CRC cell viability compared with the sh-NC control (Fig. 1A,B). EdU assays revealed that silencing of PTGS2 reduced the number of EdU positive cells in HCT116 and SW480 CRC cells after 48 h (Fig. 1C). Moreover, HCT116 and SW480 CRC cells transfected with sh-PTGS2 showed decreased expression of the proliferating marker PCNA compared with the control cells (Fig. 1D). Altogether, these data indicate that PTGS2 silencing represses CRC cell growth in vitro.

**Figure 2:**
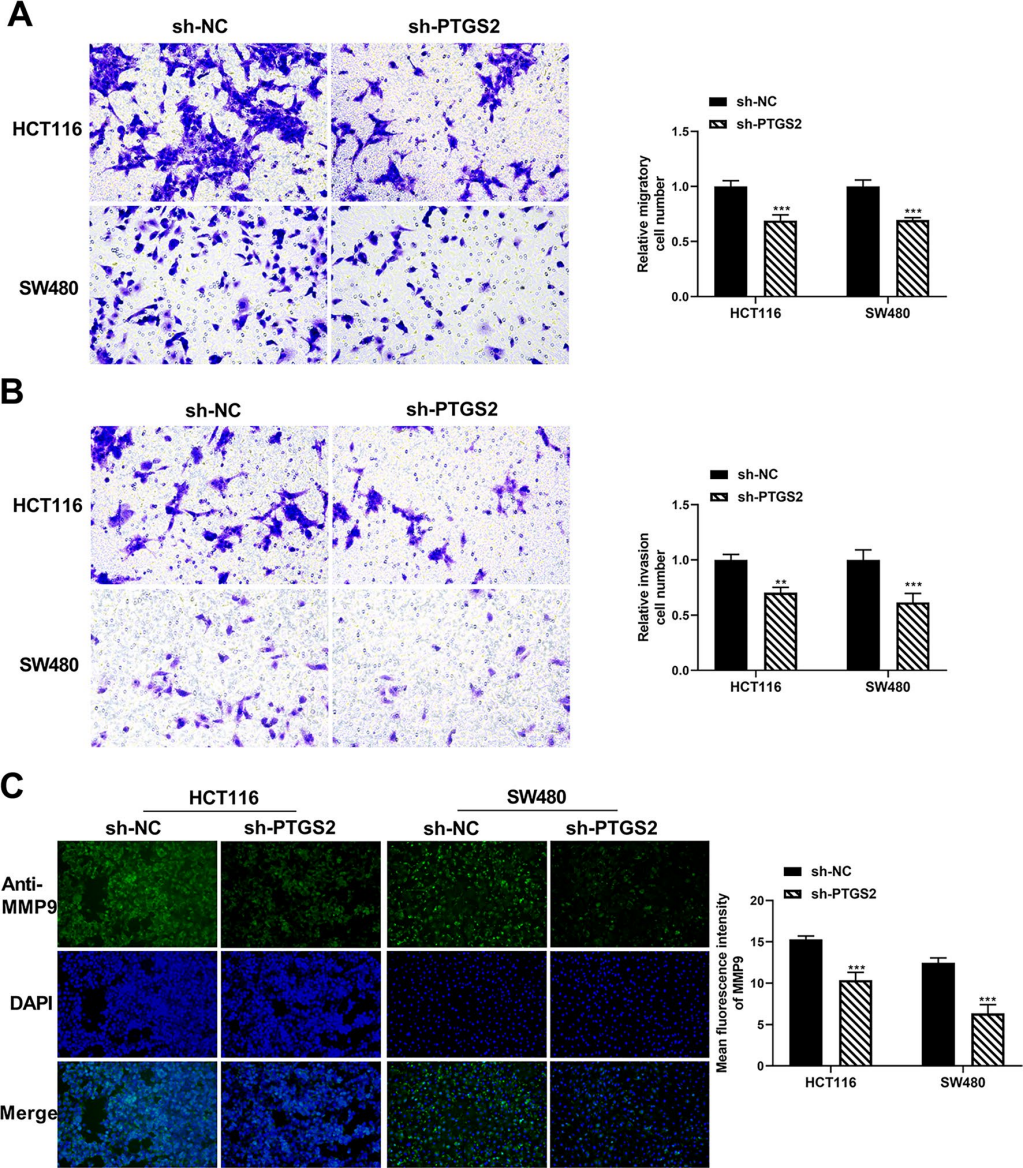
PTGS2 silencing represses the migration and invasion of HCT116 and SW480 CRC cells. (**A**, **B**) Transwell migration and invasion assays of HCT116 and SW480 CRC cells transfected with sh-PTGS2 or sh-NC. Transfected cells were seeded on 24-transwell inserts and translocated toward the complete growth medium. After 48 h of culture, pictures of at least three random fields from three replicate wells were obtained by a × 100 magnification microscope and the number of the migratory and invaded cells was quantified by ImageJ. (**C**) Immunofluorescence assay showing the fluorescence intensity of MMP9 in HCT116 and SW480 cells after transfection by sh-PTGS2 or sh-NC. Cells were incubated with anti-MMP9 antibody and secondary antibody. Cell nucleus was stained with DAPI. Images of three random fields were obtained and the fluorescence intensity was quantified by ImageJ to get an average fluorescence intensity of MMP9. Representative images per each sample are shown. n = three independent experiments performed in triplicate. **P < 0.01; ***P < 0.001.

**Figure 3:**
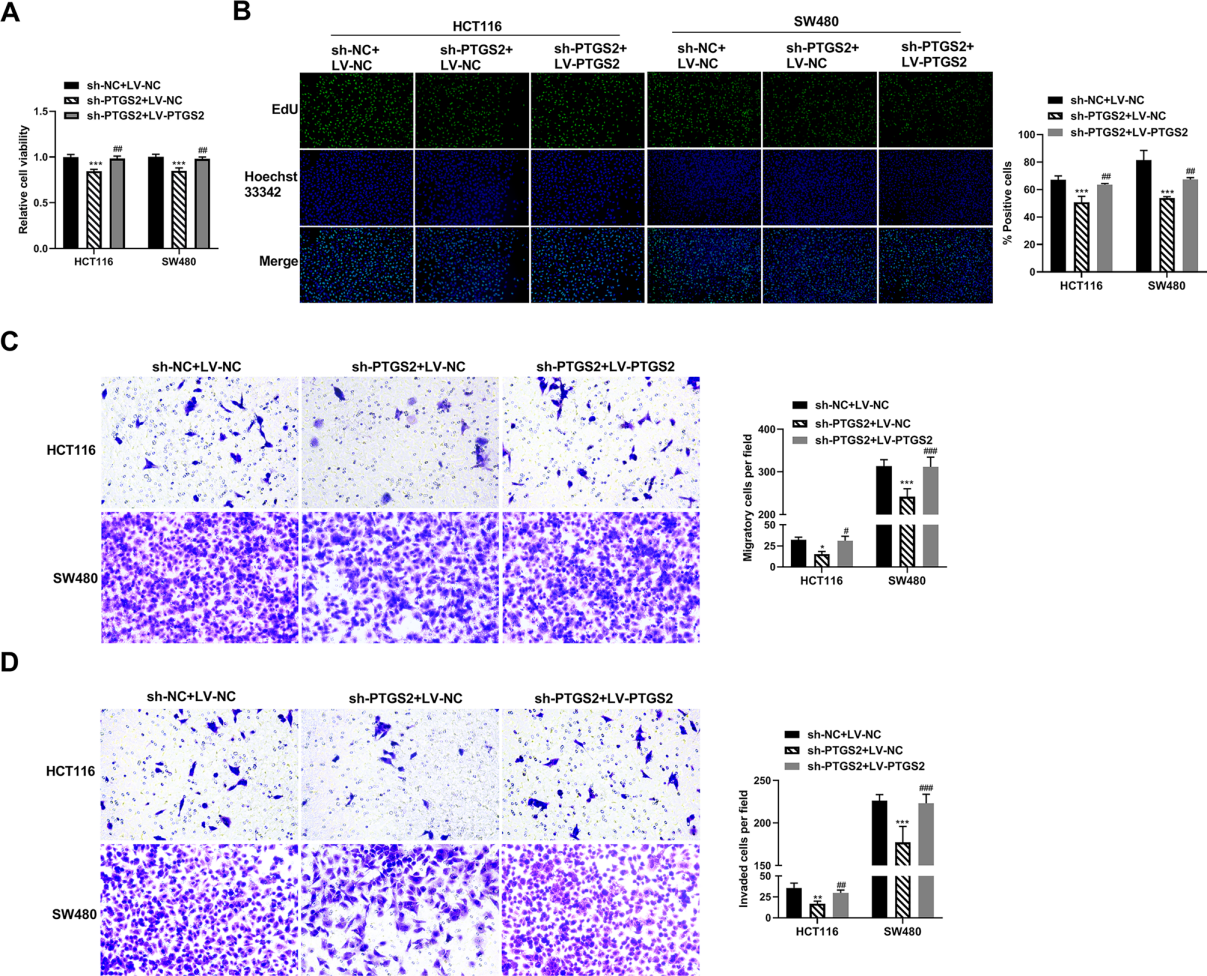
The PTGS2 expression plasmid reverses PTGS2 silencing-mediated suppression of CRC cell growth, migration and invasion. (**A**) Cell viability assay was performed with HCT116 and SW480 CRC cells after transfection by sh-PTGS2 + LV-NC, sh-PTGS2 + LV-PTGS2 or sh-NC + LV-NC using CCK8 assay. (**B**) Cell proliferation was performed by EdU assay with HCT116 and SW480 CRC cells transfected with sh-PTGS2 + LV-NC, sh-PTGS2 + LV-PTGS2 or sh-NC + LV-NC. Cells were incubated with EdU solution and stained with iF488 solution and Hoechst 33,342 solution. Images of three random fields per each sample were captured using a fluorescence microscope and the EdU positive cells (%positive cells) were defined as a percentage of total nuclei. Representative images per each group are shown. (**C**, **D**) Transwell migration and invasion assays of HCT116 and SW480 CRC cells transfected with sh-PTGS2 + LV-NC, sh-PTGS2 + LV-PTGS2 or sh-NC + LV-NC. Transfected cells were seeded on 24-transwell inserts and translocated toward the complete growth medium. After 48 h of culture, pictures of at least three random fields from three replicate wells were obtained by a 100 × magnification microscope and the number of the migratory and invaded cells was quantified by ImageJ. *P < 0.05, **P < 0.01; ***P < 0.001; ^##^*P* < 0.01; ^###^*P* < 0.001.

Using a transwell assay to study the effect on cell migration and invasion, we found that inhibition of PTGS2 by sh-PTGS2 introduction strongly impaired the migratory capacity of the two CRC cell lines (Fig. 2A). Moreover, silencing of PTGS2 impeded cell invasiveness compared with the sh-NC control (Fig. 2B). Through an immunofluorescence method, we found that PTGS2 inhibition led to a clear downregulation in the expression of the metastasis-related protein MMP9 in both HCT116 and SW480 CRC cell lines (Fig. 2C), supporting the notion that PTGS2 silencing impairs cell migration and invasion in vitro. Additionally, JC-1 fluorescence assays showed that CRC cells transfected with sh-PTGS2 had a lower ratio of red/green fluorescence signals than the controls (Supplementary Fig. 2), indicating that PTGS2 inhibition can promote CRC cell apoptosis in vitro. Lastly, to confirm that the observed phenotypes are indeed due to downregulation of PTGS2 and not to an off-target effect, we performed a rescue experiment by transfecting a PTGS2 expression plasmid into PTGS2-silenced HCT116 and SW480 CRC cells and found that the plasmid reversed sh-PTGS2-mediated inhibition of PTGS2 expression (Supplementary Fig. 1B). Furthermore, the PTGS2 expression plasmid reversed sh-PTGS2-mediated suppression of growth, migration and invasion of HCT116 and SW480 CRC cells (Fig. 3A–D).

### The two fragments (499-981 bp) and (1053-1434 bp) are the core TF binding profiles of the* PTGS2* promoter

**Figure 4:**
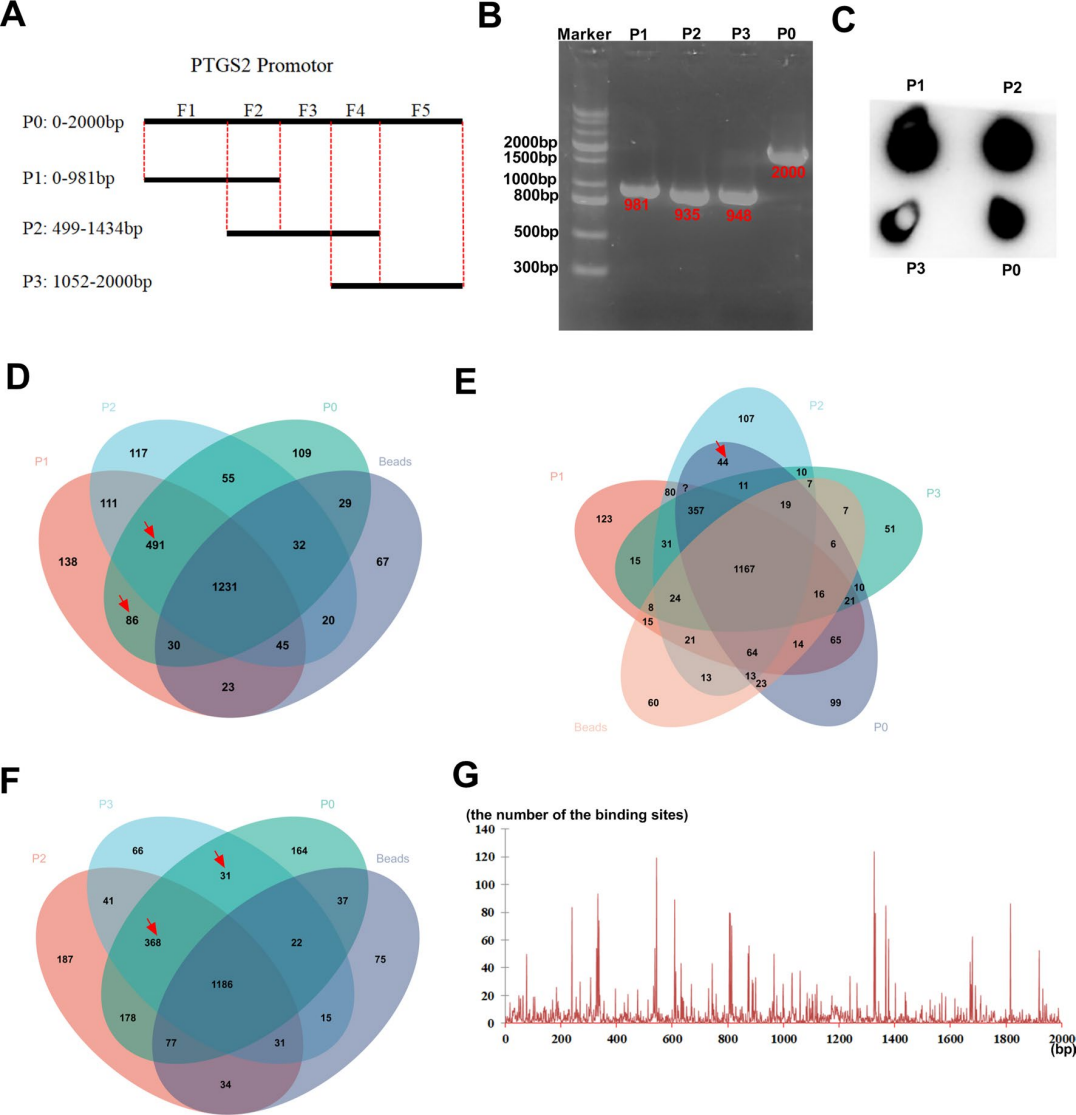
The two fragments (499–981 bp) and (1053–1434 bp) are the core TF binding profiles of the *PTGS2* promoter. (**A**) Schematic showing the four biotin-labeled sequences (P0, P1, P2 and P3) and the five fragments (F1, F2, F3, F4 and F5) of the *PTGS2* promoter. The five fragments of the *PTGS2* promoter (F1: 1–498 bp, F2: 499–981 bp, F3: 982–1052 bp, F4: 1053–1434 bp, and F5: 1435–2000 bp) were classified based on the four biotin-labeled sequences. (**B**) The biotin-labeled sequences (P0, P1, P2 and P3) were PCR-amplified using the *PTGS2* promoter sequence as template. The production of the four biotin-labeled sequences was validated by 1% agarose gel eletrophoresis. (**C**) Biotin labeling efficiency assay using an anti-biotin antibody conjugated by HRP with ECL Kit. (**D**–**F**) Venn diagram showing the specific proteins pulled down by the four biotin-labeled DNA sequences (P0, P1, P2 and P3) and beads in HCT116 cells. (**G**) A distribution histogram showing the number of the binding sites in each base of the *PTGS2* promoter predicted by JASPAR database.

To elucidate the precise TF binding regions of the *PTGS2* promoter, we firstly used PCR amplification with biotinylated primers to generate a biotinylated promoter full-length sequence (P0: 0–2000 bp) and three promoter truncations labeled by biotin (P1: 0–981 bp, P2: 499–1434 bp and P3: 1052–2000 bp) (Fig. 4A). The production of the four biotin-labeled sequences was validated by 1% agarose gel eletrophoresis (Fig. 4B) and a biotin labeling efficiency assay using an anti-biotin antibody conjugated by HRP (Fig. 4C). In the preliminary experiments, we found that HCT116 cells showed higher levels of PTGS2 mRNA than SW480 cells (data not shown), so HCT116 cells were selected for the subsequent mechanism assays. We then performed DNA pull-down assays: HCT116 CRC cells were lysed and incubated with each of the four biotin-labeled DNA sequences and streptomycin beads, followed by the collection of the precipitated proteins from the immunoprecipitates. Next, we analyzed the precipitated proteins using qualitative proteome profiling analysis by a HPLC–MS/MS method. Thousands of proteins were pulled down by each of the four biotin-labeled DNA sequences (P0, P1, P2 and P3) and beads (Supplementary Table 1). As illustrated in Fig. 4D, Venn diagram showed a total of 86 specific proteins pulled down by both P1 and P0 not P2 and beads (F1 fragment alone) and 491 specific proteins that overlapped among P1, P2 and P0 not beads (F2 fragment alone). Moreover, we found a total of 44 specific proteins pulled down by both P2 and P0 not P1, P3 and beads (F3 fragment alone), 368 proteins that overlapped among P2, P3 and P0 not beads (F4 fragment alone), and 31 proteins pulled down by both P3 and P0 not P2 and beads (F5 fragment alone) (Fig. 4E,F). These data indicated that the F1 fragment alone pulled down 86 specific proteins; the F2 fragment alone pulled down 491 specific proteins; the F3 fragment alone pulled down 44 specific proteins; the F4 fragment alone pulled down 368 specific proteins; and the F5 fragment alone pulled down 31 specific proteins. These proteins pulled down by each of the five fragments of the *PTGS2* promoter were shown in Supplementary Table 2.

Meantime, we used JASPAR database (https://jaspar.genereg.net/) to predict the TF binding profiles of the *PTGS2* promoter. Through a distribution histogram to show the number of the binding sites in each base of the *PTGS2* promoter, we found that the *PTGS2* promoter has more binding sites around these bases (240 bp, 340 bp, 540 bp, 610 bp, 810 bp, 1330 bp, 1380 bp and 1810 bp) (Fig. 4G), implying that these regions might be the core TF binding regions of the *PTGS2* promoter. By combining these specific proteins pulled down by each of the five fragments of the *PTGS2* promoter with the 1665 all human TFs from JASPAR database, we found that the number of TFs bound with each of the five fragments was 6 (F1), 49 (F2), 2 (F3), 43 (F4) and 4 (F5), respectively. These data together suggest that the core TF binding regions of the *PTGS2* promoter might be located in the F2 (499–981 bp) and F4 (1053–1434 bp) two fragments.

### PTGS2 expression positively correlates with the expression of TF RUNX1

**Figure 5:**
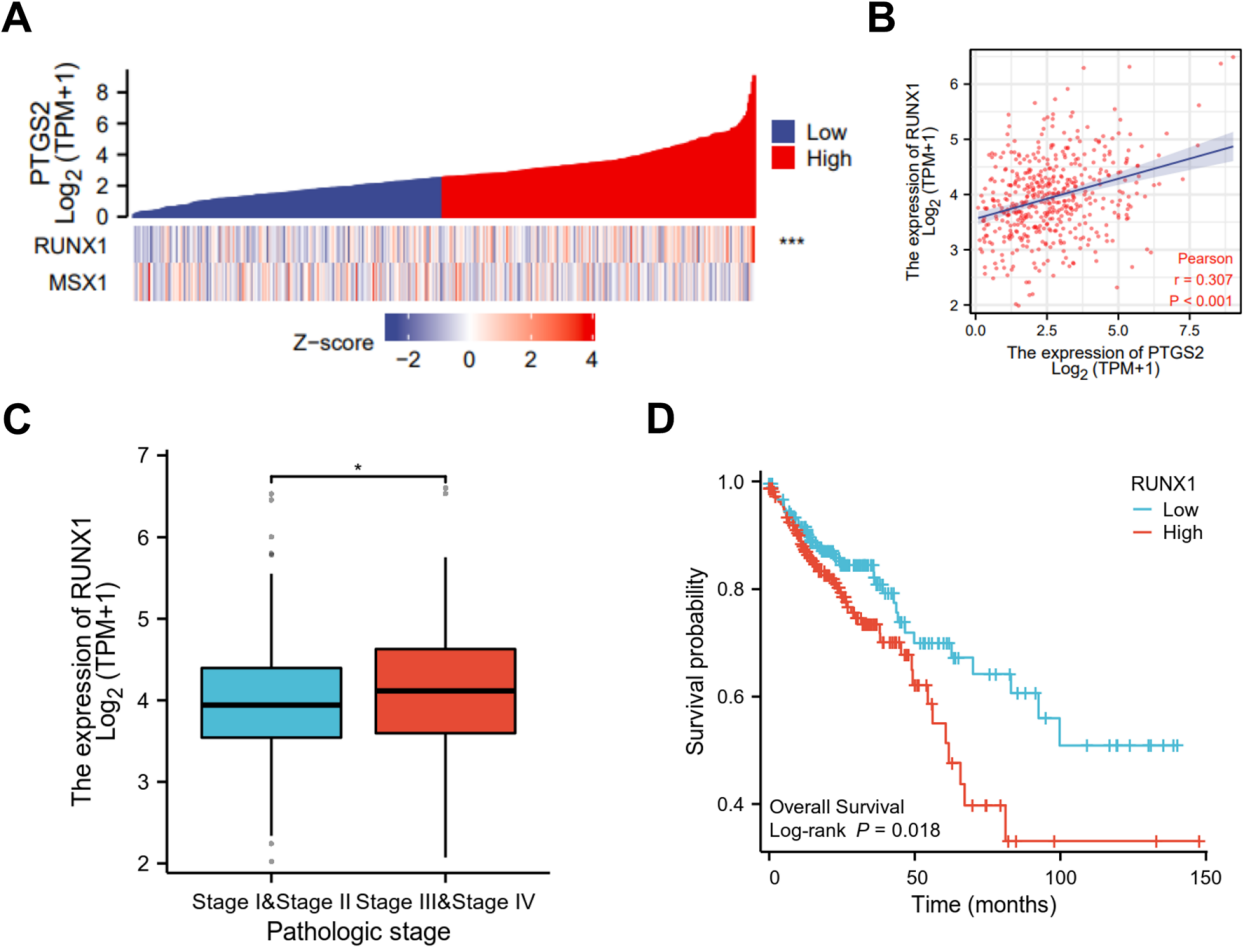
PTGS2 positively correlates with RUNX1. (**A**) Heat map showing a correlation between PTGS2 and RUNX1 or MSX1 expression using the TCGA-COAD dataset. (**B**) Scatter plots of PTGS2 expression versus RUNX1 level in 478 patients with COAD using the TCGA-COAD dataset. Pearson’s correlation coefficient (r) and *P* value are shown. (**C**) RUNX1 expression is associated with the pathologic stage of the COAD tumors. (**D**) Association between RUNX1 expression and the overall survival of these patients with COAD. *P < 0.05; ***P < 0.001.

Using KnockTF database (http://www.licpathway.net/KnockTF/) to obtain the known TFs in regulating PTGS2 expression, a total of 62 TFs was found based on the screen set threshold of log_2_FC > 1 (FC > 2) (Supplementary Table 3). To further study the TFs that regulate PTGS2 in HCT116 CRC cells, we combined the 62 known TFs with the TFs pulled down by the *PTGS2* promoter in HCT116 cells. Intriguingly, we found two TFs: RUNX1 and MSX1. To observe the correlation between PTGS2 and RUNX1 or MSX1 expression in CRC, we interrogated the TCGA-COAD dataset using National Cancer Institute GDC Data Portal (https://portal.gdc.cancer.gov/). Heat map showed a significant correlation of PTGS2 and RUNX1 expression in COAD samples, while no significant association was found between PTGS2 and MSX1 (Fig. 5A). Pearson’s correlation analysis also confirmed the significant and positive correlation between PTGS2 and RUNX1 expression in COAD samples (r = 0.307, *P* < 0.001; Fig. 5B). Furthermore, according to the findings reported by Li et al*.*^29^, RUNX1 expression was significantly associated with the pathologic stage of the tumors (Fig. 5C), and the high expression of RUNX1 predicted a poorer prognosis of these patients with COAD (Fig. 5D).

### RUNX1 acts as a positive regulator of PTGS2 expression by increasing the transcriptional activation of the *PTGS2* promoter

**Figure 6:**
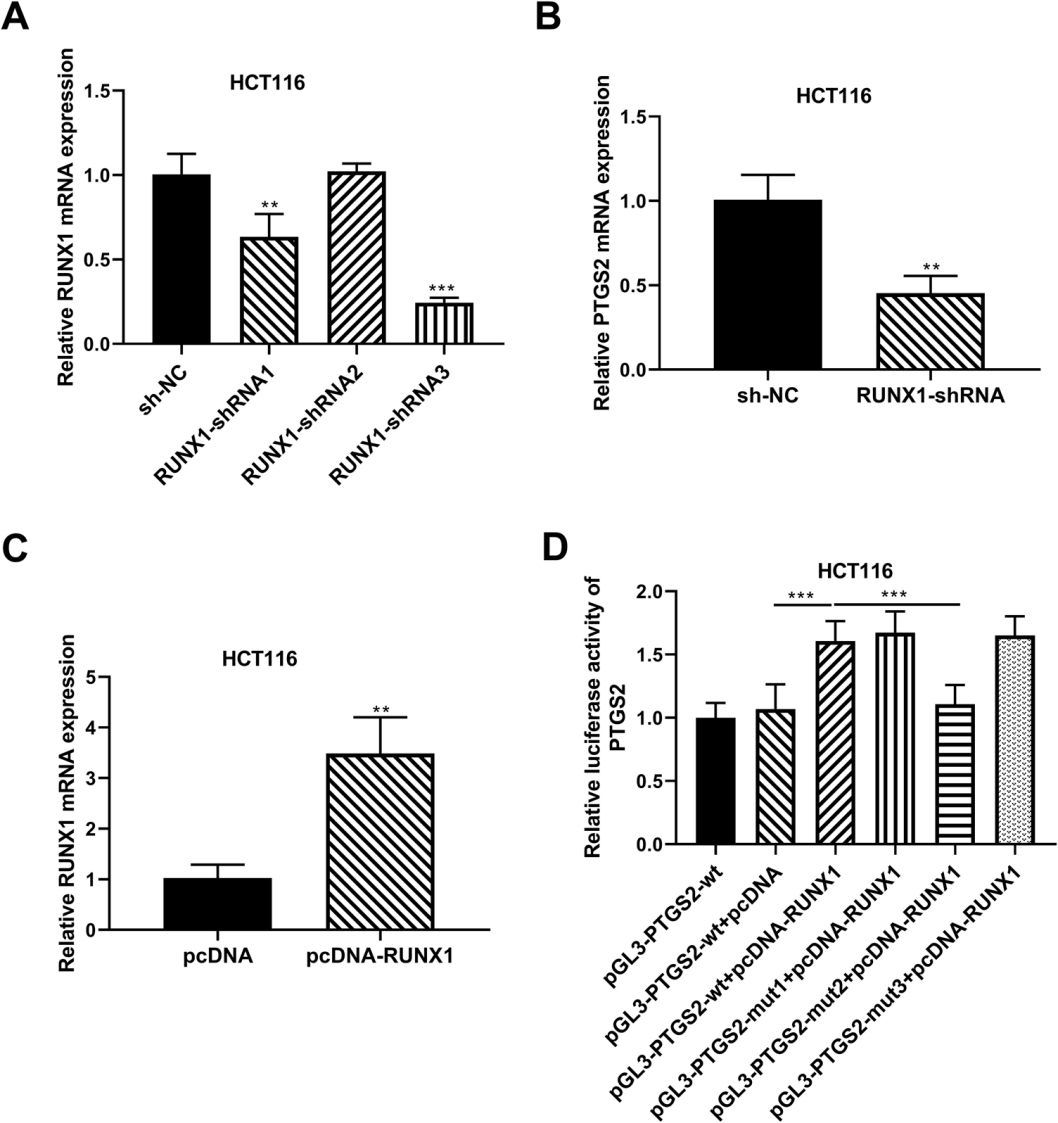
RUNX1 increases PTGS2 expression by binding to the *PTGS2* promoter and promoting its transcriptional activation. (**A**) qRT-PCR of *RUNX1* mRNA expression in HCT116 cells transfected with sh-NC, RUNX1-shRNA1, RUNX1-shRNA2 or RUNX1-shRNA3. Using *GAPDH* as a reference gene, relative expression of *RUNX1* mRNA was calculated by the 2^−ΔΔCt^ method. (**B**) qRT-PCR of *PTGS2* mRNA level in HCT116 cells after transfection by sh-NC or RUNX1-shRNA3. Using *GAPDH* as a reference gene, relative expression of *PTGS2* mRNA was calculated by the 2^−ΔΔCt^ method. (**C**) qRT-PCR of *RUNX1* mRNA expression in HCT116 cells transfected with pLV3-NC or pLV3-RUNX1. Using *GAPDH* as a reference gene, relative expression of *RUNX1* mRNA was calculated by the 2^−ΔΔCt^ method. (**D**) The wild-type (wt) and mutant-type (mut) PTGS2 luciferase reporter plasmids were constructed and transfected into HCT116 cells with pRL-TK *Renilla* control vector and pLV3-RUNX1 or pLV3-NC, followed by the analysis of luciferase activity. Firefly luciferase activity was normalized to *Renilla* activity and expressed as relative luciferase activity. n = three independent experiments performed in triplicate. **P < 0.01; ***P < 0.001.

Further, we asked whether RUNX1 regulates PTGS2 expression in HCT116 cells and, if so, how. To address this, we manipulated RUNX1 expression with three shRNAs targeting RUNX1 (RUNX1-shRNA) or a RUNX1 expression plasmid (pcDNA-RUNX1) in HCT116 cells. Transfection of RUNX1-shRNA1 or RUNX1-shRNA3, but not RUNX1-shRNA2, remarkably suppressed the expression of *RUNX1* mRNA in HCT116 cells (Fig. 6A). Since RUNX1-shRNA3 led to the most significant downregulation in *RUNX1* mRNA expression (Fig. 6A), we selected it for the subsequent research. Inhibition of RUNX1 by RUNX1-shRNA3 introduction significantly repressed cell growth, PCNA expression, migration, invasion, and MMP9 expression in CRC cells (Supplementary Figs. 3 and 4). Additionally, reduced expression of RUNX1 led to enhanced cell apoptosis (Supplementary Fig. 2). These findings indicated that RUNX1 knockdown in HCT116 and SW480 CRC cells can recapitulate the same phenotype observed upon PTGS2 knockdown. Interestingly, we observed a clear reduction in the level of *PTGS2* mRNA in RUNX1-silenced HCT116 cells (Fig. 6B), indicating that RUNX1 positively regulated PTGS2 expression.

Our DNA pull-down results showed that RUNX1 was pulled down by the F2 (499–981 bp) and F4 (1053–1434 bp) fragments of the *PTGS2* promoter (Supplementary Table 2), suggesting that RUNX1 might bind to the *PTGS2* promoter via the two regions. Using JASPAR database to predict the binding sites between RUNX1 and the *PTGS2* promoter, we found that the F2 fragment has no potential binding sites, and the F4 fragment contains three potential binding sites (site 1, 1062–1072 bp: AATTGAGGTAC; site 2, 1086–1096 bp: ATTTCAGGTTT; site 3, 1373–1383 bp: TATTGGGGCTA). To validate the binding of RUNX1 to the *PTGS2* promoter, we cloned the F4 fragment of the *PTGS2* promoter into the PGL3 vector (pGL3-PTGS2-wt) and analyzed its luciferase activity in the presence of pcDNA-RUNX1. Elevated expression of RUNX1 upon pLV3-RUNX1 transfection, confirmed by qRT-PCR (Fig. 6C), enhanced the luciferase activity of the PTGS2 luciferase reporter in HCT116 cells (Fig. 6D), revealing that RUNX1 can enhance transcriptional activation of the *PTGS2* promoter. To elucidate the validity of the three binding sites, we next generated three corresponding mutations (pGL3-PTGS2-mut1, pGL3-PTGS2-mut2 and pGL3-PTGS2-mut3) in each of the three potential sites, respectively. Luciferase assays revealed that site-directed mutation of the binding site 2 significantly abolished the promotion of RUNX1 overexpression in PTGS2 luciferase activity, while the two mutations in binding site 1 and 3 did not affect the effect of RUNX1 (Fig. 6D), demonstrating that transcriptional activation of the *PTGS2* promoter by RUNX1 is dependent on the 1086–1096 bp position of the F4 fragment. Together, these results establish that RUNX1 can enhance PTGS2 expression in HCT116 cells by promoting transcriptional activation of the *PTGS2* promoter via the 1086–1096 bp binding motif.

## Discussion

CRC frequently arises via the progressive accumulation of dysregulation in key genes including oncogenes and anti-tumor genes^34^. Targeting driver pathways would provide a better opportunity to design more efficient therapeutic interventions for CRC^35,36^. In the current report, our shRNA silencing experiments confirmed the oncogenic activity of PTGS2 in CRC. Furthermore, we defined RUNX1 as a potent TF activator of PTGS2 expression in CRC cells. We therefore provide evidence that elevated expression of PTGS2 induced by RUNX1 contributes to colorectal tumorigenesis.

Numerous studies have demonstrated the crucial implication of PTGS2 in the carcinogenesis of CRC^18,19^. For instance, glycosylated PTGS2, a stable PTGS2 form, is prevalently expressed in CRC tumors with high sensibility^37^. The polymorphism of *PTGS2* is related to the risk of CRC^38,39^. Enhanced expression of intestinal PTGS2 is reported to be an early event in colorectal tumorigenesis^39^. Moreover, PTGS2 upregulation is associated with poorer CRC-specific survival^16,17,40^. Therefore, PTGS2 has been proposed as a promising target for CRC prevention and treatment^41,42^. In this paper, loss-of-function phenotypes of PTGS2 by sh-PTGS2 led to a decrease in CRC cell growth, migration and invasiveness in vitro, suggesting that PTGS2 is a promoting regulator of CRC cell malignant behaviors. A recent study shows that PTGS2 does not affect CRC cell differentiation and metastasis in vivo^43^. These contradictory conclusions may be attributed to more complex in vivo microenvironments relative to in vitro conditions.

Previous reports indicate that some TFs, such as Ets-1 and KLF4, are able to enhance the expression of PTGS2 by elevating the transcriptional activation of the *PTGS2* promoter^44,45^. Moreover, TFs Sp1 and NRF2 can induce PTGS2 expression in cancer cells^46,47^. On this basis, we here focused on investigating the TFs that regulate PTGS2 expression in CRC cells. Using DNA pull-down assay and qualitative proteome profiling analysis, we firstly elucidated the TF binding regions of the *PTGS2* promoter. Our data indicated that the two fragments (499–981 bp and 1053–1434 bp) of the *PTGS2* promoter can bind more TFs than other sequences, suggesting that the two fragments might be the core TF binding profiles of the *PTGS2* promoter. Using KnockTF database, RUNX1 seems to be a TF involved in the regulation of PTGS2 expression in CRC cells. Interestingly, we also found a significant positive correlation between PTGS2 and RUNX1 expression in COAD samples. In rat periovulatory granulosa cells and leukemic stem cells, RUNX1 can induce PTGS2 expression by increasing transcriptional activity of the *PTGS2* promoter^48,49^. Similarly, we experimentally showed that RUNX1 operates as a positive regulator of PTGS2 expression in HCT116 CRC cells by promoting transcriptional activation of the *PTGS2* promoter via the 1086–1096 bp binding motif. On the other hand, suppression of RUNX1 can lead to an increase in PTGS2 expression in myofibroblasts^50^. Recent work has uncovered the double-edge role of RUNX1 in the progression of solid tumors by functioning as an oncogenic driver or a tumor suppressor^26^. These contradictory findings may be in part due to the different tumor types. More interestingly, in CRC, RUNX1 is overexpressed and has been identified as a potent driver in colorectal tumorigenesis^28,51^; conversely, RUNX1 is reported to work as an anti-cancer factor in gastrointestinal malignancies^52,53^. Our data suggested the oncogenic role of RUNX1 in CRC (Supplementary Figs. 2–4). These controversial findings may be due to microsatellite instability statues and different microenvironments of gastrointestinal tumor tissues.

Inhibitors of PTGS2 have been proposed as potential agents for CRC prevention and treatment^15,41,42^. Nevertheless, no major clinical trials of PTGS2 inhibitors were completed in CRC due to their adverse effects, such as elevated risk of myocardial infarction, dyspepsia, abdominal pain, gastrointestinal bleeding, and gastritis^19,54^. Moreover, it remains unclear how to prevent the potential adverse effects of PTGS2 inhibitors and which CRC patients would benefit the most from these inhibitors. To find more effective targets for PTGS2 inhibition, we investigated the upstream mechanism of PTGS2 regulation in this study and demonstrated that TF RUNX1 is a positive regulator of PTGS2 expression. Our data indicated the reduced effect of RUNX1-shRNA (sh-RUNX1) on PTGS2 level in HCT116 CRC cells. With these findings in our study, the sh-RUNX1 vector appears to represent a potential anti-CRC agent that might function as a suppressor on tumor growth and metastasis in CRC by silencing PTGS2. We envision that RUNX1 inhibitors may have the potential to improve the outcome of patients with CRC. Further work will build on these findings by determining the long-term efficacy and safety of such inhibitors in various experimental models.

In summary, we define that TF RUNX1 enhances PTGS2 expression by elevating transcription activation of the *PTGS2* promoter via the 1086–1096 bp binding motif, and as a consequence, promotes CRC cell growth, migration and invasion. Emerging from our study is an increased rationale for the use of PTGS2 inhibitors in CRC prevention and treatment.

### Supplementary Information

Supplementary Information.

## Data Availability

The data and material presented in this manuscript is available from the corresponding author on reasonable request.
