# Supplementary material for: RUNX1-induced upregulation of PTGS2 enhances cell growth, migration and invasion in colorectal cancer cells
Source: Sci Rep. 2024 May 22;14:11670. doi: 10.1038/s41598-024-60296-z (PMC11111780; doi:10.1038/s41598-024-60296-z)
Supplement: Supplementary file 1 — Supplementary Information. [file 41598_2024_60296_MOESM1_ESM.docx]

**Supplementary information**


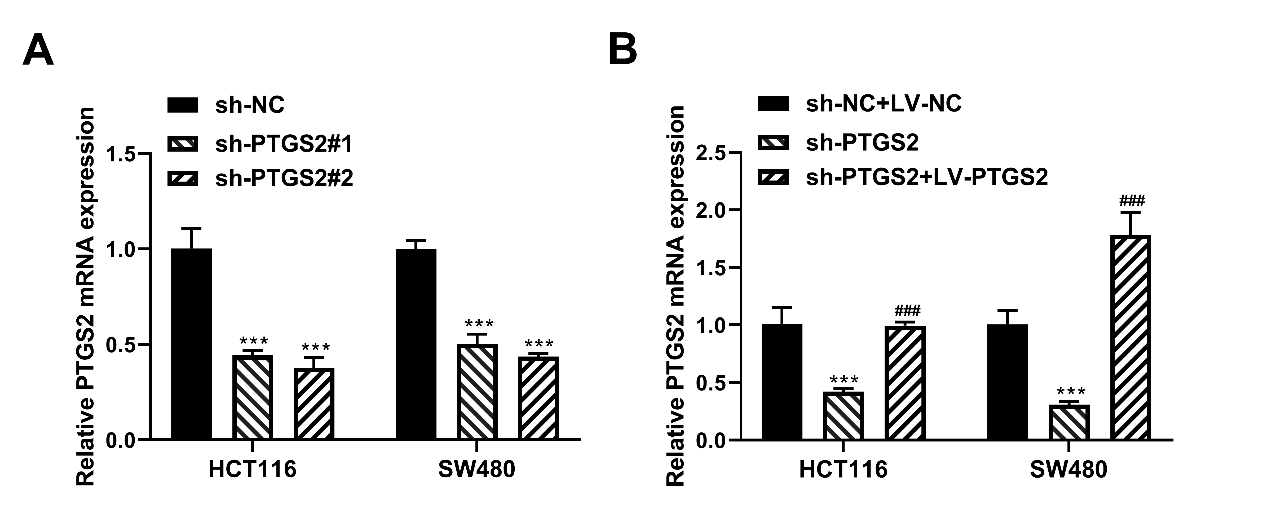


**Supplementary Figure 1.** Detection of transfection efficiency. (A and B) qRT-PCR of *PTGS2* mRNA level in HCT116 and SW480 CRC cells transfected with sh-NC, sh-PTGS2#1, or sh-PTGS2#2 (A), sh-NC+LV-NC, sh-PTGS2#2, or sh-PTGS2#2+LV-PTGS2 (B). ****P*<0.001, ^###^*P*<0.001.


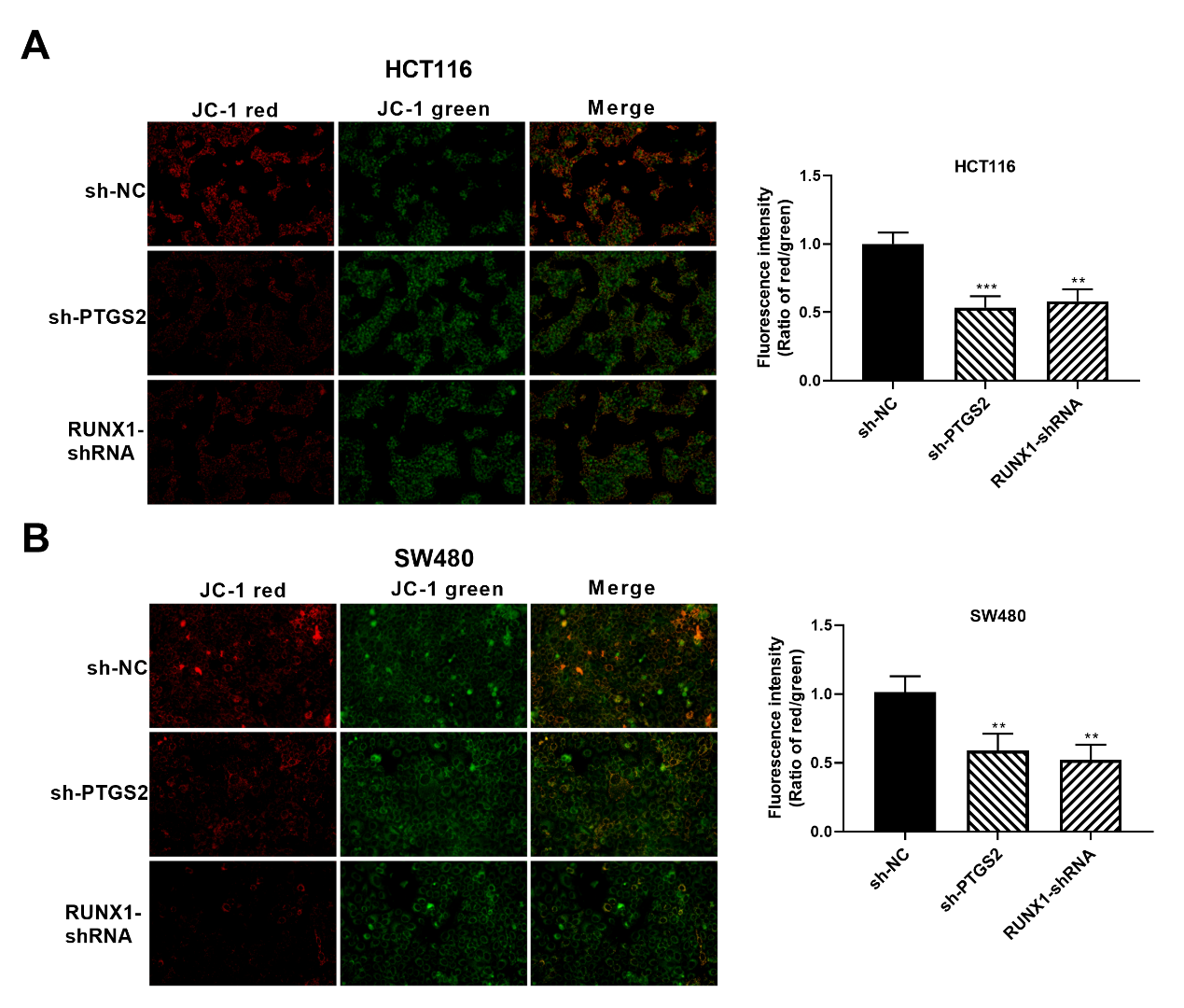


**Supplementary Figure 2.** Evaluation of apoptosis of transfected CRC cells. (A and B) HCT116 and SW480 CRC cells were transfected with sh-NC, sh-PTGS2, or RUNX1-shRNA3, followed by the assessment of cell apoptosis using JC-1 mitochondrial membrane potential assay. Images of three random fields were analyzed by ImageJ, and the ratio of red fluorescence/green fluorescence was determined. ***P*<0.01, ****P*<0.001.


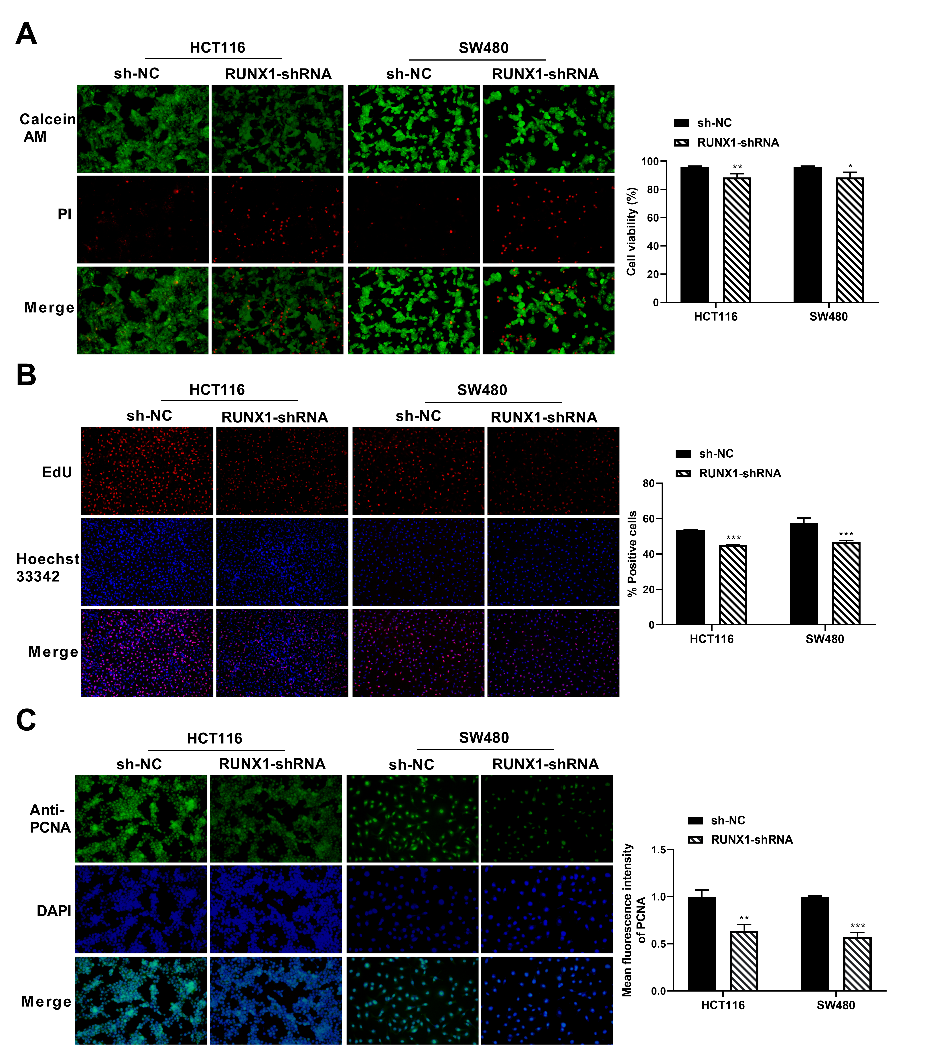


**Supplementary Figure 3.** RUNX1 silencing represses the growth of HCT116 and SW480 CRC cells. (A) Cell viability assay was performed with HCT116 and SW480 CRC cells after transfection by sh-NC or RUNX1-shRNA3 using Clacein/PI Cell Viability/Cytotoxicity Assay Kit. The number of stained cells was quantified by ImageJ and cell viability was calculated by using the method: cell viability (%) = (number of Calcein AM^+^ cells)/(number of Calcein AM^+^ cells+number of PI^+^ cells)×100. (B) EdU assay for cell proliferation performed with HCT116 and SW480 CRC cells transfected with RUNX1-shRNA3 or sh-NC. Images of three random fields each sample were captured using a fluorescence microscope and the EdU positive cells (%positive cells) were defined as a percentage of total nuclei. (C) Immunofluorescence assay showing PCNA fluorescence intensity in cell lines transfected as indicated. Images of three random fields were obtained and the fluorescence intensity was quantified by ImageJ to get an average fluorescence intensity of PCNA. **P*<0.05, ***P*<0.01, ****P*<0.001.


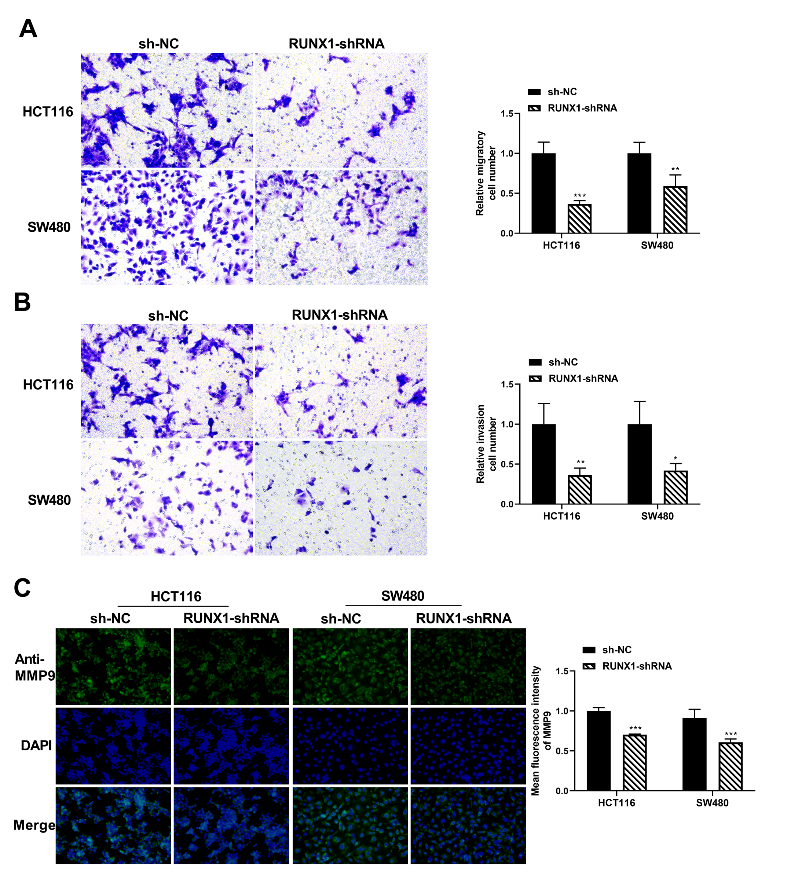


**Supplementary** **Figure 4.** RUNX1 silencing inhibits the migration and invasion of HCT116 and SW480 CRC cells. (A and B) Transwell migration and invasion assays of HCT116 and SW480 CRC cells transfected with RUNX1-shRNA3 or sh-NC. Transfected cells were seeded on 24-transwell inserts and translocated toward the complete growth medium. After 48 h of culture, pictures of at least three random fields from three replicate wells were obtained by a 100× magnification microscope and the number of the migratory and invaded cells was quantified by ImageJ. (C) Immunofluorescence assay showing the fluorescence intensity of MMP9 in cells after transfection by RUNX1-shRNA3 or sh-NC. Cells were incubated with anti-MMP9 antibody and secondary antibody. Cell nucleus was stained with DAPI. Images of three random fields were obtained and the fluorescence intensity was quantified by ImageJ to get an average fluorescence intensity of MMP9. **P*<0.05, ***P*<0.01, ****P*<0.001.

| **Supplementary Table 1. The proteins pulled down by each of the four biotin-labeled DNA sequences and bead** | | | | |
| --- | --- | --- | --- | --- |
| P1（0-981bp） | P2（499-1434bp） | P3（1052-2000bp） | P0（0-2000bp) | Beads |
| SQOR | ZNF451 | RBM47 | TM7SF2 | PDS5B |
| GEMIN5 | PQBP1 | EIF4A2 | ACSL4 | ZMYND8 |
| KIF2C | FLII | SIX4 | SCAF1 | MBNL1 |
| TAF7 | SIX4 | THOC3 | USP34 | DRG1 |
| VPS29 | THOC3 | NUDT5 | CLOCK | EIF3B |
| NPLOC4 | MRRF | EIF3L | MRRF | ZNF131 |
| OTUB1 | PFDN2 | HLA-C | JUN | EXOSC3 |
| RTCA | GRSF1 | CCDC59 | PRKAA1 | CAMK2A |
| NDUFB9 | SQOR | SREK1 | PATZ1 | LRRC41 |
| KNOP1 | NKAP | DPM1 | HOMEZ | NDUFS4 |
| TADA1 | ZMYND8 | SENP3 | RBM34 | DSC3 |
| BUD23 | SUPT6H | TOP1MT | KAT8 | SGF29 |
| DTD1 | RPRD1A | PNISR | MAD1L1 | UTP4 |
| ZMYM3 | POM121C | UTP3 | NOSIP | RAB1A |
| DAXX | CLASP1 | SRSF11 | PQBP1 | SP2 |
| MED24 | ATP6V1G1 | CELF1 | YARS2 | DDX39A |
| USP48 | COX5B | USP34 | OCIAD2 | MRPS21 |
| AKR7A2 | CCNT1 | CCNT2 | MRPL14 | GAK |
| ATF1 | UCHL5 | ALDH2 | LRRC1 | PALM |
| NKAP | SPATS2L | HOMEZ | ELF1 | DHX38 |
| LRBA | CDCA2 | DGCR8 | PARK7 | GADD45GIP1 |
| CUL3 | TUBAL3 | ACADVL | PTRH2 | EMC1 |
| SGF29 | SCAMP3 | EXOSC3 | ZNF512B | CCDC12 |
| DNM1L | NAB2 | DDX6 | EEF1B2 | IL1RL1 |
| FH | NFKBIL1 | MRPS18B | MRPL22 | LSM12 |
| ZNF146 | KIF20A | ITGB4 | COX5B | RPRD1B |
| ANKRD11 | TAF3 | KRT15 | ENAH | KIF2A |
| AGPS | UTP11 | POLR1C | QSER1 | GSN |
| SREK1 | HSP90AB2P | MTHFD1L | CPT1A | NFIL3 |
| EVPL | DNAJC11 | CENPV | SIX4 | MCRS1 |
| AARS1 | SMC5 | CCDC12 | MRPL39 | TADA1 |
| NOP53 | STAT3 | G3BP2 | POM121C | PPP3CB |
| XPO4 | TMEM11 | EMC1 | PKP2 | KANSL3 |
| LSM6 | ECSIT | BTF3 | NAA50 | HSD17B12 |
| NT5DC2 | ARHGEF1 | CISD1 | GPRC5C | NVL |
| CCNT2 | TADA1 | PSMC3 | PLEKHS1 | KIF2C |
| THOC3 | CPNE8 | VAPB | THOC3 | PRRC2A |
| PKP1 | ZNF16 | DHX16 | ATF1 | C1QBP |
| NTPCR | EVPL | EPB41L2 | PACSIN3 | SREK1 |
| USP13 | MRPL32 | RACGAP1 | KIF2C | QSER1 |
| MYL6 | ANKRD11 | TMEM126A | UTP3 | ISG20L2 |
| NFIB | EPPK1 | POLR2C | MYADM | RSBN1 |
| NUFIP1 | EIF2A | PALM | PDIA5 | FZR1 |
| MED6 | UTP20 | SUPT3H | NMT1 | CANX |
| SERPINB12 | MAU2 | DSC2 | CLASP2 | CKB |
| PPA1 | ERI1 | KLF13 | AKR1A1 | RBBP7 |
| RAB18 | PLCD3 | SMC2 | RAP1B | SLC38A2 |
| PPID | PPP1R8 | PKN3 | AHSA1 | MGST1 |
| SMIM4 | SPCS3 | MCCC1 | NR2F1 | TAF1C |
| UBE2I | PPP2R2A | SLC25A6 | ZC3H15 | AHDC1 |
| LARP1 | TIMM44 | MRPS31 | MARS1 | TADA3 |
| SH3PXD2B | PPA1 | CKMT1A | NUFIP1 | GSDMA |
| SP2 | UMPS | CDR2L | RMDN3 | TSPYL1 |
| PRKAR2A | GDI1 | MAD1L1 | RPRD1B | EHD1 |
| MCCC2 | KARS1 | TSFM | KRT84 | TAF5 |
| SIX4 | APOC3 | STAU1 | TMEM205 | MYO1G |
| STXBP1 | USP30 | PSMD3 | PRORP | ZBTB20 |
| TRIM47 | PRORP | KNOP1 | TWNK | CMAS |
| FBXO28 | MAVS | USP48 | KMT2A | POLR2H |
| UMPS | G3BP2 | PPP2CB | YBEY | CDC42BPB |
| CUSTOS | TBC1D24 | CABIN1 | NAA10 | TCERG1 |
| GOT1 | LUZP1 | SLC25A13 | PYGL | MSI1 |
| SMPDL3B | KDM5A | SORD | UTP11 | PNISR |
| PRKAA1 | TSR1 | UTP6 | MAZ | RBM47 |
| PATZ1 | NUFIP2 | HSPH1 | TAF3 | LEO1 |
| PRKCD | NTPCR | EIF4H | GSK3B | FBXL12 |
| AP3D1 | PHF3 | KAT7 | SEC61B | RPS28 |
| YARS2 | TJP2 | BUD13 | RDH11 | ORC3 |
| CCDC51 | LSR | POLR1F | AMOTL1 | KDM2A |
| UTP11 | CENPV | HSPE1 | GSK3A | USP3 |
| TSR1 | AFDN | CIRBP | TAF1C | RCOR1 |
| RAVER2 | POLG | RAP1B | JRKL | IRS1 |
| VPS26A | DSTN | PTBP3 | KIAA1671 | TMED10 |
| SH3GL1 | RAB5A | CLASP2 | SCAF4 | PCNA |
| SAR1A | DCAF1 | TOX2 | RSBN1 | GNA13 |
| PNISR | INO80B | PSMC6 | INO80 | MRPL13 |
| AFDN | RFX1 | ZNF451 | POLR1F | EP400 |
| NAB2 | SMC4 | UTP15 | SEPTIN8 | MPHOSPH8 |
| AP2S1 | PHLDB1 | XPO4 | TJP2 | ARL6IP4 |
| ZCCHC7 | RAVER2 | DNAJB11 | LSR | EPHA2 |
| TMEM201 | BEND3 | RAD50 | UBE2I | NCCRP1 |
| SLC25A13 | STK24 | DENR | EVPL | PIP5K1A |
| RRAS2 | RAB8A | EPHA2 | MEPCE | LLPH |
| L3MBTL2 | PIP5K1A | H2AW | PNKD | NSMCE4A |
| ZDHHC5 | NAP1L1 | ANGEL1 | FKBP5 | TBK1 |
| SCAF1 | AKR7A2 | CLINT1 | TRIP13 | MED14 |
| TBK1 | LZTS2 | DRG1 | TGM1 | CEP170 |
| MED14 | LLPH | ZNF143 | MED24 | PTBP3 |
| UTP15 | SIK3 | CDX2 | MBOAT7 | NIPSNAP1 |
| CLASP2 | IMPA1 | RAI1 | ZBTB10 | TMEM33 |
| LSR | PREP | URB2 | LYPLAL1 | MSL1 |
| LRRC47 | DSC1 | PGD | ZMYM3 | SERPINH1 |
| H2AW | SMNDC1 | MAOB | CEBPB | LIN7C |
| ZBTB9 | MRPS5 | DOCK7 | SRSF11 | RCOR2 |
| UTP6 | RIF1 | ALDH7A1 | CDK12 | ACTA1 |
| COPB2 | DDX6 | PLCD3 | RRAS | KHDRBS1 |
| DHX8 | NVL | PUM2 | KANK2 | HNRNPH3 |
| PPFIA1 | HEXIM1 | CCDC180 | LEMD2 | RPL8 |
| CAAP1 | CACYBP | SLC3A2 | UBE2N | TJP1 |
| MVD | BLMH | SUPT20H | TXNRD1 | RPS14 |
| SUPT20H | CORO1A | TMEM33 | NSMCE4A | JUP |
| NEIL2 | GNAQ | GAK | NAP1L1 | RPL26 |
| PPP2R2A | POTEE | PSMA1 | PRKAR1A | RPS8 |
| TSPYL1 | MVD | TBK1 | SH3PXD2B | EBNA1BP2 |
| WDR6 | CCNT2 | KRTCAP2 | PPIE | TUBB4B |
| CDCA2 | UBN1 | SHOC2 | DENR | RPL21 |
| PALM | SH3PXD2B | IPO5 | IPO5 | CDC5L |
| ASNS | MRPS33 | NUFIP1 | CSRP2 | RFC5 |
| HPF1 | ABI2 | NFRKB | UTP6 | RUVBL1 |
| LSM3 | RBM34 | RCOR2 | KLK7 | PABPC1 |
| NCOR2 | KIF2C | KLF5 | EMC1 | H1-10 |
| ELF2 | SEC13 | C17orf80 | ORC1 | VDAC1 |
| IKBIP | CLASP2 | PKP1 | FBXO11 | SLC2A1 |
| USP34 | ST14 | POLR2H | PAWR | OAT |
| SRSF11 | FARP1 | FAM98A | XPO4 | CBX8 |
| SAE1 | MBNL1 | LSM12 | ECM1 | MCM4 |
| FYTTD1 | ATP6V1A | SERPINB1 | MRPL23 | SMARCE1 |
| MBOAT7 | AGFG1 | GPD2 | APOC3 | POGZ |
| MAP2K3 | EMC1 | GTF2H4 | AARS1 | RPL37A |
| ZNF451 | MRPS25 | TADA1 | C17orf80 | NR2F1 |
| AK1 | MRPL39 | HSPA4 | MRPS21 | HNRNPAB |
| PARVA | CAAP1 | ZMYM3 | PRCC | RPRD2 |
| MCTS1 | MRPS30 | COPB1 | MACROH2A2 | NDUFS1 |
| MDH1 | DGCR8 | GSN | EMILIN1 | NME2 |
| HLTF | FAR1 | HNRNPF | STK39 | H2AC21 |
| DDX3X | RAB21 | KRT19 | ASH2L | NUP188 |
| H1-0 | LRRC1 | HNRNPA0 | GDI1 | HMGB2 |
| CLTC | ENPEP | SF3B2 | TNKS1BP1 | INTS3 |
| NUMA1 | CUL4B | TFAM | ACTR5 | NOL9 |
| TOP2B | LRRC47 | MYH9 | OSBPL8 | CDC23 |
| MDC1 | SRCAP | SF3B1 | MSL2 | NOL10 |
| CTNNB1 | MED14 | HSP90AB1 | EXOC8 | RBM6 |
| ALDH18A1 | TBK1 | DDX17 | TSFM | NGDN |
| RPL7A | CDC37 | VDAC2 | NUP214 | CCT3 |
| SRSF9 | CTNNBL1 | ACACA | LZTS2 | ALDH3A2 |
| RPS13 | USP48 | CLTC | BCAS2 | IMP3 |
| SART1 | RAB25 | HSPA1B | MRPS25 | UTP14A |
| ADAR | DAD1 | ACTR3 | SMARCD1 | SUN1 |
| TKT | SRSF11 | RPA2 | L3MBTL2 | TUBA1A |
| PRPF19 | AHNAK2 | TIAL1 | INO80B | LARP7 |
| SNRPD3 | TXNL1 | EXOSC10 | GRHPR | KIF4A |
| RFC3 | APOO | CBX8 | SPCS3 | RBM22 |
| CTNNA1 | CDC42BPG | S100A4 | CTNNBL1 | BRD7 |
| TRA2B | TFDP1 | MT-CO2 | PPP1R8 | ATP5PB |
| KIF22 | C8orf33 | ST13P4 | PREP | INTS14 |
| SF3A3 | CDK12 | RPL14 | PLCD3 | SEC13 |
| EEF1G | YLPM1 | HMGA2 | ZC3H4 | POLR2C |
| SERBP1 | HNRNPUL2 | PRPF40A | ZC3H13 | TXN |
| PGAM5 | U2SURP | DDX41 | LSM3 | PWP1 |
| RRBP1 | SF3B1 | TCOF1 | SLC4A1AP | ZC3H14 |
| WDR33 | PRSS1 | WDR33 | SPATS2L | PHF8 |
| RBM4 | HP1BP3 | COX4I1 | GPKOW | PCID2 |
| HRNR | SMARCA5 | EIF4A1 | PPL | FLOT1 |
| HDAC1 | ATP5F1B | NIPBL | EML2 | KIAA1671 |
| PRPF4 | TFAM | RBM25 | MCTS1 | GPATCH4 |
| RPL38 | NOLC1 | SNRPE | DHX16 | SAP30BP |
| BSG | RPL13 | DDX18 | SLC25A32 | PSMD11 |
| DDX24 | PHB1 | SRP14 | CAAP1 | ANXA1 |
| DDX18 | TIAL1 | NDUFS1 | EP400 | TAF2 |
| CYC1 | CTNND1 | PES1 | CUL3 | GLYR1 |
| PGAM1 | TCF7L2 | SMARCE1 | RFX5 | SNRPB2 |
| MISP | RFC1 | SNU13 | SLC25A36 | MOGS |
| RBM27 | RPLP0 | ADNP | CBX4 | PPIA |
| LLGL2 | RPL4 | RRS1 | NOP53 | SUMO1 |
| BLM | SNRPA1 | FTSJ3 | ARL6IP4 | PSMD13 |
| KRT74 | PKP3 | ACTR8 | ZDHHC5 | NEPRO |
| INTS3 | NUP210 | NAP1L1 | ACTB | PHF2 |
| YY1 | DBN1 | CHTOP | PRKDC | YME1L1 |
| SMCHD1 | NUP155 | CMAS | ILF3 | CHD8 |
| PSMB5 | MDH2 | STOML2 | EZR | CXXC1 |
| FXR1 | HSPB1 | EHMT1 | NOP56 | MED17 |
| CTBP2 | EEF1G | SUN1 | SFPQ | EIF2S2 |
| KRAS | PCBP2 | SRPRB | SF3B2 | NFIA |
| SIN3A | RBM15 | CSNK2A1 | TRIP12 | FAM98B |
| CCAR2 | YWHAE | SUGP2 | SNRPB | MLF2 |
| RBM3 | KPNA4 | LENG8 | RPL14 | KIF20A |
| ALDH1A3 | API5 | RPF2 | TCP1 | ATP13A1 |
| CYB5B | PC | MTX1 | HMGB3 | ERAL1 |
| NOL6 | RPL28 | DSG1 | DDX23 | ORC1 |
| ANXA3 | CCAR2 | ORC5 | OAT | NIPSNAP2 |
| MCM6 | PACSIN2 | CAPZB | LIMA1 | SLC25A12 |
| CNN3 | ASPH | PRDX6 | SPEN | ZC3H13 |
| ACSL5 | ALDOC | RPL36AL | MT-CO2 | CCNB1 |
| EMG1 | RRP9 | ALDH3A2 | RPL10 | ST13P4 |
| RAB14 | CMAS | STAG1 | MAGOH | MOV10 |
| PYCR2 | XRCC1 | NUP37 | RCC1 | RPS3A |
| PHF6 | CKAP4 | DDX50 | SMARCC1 | ACTB |
| ILKAP | DDX51 | HSPA4L | RFC1 | KRT1 |
| PLCB3 | TUBG1 | MYO1D | TOMM40 | KRT10 |
| IPO7 | EIF5A | ACTR1A | RPL35 | KRT9 |
| INTS10 | EHMT1 | WDR18 | CPSF2 | ACTBL2 |
| PSMA6 | PPHLN1 | RBMS2 | VCP | KRT2 |
| EIF4E | MAP4K4 | RAB14 | SAMD1 | HNRNPC |
| SUN2 | DNTTIP2 | GART | SET | PRKDC |
| POP1 | NHP2 | PLIN3 | RNPS1 | KRT8 |
| FOSL2 | CTCF | DMAP1 | PFKP | HNRNPK |
| NR2C2 | CHAMP1 | TUBA4A | RAB15 | NPM1 |
| NUP43 | OCIAD1 | KLF16 | CPSF7 | MATR3 |
| FAF2 | RBM27 | CDK12 | DNTTIP2 | ILF3 |
| RAB2B | ARF6 | BCAS2 | SMARCC2 | KRT18 |
| YES1 | TARS1 | POP7 | DDX47 | KRT19 |
| PUM1 | FXR1 | NCBP2 | CYCS | RFC4 |
| TCEA1 | PBRM1 | MRPS21 | CCDC47 | DDX21 |
| WTAP | NF1 | SRSF4 | RRP9 | TOP1 |
| RCOR1 | ZNF148 | CAP1 | TOR1AIP1 | HNRNPR |
| TERF2IP | MRTO4 | ZC3H4 | GTF3C5 | DHX9 |
| INTS7 | SCAI | MAL2 | DNAJB1 | SYNCRIP |
| NTHL1 | NME3 | PARD3 | SRRM1 | H1-3 |
| MRPL45 | GSTO1 | TENT4B | HK1 | CPSF2 |
| DDX50 | DCAF7 | TRMT1L | CD44 | RBM14 |
| CDC37 | PHF6 | ZNF746 | NCBP1 | H3-7 |
| PARD3 | FARSA | ARL6IP5 | MCM5 | PRSS1 |
| NOB1 | TFPT | WDR6 | YWHAB | HNRNPA2B1 |
| EIF3G | PGM5 | ZSCAN29 | NQO1 | ALB |
| MORC2 | SUZ12 | PPP1R12A | HTATSF1 | KRT5 |
| TBPL2 | PSMC4 | RHOF | CAPRIN1 | SNRNP200 |
| GRSF1 | CTSZ | CHD8 | DLD | ILF2 |
| CDYL | PSMA5 | SAR1A | SND1 | MYH9 |
| TBRG4 | LCN1 | DNAJC19 | RAB13 | SF3B3 |
| FABP5 | HDHD5 | PARN | KIF4A | LMNA |
| MRPL41 | EXOSC2 | ARL6IP6 | FAM98A | SF3B1 |
| ONECUT2 | TRIM25 | MTCH1 | ALDH1B1 | LMNB1 |
| CSNK1D | DHX33 | RPS3A | SMARCA1 | NUMA1 |
| TLN1 | OGDH | ACTB | AAAS | HNRNPL |
| SPOUT1 | PRKCSH | KRT1 | RPF2 | MKI67 |
| WDR18 | ITGB1 | TOP1 | PDS5B | DSP |
| MAD1L1 | PAK1IP1 | PARP1 | C1QBP | HNRNPH1 |
| NSF | DDX50 | HNRNPC | PSME1 | GAPDH |
| SLC25A12 | PGRMC1 | H4C1 | RGPD3 | PDCD11 |
| NR2F1 | MYL6 | HNRNPU | POLR2E | PRPF8 |
| REEP6 | TOMM22 | KRT10 | DCAF7 | PRPF6 |
| KDM2B | INO80 | ACTBL2 | KPNA3 | DDX3X |
| PHF8 | EIF3CL | RBM14 | PSMD3 | NOP2 |
| CDC42BPB | BAIAP2L1 | HMGA1 | DHX37 | HNRNPUL2 |
| MAPRE1 | AP3B1 | HNRNPA2B1 | LENG8 | KHSRP |
| EED | SUMO3 | HNRNPK | IMP3 | SRSF1 |
| ATXN2L | QARS1 | KHDRBS1 | SFN | RPS4X |
| TUBA4A | PSMB7 | KRT2 | HLA-A | RBM17 |
| MRPS21 | ZNF512B | PRKDC | FDFT1 | PLEC |
| EXOSC7 | NOMO2 | KRT8 | ZNF146 | U2AF2 |
| ABCF2 | HBS1L | LMNA | GLG1 | RPS18 |
| TAF6 | RBBP5 | ACTA1 | GSR | LMNB2 |
| HOXB9 | DPM1 | RBM12B | CEP170 | SF3B2 |
| CDC40 | WBP11 | MKI67 | ALDH7A1 | DDX5 |
| COX5B | STRAP | ALB | WDR74 | HNRNPF |
| MPHOSPH8 | NUDC | NPM1 | RPRD1A | RPS3 |
| RFX1 | C1orf174 | ILF3 | RSL24D1 | U2SURP |
| FAM98A | EIF2AK2 | MATR3 | NDUFA11 | RPL7A |
| BAIAP2L1 | MRPS27 | HNRNPD | EIF3D | FBL |
| TAOK1 | BUD31 | HNRNPH3 | NMNAT1 | HP1BP3 |
| CAPZA1 | PTGES | HNRNPM | ZNF644 | RANBP2 |
| ARL1 | RAC2 | SSBP1 | RCOR1 | NAT10 |
| RPP30 | ARL8A | SF3B3 | MACROD1 | EFTUD2 |
| REEP5 | ATXN2L | XRCC6 | SCO2 | NOP56 |
| SP100 | HLA-B | KRT18 | COX7A2 | HNRNPA0 |
| OXSR1 | CBX4 | HNRNPA1 | PCNP | ZFR |
| DHX38 | FLOT2 | XRCC5 | BMI1 | CPSF1 |
| LYPLAL1 | EIF4G2 | LMNB1 | TAF4 | MYO1C |
| VAPB | MBD3 | MAT2A | MRPS2 | PES1 |
| ATP6V1G1 | PSMD4 | HNRNPH1 | BTF3 | RPS13 |
| CDK12 | MRPS12 | HNRNPUL2 | PSMD4 | DHX15 |
| EXOC7 | ITPR1 | HLTF | MPG | YLPM1 |
| RPS3A | GTF2B | ANXA2 | TFAP2D | RBM12B |
| ACTB | UBE2D3 | GAPDH | DDX6 | CTNNA1 |
| TOP1 | NDUFB9 | NCL | POLRMT | SRSF9 |
| KRT1 | RFX3 | YLPM1 | CAPG | RPL10A |
| HNRNPC | IWS1 | HSPA8 | ALDH1A1 | ELAVL1 |
| PARP1 | INTS4 | HNRNPR | SUPT6H | KRT6A |
| H1-3 | CCDC51 | LMNB2 | TRIM41 | HNRNPA3 |
| H4C1 | CSNK1A1 | DHX9 | FOXC1 | FUBP3 |
| KRT9 | PIN4 | HNRNPL | EIF4E2 | MCM7 |
| HMGA1 | GOT1 | HNRNPA3 | ANKS1B | DDX17 |
| HNRNPU | ZFX | H2BC12L | BBX | RPS25 |
| KRT10 | TAF6L | NUP98 | USP5 | ALYREF |
| LMNA | SUPT20H | FLNA | SMPDL3B | SRRM2 |
| HNRNPK | MAD1L1 | PKM | ACAT2 | HNRNPA1 |
| HNRNPA2B1 | CCNB1 | SNRNP200 | SLC25A12 | NONO |
| KHDRBS1 | LCOR | DDX3X | ACP1 | KRT16 |
| RBM14 | PRC1 | DDX5 | SRP9 | RPS16 |
| PRKDC | ACTB | DDX21 | CSDE1 | SNRPD2 |
| KRT8 | MKI67 | H1-0 | SGF29 | SMARCA5 |
| HNRNPM | TOP1 | AHNAK | UTP4 | SUGP2 |
| NPM1 | KRT1 | KHSRP | PDHA1 | SRSF3 |
| RBM12B | HNRNPC | ELAVL1 | RBM22 | RAN |
| MATR3 | KRT10 | RPS18 | STAU1 | FASN |
| MKI67 | H1-3 | RPA1 | SRM | RPS9 |
| KRT2 | PARP1 | ILF2 | ATAD3B | NUP205 |
| ACTC1 | H4C1 | ACTN4 | OGT | XRCC6 |
| ANXA2 | KRT9 | EZR | RAI1 | SRSF10 |
| NCL | HNRNPU | ENO1 | TNPO3 | RBMX |
| HNRNPA1 | ACTBL2 | NONO | DHX38 | TMPO |
| HNRNPF | HMGA1 | HP1BP3 | BRMS1 | RSL1D1 |
| SSBP1 | KHDRBS1 | RPS3 | RTRAF | EXOSC10 |
| ILF3 | PRKDC | NOP56 | TASOR | FIP1L1 |
| HSPA8 | KRT8 | KRT5 | MRPL12 | RRP1B |
| ALB | HNRNPK | RBM17 | SFMBT1 | TUBB |
| GAPDH | HNRNPA2B1 | TOP2B | CHD8 | HSPA8 |
| HNRNPD | CSE1L | KRT14 | FNBP4 | RALY |
| KRT18 | KRT2 | SRSF1 | MOB1B | DDX24 |
| HNRNPH3 | LMNA | DBT | RNASE7 | SF3A1 |
| LMNB1 | RBM14 | SUB1 | KRT1 | RPLP0 |
| AHNAK | ACTA1 | ALYREF | KRT10 | RPL27 |
| HNRNPH1 | RBM12B | SMARCA5 | KRT9 | FLNA |
| HNRNPUL2 | HNRNPM | TOP2A | HNRNPC | RPL35A |
| RFC4 | MATR3 | RALY | KRT2 | RUVBL2 |
| HNRNPL | ALB | HSPD1 | PARP1 | PARP1 |
| KRT19 | ANXA2 | PRPF8 | TOP1 | SLC25A5 |
| LMNB2 | ILF3 | U2AF2 | H4C1 | RPL11 |
| FLNA | HNRNPD | NAT10 | H1-3 | RPS11 |
| YLPM1 | KRT19 | H2AZ2 | HNRNPU | RBM10 |
| XRCC5 | NCL | THRAP3 | HNRNPK | RPL13 |
| HNRNPR | HNRNPH3 | FASN | HNRNPA2B1 | IK |
| XRCC6 | HNRNPA1 | ATP5F1A | KRT8 | XRCC5 |
| EZR | SSBP1 | NUMA1 | LMNA | SMARCA4 |
| PKM | HNRNPF | NOP58 | KRT5 | SART1 |
| HNRNPA3 | GAPDH | FUBP3 | H2BC12L | RPS6 |
| SNRNP200 | NPM1 | MCM7 | CLTC | EEF1A1 |
| SF3B3 | XRCC6 | LIG3 | ACTA1 | PRPF19 |
| HNRNPA0 | HNRNPH1 | HMGB2 | HMGA1 | DSG2 |
| ACTN4 | XRCC5 | UBTF | ORC5 | RPL17 |
| MAT2A | LMNB1 | PLEC | HNRNPM | GTF2I |
| CCT4 | HSPA8 | ATP5F1B | NPM1 | RPS23 |
| EEF1A1 | HNRNPL | RAN | RBM14 | ALDH18A1 |
| NONO | DHX9 | MDC1 | KHDRBS1 | RPL23 |
| SYNCRIP | LMNB2 | HNRNPDL | MATR3 | CTNNB1 |
| DSP | ORC5 | RUVBL2 | ANXA2 | TUFM |
| ENO1 | MAT2A | RPS16 | HNRNPA1 | SLC25A1 |
| HSPD1 | CPSF2 | DSP | INTS1 | LYAR |
| U2SURP | CLTC | DHX15 | HNRNPD | MYO1B |
| CPSF2 | HNRNPR | PRPF19 | DSP | KRR1 |
| H3-3A | EZR | VDAC1 | KRT18 | HSPD1 |
| DDX5 | SYNCRIP | RPS4X | GAPDH | PCF11 |
| ILF2 | HNRNPA3 | PRPF6 | NCL | RPL30 |
| SFPQ | AHNAK | CTNNB1 | KRT19 | ZNF638 |
| FASN | PKM | HNRNPH2 | KRT16 | RPL23A |
| HSP90AB1 | PLEC | SLC25A5 | SF3B3 | RPS2 |
| ELAVL1 | SF3B3 | SRSF9 | RBM6 | PDS5A |
| RBMX | H3-3A | TRIP12 | ALB | WDR33 |
| DDX21 | NONO | TMPO | HSPA8 | MYH10 |
| ALYREF | ILF2 | TUBB | MKI67 | DDX23 |
| PRPF8 | HNRNPA0 | SNX9 | AHNAK | TRIP12 |
| RPS18 | FLNA | RBMX | MAT2A | RBM25 |
| KHSRP | ENO1 | RPS13 | HNRNPH3 | DDX18 |
| ATP5F1A | ACTN4 | KRT77 | SSBP1 | DDX27 |
| SRSF1 | SNRNP200 | SFPQ | HNRNPL | UBA52 |
| CBX3 | PDCD11 | FBL | PKM | SNRNP40 |
| RPS4X | RPS3 | KRT16 | LMNB1 | THRAP3 |
| SF3B1 | ATP5F1A | CNN2 | SYNCRIP | CHERP |
| DDX17 | SFPQ | PHB2 | HNRNPA0 | EZR |
| ATP5F1B | HLTF | EEF1A1 | HNRNPH1 | CHD4 |
| RPS3 | DDX5 | EFTUD2 | ENO1 | CPSF7 |
| SF3B2 | FASN | HNRNPAB | ACTN4 | MSH6 |
| PES1 | KHSRP | JUP | FLNA | HRNR |
| NOP56 | HSPD1 | SRSF10 | JUP | H2BC18 |
| TJP1 | DDX3X | DNAJC9 | HNRNPR | UQCRC2 |
| SMARCA5 | ELAVL1 | MYBBP1A | SNRNP200 | GTF3C1 |
| FSCN1 | CBX3 | AHCTF1 | ILF2 | NUP98 |
| MYH9 | FUBP3 | UQCRC2 | HNRNPA3 | VDAC2 |
| RALY | NUP98 | HADHA | PRSS1 | TRIM4 |
| KRT5 | DBT | RPL7A | LMNB2 | PTBP1 |
| HMGB2 | RPS18 | FSCN1 | MYH9 | DDX54 |
| UBE2S | DDX21 | CPSF1 | XRCC5 | ACTN4 |
| HSP90AA1 | PRPF8 | AKAP8 | KHSRP | ADAR |
| RPA1 | H2BC12L | H2BC21 | U2SURP | SF3A3 |
| RAN | PHB2 | ALDOA | XRCC6 | ATP5F1A |
| RUVBL2 | EEF1A1 | SRSF3 | HNRNPF | RPL18A |
| EFTUD2 | RPA1 | GTPBP4 | KRT6A | SNRPD1 |
| FLNB | HSP90AB1 | TUFM | PLEC | RFC2 |
| PLEC | FBL | RPS25 | RPS6 | CTTN |
| PHB2 | ALYREF | NUP93 | PRPF8 | TRIOBP |
| ALDOA | TOP2B | RPS9 | TPR | RPL14 |
| RBM17 | KRT5 | RPL11 | HSPD1 | HNRNPUL1 |
| FUBP3 | U2AF2 | TJP1 | TMEM33 | HNRNPH2 |
| HSPA1B | DSP | PTBP1 | ATP5F1A | LIMA1 |
| DHX15 | MYH9 | CHD4 | DDX21 | SFPQ |
| PRPF6 | RBM17 | RPS14 | HNRNPUL2 | NCL |
| TUBB | DDX17 | CHERP | YLPM1 | ATP5F1B |
| NOLC1 | HMGB2 | RUVBL1 | NONO | SNRPA1 |
| SUB1 | H2AZ2 | FLNB | DDX5 | CPSF6 |
| RPS14 | RALY | PDS5A | NUP98 | CDH1 |
| NAT10 | TOP2A | RPRD2 | HSP90AB1 | NUP155 |
| U2AF2 | FLNB | GNB1 | H3-3A | PRPF3 |
| TOP2A | NOP56 | RFC5 | RPS4X | KPNA2 |
| TMPO | FSCN1 | NOP2 | FASN | RPL3 |
| NOP58 | MCM7 | ALDH18A1 | ELAVL1 | SRSF6 |
| THRAP3 | NAT10 | HSP90AA1 | WIZ | HNRNPD |
| EEF2 | HSP90AA1 | SRSF7 | CTNNA1 | EHD4 |
| CNTNAP4 | NUMA1 | SMC1A | SRSF1 | HNRNPDL |
| SF3A1 | MDC1 | KRT6A | RPS18 | PNN |
| PTBP1 | RPS16 | H3-7 | SNRPD3 | AHCTF1 |
| SLC25A5 | RAN | RPL23A | EEF1A1 | BRIX1 |
| VDAC1 | ALDH18A1 | RPL8 | NUMA1 | INTS1 |
| FBL | PRPF6 | HMGB3 | FUBP3 | ANXA2 |
| TRIM28 | SLC25A5 | EIF4A3 | ATP5F1B | TIAL1 |
| MCM7 | CTNNB1 | RPL23 | PDCD11 | SMARCD2 |
| ORC5 | SRSF1 | GTF2I | SF3B1 | FDXR |
| PSIP1 | HMGB1 | RPL13 | RPS3 | RBM28 |
| H2AZ2 | RPS3A | SAFB | FLNB | NOC2L |
| TRIM4 | RPS14 | BAZ1A | ALYREF | EIF4A3 |
| GTF2I | JUP | HNRNPUL1 | PHB2 | ANAPC7 |
| ACTBL2 | EEF2 | RFC4 | DDX3X | CTNND1 |
| SAFB | ALDOA | EEF2 | ALDOA | RPL12 |
| RPS16 | VDAC1 | KIF22 | HMGB2 | DIDO1 |
| CHD4 | SF3B2 | DIDO1 | HMGB1 | GNB4 |
| SRSF10 | DHX15 | RPS11 | VDAC2 | XRN2 |
| TRIP12 | HNRNPDL | RPS23 | HSP90AA1 | TBL3 |
| VDAC2 | HSPA1B | PPIB | DDX17 | RPL5 |
| UQCRC2 | KRT14 | UBA52 | DHX15 | GAR1 |
| KRT77 | THRAP3 | USP39 | FSCN1 | WDR36 |
| GNB1 | VDAC2 | ZNF326 | SUB1 | ZNF326 |
| JUP | EFTUD2 | OAT | THRAP3 | RFC1 |
| SNX9 | SUB1 | PHB1 | UBA52 | RPN1 |
| RPL13 | FUS | RPL4 | EEF2 | TOP2B |
| NUP93 | TRIP12 | PPP1CA | EFTUD2 | PPP1CA |
| SPTAN1 | KRT77 | ADAR | U2AF2 | KPNB1 |
| DBT | HNRNPH2 | TRA2B | RALY | ACTL6A |
| ANXA6 | ACACA | SF3A1 | ALDH18A1 | CDK1 |
| PPIB | SF3A1 | TRIM28 | FBL | RPL24 |
| SMC1A | RUVBL2 | RPL6 | RBM17 | KRI1 |
| PHB1 | TUFM | NOLC1 | ACTBL2 | TFIP11 |
| EIF4A3 | PTBP1 | RPL27 | MDC1 | SMARCC1 |
| SNRPD2 | ANXA6 | DDX52 | HLTF | SRSF7 |
| AHCTF1 | KRT16 | RBM10 | NOLC1 | RPL6 |
| LDHB | UQCRC2 | SNRPD2 | SLC25A5 | RFC3 |
| SUPT16H | SNX9 | CALU | ING4 | PKP3 |
| SLC25A3 | TUBB | RPLP0 | CHD4 | RPL18 |
| SRSF3 | TRIM28 | SUPT16H | TRIM4 | NOC3L |
| HNRNPH2 | NOP58 | SNRPD1 | PHB1 | GNL3 |
| AP2M1 | RPL29 | APOBEC3C | TADA2B | RRP12 |
| SRSF7 | MYBBP1A | FIP1L1 | SMARCA5 | NDUFA10 |
| PDS5A | SRSF9 | TPR | NOP58 | PWP2 |
| TFAM | GNB1 | RPS8 | MYO1C | NUP107 |
| RPS25 | RPL7A | RFC1 | HNRNPDL | SPEN |
| RPS9 | RPS25 | RPS6 | HSPA1B | SRSF5 |
| TUFM | RPS13 | RBM6 | PRPF6 | HELLS |
| SRRM2 | TMPO | HSPA9 | ANXA6 | RANGAP1 |
| RPL8 | EIF4A3 | FBLL1 | RPS3A | RRP1 |
| TCOF1 | RPS9 | LDHB | RPS16 | NUP133 |
| HMGB1 | NUP93 | SMARCA4 | SUPT16H | DDX52 |
| DIDO1 | SNRPD2 | RPL10A | FUS | TOP2A |
| AKAP8 | RPL23A | SLC25A1 | DSG1 | SNRPB |
| RPS19 | SPTAN1 | PSIP1 | SRSF9 | KRT77 |
| LIG3 | SLTM | IK | RUVBL2 | KRT74 |
| SNRPB | RPL8 | RPL24 | RPL8 | SMARCC2 |
| HADHA | RPL11 | RSL1D1 | TRIM28 | SNRPD3 |
| RPS15A | SRSF3 | SNRPA1 | TUFM | RPS19 |
| HNRNPDL | SMC3 | ZFR | YARS1 | MSH2 |
| RPL29 | RPS19 | CPSF6 | TOP2B | RPF2 |
| GTPBP4 | LIG3 | HSP90B1 | SPTBN1 | DHX30 |
| CPSF1 | CHD2 | MSI2 | RPS14 | EMD |
| IMMT | RPL26 | NUP205 | CALML5 | RBFOX2 |
| RUVBL1 | HMGA2 | FDXR | MYBBP1A | RRS1 |
| RPL11 | LDHB | H3-3A | SRSF10 | GCN1 |
| RPL4 | RPL27 | SART1 | NAT10 | PGAM5 |
| TAF6L | CHD4 | ANXA6 | UQCRC2 | HSPB1 |
| SPTBN1 | HNRNPAB | WDR36 | CSTA | NUP153 |
| RPL23A | RUVBL1 | TBL3 | TMPO | HTATSF1 |
| HSPA9 | PPIB | MACROH2A1 | PTBP1 | RAB18 |
| TPR | CPSF1 | SRSF2 | RPA1 | CLTC |
| LRPPRC | PDS5A | RPL5 | LIG3 | MTREX |
| BAZ1A | GTPBP4 | SF1 | VDAC1 | PPP2R1A |
| RSL1D1 | HSPA5 | RANBP2 | MCM7 | PFKP |
| RPL23 | PSIP1 | RPS19 | LDHB | SMC1A |
| RPL26 | LDHA | KPNA2 | HNRNPAB | TOMM40 |
| RPS2 | NOP2 | KIFC1 | SRRM2 | DCD |
| CHERP | SPEN | RFC3 | SPTAN1 | RPL22 |
| HNRNPAB | KIF22 | MSH6 | SRSF3 | UBTF |
| SEPTIN9 | SART1 | ATP1A1 | RPS2 | RPS17 |
| ACACA | RFC5 | MIDEAS | RPS9 | NDUFS2 |
| RPL6 | H2BC21 | CTNNA1 | RPL27 | DBT |
| RFC5 | DIDO1 | SEPTIN9 | PPIB | RPL4 |
| SNRPA1 | AP2M1 | PUF60 | TKT | CDH3 |
| RPS23 | RANBP2 | RCN1 | ZFR | KRT15 |
| YWHAZ | AKAP8 | ACIN1 | RPS25 | SAMD1 |
| ACIN1 | DSG2 | RPS2 | TUBB4B | GNAS |
| UBA52 | RPS2 | DDX23 | SMC1A | DBN1 |
| SSRP1 | SAFB | MYO1C | CTNNB1 | AHNAK |
| GTF3C1 | ADAR | HRNR | SLC25A3 | PCBP2 |
| RPL27A | ZFR | SNRPB | PRPF19 | NHP2 |
| HSPA5 | RSL1D1 | YWHAZ | ADAR | DDOST |
| CPSF6 | SRSF10 | RPS15A | HSPA9 | RPL35 |
| SNRPD1 | UBA52 | DKC1 | ACACA | RPSA |
| CKB | RPL35A | HMGB1 | NUP93 | MAP4K4 |
| KIFC1 | SNRPB | SNRPD3 | LDHA | NUP160 |
| RPS11 | SLC25A3 | RPL12 | S100A7 | UBAP2L |
| NOP2 | RFC4 | NDUFA10 | SAFB | UQCRC1 |
| BCLAF1 | BAZ1A | HSPA5 | HADHA | PCBP1 |
| RBM10 | CTNNA1 | EHD4 | NOP2 | SNX9 |
| H2BC21 | RPS15A | SLC25A3 | RPL17 | MYL12B |
| KRT16 | RPL17 | PKP3 | PRDX1 | FTSJ3 |
| SMARCA4 | HSP90B1 | TADA2B | SSRP1 | PPHLN1 |
| RPLP0 | CCT3 | RRP1B | GTPBP4 | NIP7 |
| RPL27 | ZNF326 | TCF7L2 | RPL13 | SF1 |
| CCT8 | TKT | KDM1A | CPSF1 | RPL28 |
| CCT3 | HNRNPUL1 | PC | RUVBL1 | WDR18 |
| ZFR | PPP1CA | RUNX1 | RPS19 | KRT6B |
| TUBB4B | RPS23 | RPN1 | RSL1D1 | NFIC |
| EXOSC10 | SSRP1 | RPL27A | H2AZ2 | MYO1D |
| RPL17 | UHRF1 | NUP155 | RPL23A | RAD18 |
| RBM25 | ACTR3 | CTTN | RPL26 | MAGEB2 |
| RANBP2 | UBTF | CPSF7 | HSPA5 | H2BC21 |
| DDX23 | HSPA9 | SPEN | TUBB | PPP1CC |
| RPN1 | SNRPD1 | PPIA | PPIA | FSCN1 |
| SPEN | RPA2 | SF3A3 | RPL4 | WDR3 |
| RPS6 | RPRD2 | H1-10 | RBMX | ENO1 |
| RPS8 | RPL10A | GTF3C1 | RPL10A | SUPT5H |
| HMGA2 | SRSF7 | UQCRC1 | RPS11 | HEATR1 |
| RPL10A | TCP1 | SMC3 | HSPB1 | UTP3 |
| CPOX | UQCRC1 | CCT8 | HSP90B1 | SSBP1 |
| SLC25A1 | RBM10 | MSH2 | PDS5A | POLDIP3 |
| RFC2 | SLC25A1 | RPL31 | KRT6B | PUF60 |
| RPA2 | SPTBN1 | NUP210 | RPL6 | NOM1 |
| NUP210 | TPR | SSRP1 | RPL11 | CSE1L |
| MYO1C | AHDC1 | TCP1 | PUF60 | RPS7 |
| WIZ | KRT79 | TKT | MYH10 | KHDRBS3 |
| HSP90B1 | CPOX | CASZ1 | LRPPRC | RBM15 |
| H3-7 | RPL6 | CDK1 | SRSF7 | MSI2 |
| DSG2 | FDXR | CPOX | KRT17 | RPL7 |
| IK | RPL5 | RBM4 | UBTF | NKRF |
| SMC3 | RPN1 | NFIC | RPLP0 | UTP18 |
| ACTR3 | CPSF6 | EEF1G | DSG2 | GTF3C2 |
| UHRF1 | CDC5L | PYCR1 | TOP2A | ATAD3A |
| SNRNP70 | NUP205 | IMMT | SART1 | SNRPE |
| RPS20 | CCT8 | RPL3 | RPS23 | TGM3 |
| SF1 | RPS6 | RB1 | RPS8 | DDX51 |
| CTNND1 | RPS11 | PGAM5 | PSIP1 | RPL34 |
| SRSF2 | KRT6A | MECP2 | SNRPA1 | MYO6 |
| TCP1 | OAT | SLC2A1 | CHERP | GTF3C4 |
| PPP1CA | RPL21 | RREB1 | DIDO1 | KRT78 |
| ZNF638 | SRRM2 | DSG2 | RPN1 | DYNC1H1 |
| PGK1 | CTTN | ACTL6A | RPL12 | NUP210 |
| DKC1 | RBMX | RPS20 | HNRNPH2 | SDAD1 |
| SNRNP40 | PUF60 | PCBP2 | CPOX | ZMYM4 |
| ATP1A1 | H3-7 | LRPPRC | RPL21 | ACIN1 |
| PPIA | RPL27A | TARDBP | FLG | INTS6 |
| FBLL1 | RFC3 | TOMM20 | YWHAZ | HSPA1B |
| SMU1 | MSH6 | EMD | UQCRC1 | URB1 |
| RREB1 | LRPPRC | SAMD1 | RPL23 | ITPRID2 |
| FDXR | RPL31 | KRT6B | RPL27A | RAI14 |
| CNN2 | RPS8 | CDH1 | BAZ1A | CSTF3 |
| NFAT5 | FIP1L1 | RPL22 | SLC25A1 | RBM5 |
| PUF60 | IMMT | RPSA | CCT8 | POP1 |
| RFC1 | HMGB3 | RPL35A | SEPTIN9 | EIF4A1 |
| PNN | EXOSC10 | SRSF6 | KRT79 | DHX37 |
| RACK1 | RPL14 | PCBP1 | RPS15A | PRPF4 |
| HMGB3 | FUBP1 | CCT6A | DDOST | RPL9 |
| PFN1 | PES1 | TMPO | SNX9 | EEF1D |
| CHD2 | TRA2B | SPTBN1 | CCT3 | TRA2B |
| IQGAP1 | NCOA5 | DDB1 | CCT6A | HMGB1 |
| HSPB1 | SEPTIN9 | CPSF2 | USP39 | RBBP4 |
| RPL7 | PDHB | SNRNP40 | FABP5 | DOCK7 |
| CDK1 | GTF3C1 | DDOST | AHCTF1 | ATAD3B |
| ZNF326 | YWHAZ | RPL13A | PFN1 | ACOT9 |
| FUBP1 | SNRPD3 | CCT3 | CHD2 | RPL10 |
| KPNB1 | CNN2 | PGK1 | GTF3C1 | XPO1 |
| PRSS1 | RPL12 | ZNF638 | ACTN1 | POLR2B |
| PRDX1 | RBM6 | RPL7 | CPSF6 | GTF3C5 |
| DDB1 | WDR36 | KRT74 | DDB1 | RBM4 |
| TPI1 | KPNB1 | CTNND1 | MTA1 | PPP1R9B |
| RCC1 | SRSF2 | PSMB1 | RPL31 | VARS1 |
| CCT7 | MYO1B | SMARCC1 | SOD1 | GTF3C3 |
| KRT6A | DDX41 | HSPB1 | SMARCA4 | SMC3 |
| ACTN1 | DDX23 | BAZ2A | DKC1 | MISP |
| RRP1B | SRSF6 | LRRC59 | IMMT | PELP1 |
| LYAR | DKC1 | PNN | MDH2 | RBM27 |
| ACTL6A | SERBP1 | SYMPK | RFC4 | CFAP20 |
| PDIA3 | SMARCA4 | PFKP | RPL5 | CNN3 |
| MT-CO2 | BUB3 | RPL18A | PCBP2 | RPS20 |
| VDAC3 | ANXA5 | RCC2 | H2BC20P | SUPT16H |
| RBM6 | NDUFA10 | RPL21 | COX4I1 | ALDH1A3 |
| P4HB | SLC25A10 | RPL9 | RPL3 | RPS5 |
| DBN1 | CCT6A | KPNB1 | RPL35A | SUN2 |
| RBM39 | MSI2 | NDUFS2 | RPL7 | ARGLU1 |
| PKP3 | P4HB | TPM3 | RPSA | HDAC1 |
| PCBP1 | RPS5 | TOP3A | RANBP2 | IMPDH2 |
| FIP1L1 | RPL3 | XRN2 | CDC5L | TRIM28 |
| TBL3 | RCC1 | PDLIM5 | RFC5 | ZC3H18 |
| U2AF1 | PGK1 | MTA2 | TIAL1 | EEF1G |
| WDR36 | EHD4 | PPP1R10 | GGCT | PPP1R10 |
| CASZ1 | TMPO | LYAR | TFAM | TUBG1 |
| TIAL1 | HRNR | H2AC21 | PDIA3 | MAGOH |
| CPSF7 | RFC2 | RAE1 | CCT2 | L3MBTL3 |
| ANXA1 | RRP1B | ATP5PO | P4HB | MDN1 |
| RBBP4 | ACTN1 | RBM39 | RRP1B | RPS15 |
| ANXA5 | CAND1 | TOMM40 | ZNF638 | LCN1 |
| DDX27 | PRDX1 | RPL35 | LYAR | GNL2 |
| DEK | CPSF7 | GNL3 | MYO1B | CPSF3 |
| PDLIM5 | RACK1 | BCLAF1 | FIP1L1 | BAZ1A |
| AHCY | BCLAF1 | PPP2R1A | TPI1 | BOP1 |
| EIF4A1 | DDB1 | ACTN1 | H3-7 | BAZ1B |
| SLC2A1 | PCBP1 | P4HB | EEF1G | NT5DC2 |
| MSN | MACROH2A1 | KPNA4 | PDHB | RBM26 |
| CTTN | INTS1 | NUP85 | SLC2A1 | CCDC47 |
| PCBP2 | P4HA1 | TFIP11 | RPL13A | SF3A2 |
| PPP2R1A | MT-CO2 | TUBB4B | TCOF1 | RPS10 |
| TRRAP | ZNF638 | NME1 | DOCK6 | DDX41 |
| SRSF6 | CDK1 | DBN1 | DDX27 | PPAN |
| MIDEAS | PRPF40A | HELLS | IQGAP1 | NOP9 |
| TARDBP | BAZ2A | MCM4 | KRT78 | LRRC59 |
| RPS10 | PDIA3 | EBNA1BP2 | RBM10 | PHB2 |
| SARNP | CCT4 | SMU1 | WDR36 | ARF3 |
| P4HA1 | SF1 | CCT4 | NUP210 | HLTF |
| RB1 | SF3A3 | RBBP4 | SNRNP70 | YBX1 |
| MCM4 | IQGAP1 | PRPF3 | SARNP | CHD2 |
| RPL13A | PGAM5 | ANAPC7 | CCT4 | RPL29 |
| RPL3 | RPSA | RPL38 | MYO1D | KRT17 |
| NFIC | ACLY | CBX3 | SMU1 | SLC25A11 |
| DDOST | SMC1A | SRRM1 | NUP155 | CPSF4 |
| CCT2 | LRRC59 | RPS10 | RFC2 | PPIB |
| NDUFA10 | CALU | SARNP | KDM1A | DDX55 |
| DYNC1H1 | EIF4A1 | CHD7 | KIFC1 | FLG |
| RAP1B | CASZ1 | PPP1CC | BUB3 | TRRAP |
| SAMD1 | RPL10 | ATAD3A | RPL18A | MDC1 |
| RPL31 | TBL3 | RACK1 | YWHAE | PPP1CB |
| DNM2 | DEK | WIZ | DDX52 | UACA |
| BAZ2A | TARDBP | WDR3 | FUBP1 | SRP14 |
| MTA2 | TJP1 | PRDX1 | RPA2 | DDX50 |
| HNRNPUL1 | GCN1 | MYO1B | SMC3 | DDX31 |
| UBAP2L | PDLIM5 | RRP12 | KPNB1 | ADNP |
| GCN1 | SYMPK | INTS1 | NDUFS2 | MT-CO2 |
| PPP1R10 | RALYL | MAGEB2 | RBBP4 | NOL6 |
| YWHAE | PPP2R1A | DNM2 | ANXA1 | NDUFS3 |
| WDR3 | RPL18A | NOC2L | CKB | MCM3 |
| CDC5L | COX4I1 | NUP160 | RCC2 | GNAI2 |
| CAND1 | RBM39 | CKB | ZNF326 | TMPO |
| CALU | RPL7 | NCKAP1 | EEF1D | PFKL |
| PA2G4 | PFN1 | JUNB | ARG1 | DDX47 |
| PYCR1 | TEAD1 | GTF3C2 | GAR1 | CCDC50 |
| SMARCC1 | PABPC1 | EEF1D | TMPO | CRNKL1 |
| GANAB | APEX1 | SNRNP70 | PNN | RAVER1 |
| POLR1E | XPC | PABPC1 | IK | SEC11A |
| MACROH2A1 | NDUFS2 | BUB3 | DBN1 | RPL19 |
| ST13 | KHDRBS3 | BAZ1B | MSI2 | LEMD3 |
| KDM1A | LYAR | PRMT1 | RPL30 | RPN2 |
| PPHLN1 | CCT2 | RPL10 | PABPC1 | CDC16 |
| RPL21 | H1-10 | MRPS26 | PGAM5 | RPL22L1 |
| KIF4A | CDH1 | PDCD6 | PRPF40A | RPP30 |
| SNRPE | RB1 | BLM | DEK | SNW1 |
| RBM15 | CBX8 | KRR1 | RPS20 | TNPO1 |
| EHD4 | RUNX1 | DNTTIP1 | BCLAF1 | RBM8A |
| LIN7C | TOMM40 | CCT2 | CDK1 | PALLD |
| EMD | ANXA1 | RCC1 | EHD4 | TEX10 |
| PABPC1 | MECP2 | HDAC1 | SNRPE | EEF2 |
| ANAPC7 | WDR33 | POLR2B | PKP3 | RPA2 |
| NUP155 | SNRNP40 | SSR1 | CDH1 | SFSWAP |
| CDH1 | CCT7 | NDUFV1 | TUBB2A | POLR2A |
| PRPF3 | GRHL2 | API5 | PSMA5 | RPS27 |
| NDUFS1 | ST13 | GNA12 | DSC1 | NUP43 |
| APOBEC3C | NDUFS1 | CYCS | RPS10 | ATP1A1 |
| SYMPK | RREB1 | CAND1 | HNRNPUL1 | YTHDC1 |
| SMARCC2 | PYCR1 | RAD23B | RPL24 | WDR82 |
| KHDRBS3 | RBBP4 | SON | EXOSC10 | ZCCHC9 |
| CBX8 | IK | XPO1 | ACTL6A | NXF1 |
| DDX42 | SARNP | DDX46 | PYCR1 | YES1 |
| MSI2 | EMD | SMARCD2 | CTNND1 | BUB3 |
| RPL14 | HELLS | SMARCC2 | MRPL37 | ATP5PO |
| XPC | JUNB | YWHAE | TBL3 | PPFIBP1 |
| RPL10 | EEF1D | RPL34 | EIF4A1 | FLNB |
| SSR1 | SMARCD2 | MTA1 | TGM3 | DSG1 |
| PC | POLR1E | PWP2 | MSH6 | SRSF8 |
| XRN2 | ITPRID2 | CSE1L | ST13 | TADA2B |
| EEF1D | KRT13 | XPNPEP3 | AKAP8 | AKAP8 |
| NIPBL | IPO5 | CCT7 | CBX3 | CBX3 |
| LIMA1 | GAR1 | TFAP2A | WDR33 | DDX10 |
| HCFC1 | PA2G4 | RPL26 | SF1 | NCKAP1 |
| MRPL37 | ATP5PO | FEN1 | RACK1 | PLEKHA5 |
| RCN1 | RPS20 | TAF15 | PCBP1 | DIMT1 |
| JUNB | MCM4 | DHX30 | TPM3 | CAPZB |
| SRBD1 | SNRPE | CAPN1 | APEX1 | PAXBP1 |
| HSD17B4 | RCN1 | NUP107 | PA2G4 | HSP90AA1 |
| SRRT | AHCY | L3MBTL3 | KIF22 | RPL36 |
| MYO1B | RPL24 | BRIX1 | AHCY | SF3B6 |
| SURF6 | SMARCC1 | EHMT2 | CAND1 | RRBP1 |
| ZNF384 | CCT5 | RAD18 | NDUFA10 | DOCK6 |
| PRMT1 | WDR3 | POLDIP3 | CCT7 | CHTOP |
| PFKP | VARS1 | RBBP6 | DDX18 | DNTTIP2 |
| ATP5PO | SAMD1 | ANXA1 | RPL9 | SON |
| RCC2 | ING4 | POLR1E | NDUFS1 | POLR1G |
| RPL30 | TUBB2A | CYC1 | FDXR | NOC4L |
| XPNPEP3 | ACTL6A | MTDH | XRN2 | RSL24D1 |
| SNRPA | DDX42 | RBFOX2 | RBM15 | EIF6 |
| POLR1F | SMU1 | PRPF4 | MECP2 | RPS24 |
| RPL18A | MIDEAS | RAI14 | NME1 | SEPTIN9 |
| MDH2 | RPL9 | ARF4 | PSMA1 | MTX1 |
| LRRC59 | RRBP1 | NHP2 | SRSF6 | TPM3 |
| NOC2L | CKB | HMCES | ARF3 | SNU13 |
| TAF15 | NME1 | PPHLN1 | HELLS | SLC25A6 |
| ING4 | MRPL37 | RBM5 | NUP205 | CALML5 |
| SMARCD2 | ZNF384 | ZNF384 | HCFC1 | SENP3 |
| RUNX1 | RCC2 | ITPRID2 | EMD | RRP9 |
| RPL24 | ATAD3A | UBAP2L | KPNA2 | WAPL |
| IPO5 | PRMT1 | HSD17B4 | XPC | CYC1 |
| APEX1 | MAP4 | TERF2 | NME2 | FAF2 |
| CYCS | ANAPC7 | RPS26 | PRMT1 | U2AF1 |
| AFG3L2 | RBM4 | RPL22L1 | API5 | S100A9 |
| ATAD3A | RPL22L1 | NFATC2 | EIF5A | NUDT21 |
| ADNP | PPP1R10 | AKAP8L | HMGA2 | DDB1 |
| HSD17B10 | KRR1 | POLR1G | AP2B1 | POLR1E |
| SERPINH1 | TCOF1 | RPN2 | RPL19 | ALDH1B1 |
| INTS1 | RPL38 | DDX42 | DDX46 | DAP3 |
| NME2 | GANAB | PDIA3 | PEBP1 | TRMT1L |
| RPL9 | CHD7 | RPS5 | TJP1 | LUC7L2 |
| DDX49 | SNRPA | RANGAP1 | PPP1R10 | ARID1A |
| TOMM40 | PRPF3 | ZBTB20 | PRPF4 | MFAP1 |
| RRP12 | GNL3 | NUP133 | GNAS | SRRM1 |
| DDX54 | RBM5 | PPP1R9B | RRP12 | ESF1 |
| USP39 | PSMB1 | YTHDC1 | ANAPC7 | IGF2BP2 |
| EHMT2 | RPL13A | SEPTIN2 | ATP5F1C | CAPZA1 |
| NME1 | WIZ | SLTM | KRR1 | CNOT1 |
| DDX46 | APOBEC3C | ZNF24 | PPP2R1A | NQO1 |
| GAR1 | XRN2 | GTF3C4 | TCF7L2 | SERPINB12 |
| ARID1A | SERPINH1 | CDCP1 | CBX8 | SIN3A |
| EIF3A | SRRT | POGZ | DYNC1H1 | MRPS7 |
| RPL35 | SRP14 | GNAS | WDR3 | PMPCB |
| GPI | NOC2L | XRCC1 | EBNA1BP2 | MTA1 |
| MAGEB2 | DDX46 | YBX1 | TARDBP | ARF4 |
| RPSA | NOC3L | PSMC2 | PDIA4 | FUBP1 |
| NUP98 | TCERG1 | VCP | DDX41 | IMMT |
| SUPT5H | GNAS | ACOT9 | RPL18 | CSNK2A1 |
| CPNE3 | MAGEB2 | NDUFS3 | GANAB | CDK9 |
| RPL34 | MTA2 | PSMA7 | RBM39 | HSPA9 |
| TCF7L2 | PPP1CC | RPL28 | ATP5PO | CEP131 |
| MTDH | PWP2 | FOXK2 | MACROH2A1 | INTS2 |
| DNMT1 | VCP | DDX51 | XPO1 | TCOF1 |
| GLYR1 | LIMA1 | PPP1CB | DDX49 | INTS5 |
| MRPS7 | TOMM20 | DDB2 | NFIC | TENT4B |
| SF3A2 | NUP85 | BOP1 | TRRAP | RPF1 |
| SMARCE1 | H2AC21 | DDX54 | DNM2 | RAB35 |
| NOC3L | TPM3 | SFXN1 | CPNE3 | RAB13 |
| CORO1B | VDAC3 | GCN1 | RPL38 | AAAS |
| CCT5 | DHX30 | ATAD1 | SRBD1 | NOP16 |
| FUS | PPP1CB | TCF20 | NOC2L | EHMT2 |
| ITPRID2 | UGT2B17 | MTREX | DCD | TFCP2 |
| EBNA1BP2 | S100A4 | NUP153 | POGZ | MAD1L1 |
| RPS17 | CANX | DHX37 | RANGAP1 | RPA1 |
| CDCA7L | MTDH | RRP9 | RCN1 | TJP3 |
| BUB3 | POLR2B | SUPT5H | ALDH9A1 | FXR2 |
| RAE1 | PABPN1 | NACA | CASP14 | SPOUT1 |
| CAPN1 | PPM1G | KPNA3 | CANX | BAG2 |
| SEPTIN2 | PSPC1 | EPRS1 | SEPTIN2 | TCF7L2 |
| NUP133 | SMARCC2 | PMPCB | CORO1B | SEC61A1 |
| RPL28 | PRPF4 | CANX | ATAD3A | RBM7 |
| AKAP12 | NCKAP1 | MDH2 | H1-10 | NCBP1 |
| POLR1G | KDM1A | AHCY | MAGEB2 | ZNF768 |
| PDIA4 | HDAC1 | HTATSF1 | PWP2 | RPL7L1 |
| KRT17 | EBNA1BP2 | RPL29 | PDLIM5 | SMU1 |
| XPO1 | POLDIP3 | NME2 | LRRC59 | TMEM214 |
| PSPC1 | MSH2 | AAAS | POLR1E | PRPF31 |
| EIF2S3 | PDIA4 | SET | HADHB | DDX56 |
| H2AC21 | MTA1 | RBM27 | SRP14 | ACAT1 |
| NUP153 | DNM2 | RPS7 | NFAT5 | NSUN5 |
| KRR1 | HADHB | ATP5F1C | MIDEAS | PSPC1 |
| CANX | GEMIN5 | GNAI2 | RBM4 | SEPTIN7 |
| SLC25A11 | ADNP | CCDC47 | TOMM20 | FOXK2 |
| EIF5A | KPNA2 | G3BP1 | ANXA5 | PUM3 |
| GNAS | NUP153 | RPL18 | RTCB | MTDH |
| RBBP6 | CPNE3 | RBM8A | RPL15 | CYFIP1 |
| PPP1CC | TUBB4B | STIP1 | DDX54 | SMPD4 |
| MECP2 | KRT72 | PA2G4 | GNAI2 | CALU |
| MSH2 | MTREX | KIF4A | GTF3C2 | RCN1 |
| RBFOX2 | UQCRFS1 | MYL12B | NUP133 | NIPBL |
| RPL35A | TADA2B | IGF2BP2 | DDX24 | TOE1 |
| MTREX | USP39 | MRPS7 | RPL28 | WDR5 |
| TPM3 | SET | CDC27 | S100A8 | ZNF592 |
| ACOT9 | KRT17 | MYH14 | PPP1CC | CSTF1 |
| ATP5F1C | GTF3C4 | ABHD10 | PPP1CB | BUD13 |
| EIF2S1 | NUP107 | TCERG1 | HDAC1 | CDCP1 |
| MTHFD1 | BLM | TPI1 | HSD17B4 | RCC1 |
| CALR | SUPT5H | UTP18 | CCT5 | NELFCD |
| CSTF3 | BAZ1B | CSTF3 | RAB35 | PYCR1 |
| SFXN1 | KRT6B | ZBTB10 | MYL12B | MTCH2 |
| GTF3C2 | STOML2 | WDR82 | SEPTIN7 | DSC1 |
| CHD7 | BAG2 | TRIOBP | PES1 | CDCA7L |
| RBM5 | SEPTIN2 | SNRPA | HDGF | BMS1 |
| PLRG1 | NUP133 | CHAMP1 | SYMPK | HADHB |
| PPP1CB | DYNC1H1 | PHC2 | SAMM50 | TOMM22 |
| SRSF5 | CHCHD3 | COX6C | SNU13 | INTS13 |
| RRP9 | RRP12 | ALDOC | NDUFS3 | MIDEAS |
| CHCHD3 | EIF2S2 | DYNC1H1 | MSX1 | DPF2 |
| KRI1 | RAD18 | TPP1 | BRIX1 | RPS27L |
| WDR1 | HCFC1 | CDK11B | ACAT1 | ANAPC2 |
| UQCRFS1 | MSX1 | CCAR2 | RPL34 | CAPRIN1 |
| L3MBTL3 | TFIP11 | PLRG1 | MTREX | ARF6 |
| POLR2B | DDX52 | SRBD1 | POLR2B | PAK1IP1 |
| PEBP1 | GNAI2 | ARGLU1 | CHCHD3 | ERCC2 |
| MRPL38 | CCDC47 | FOXK1 | GNL3 | COX20 |
| SON | L3MBTL3 | MISP | EWSR1 | PHB1 |
| NUP160 | MYO1D | UHRF1 | SEPTIN11 | WDR12 |
| FOXK1 | WDR82 | GOT2 | STIP1 | MTA2 |
| BAG2 | CALR | ALDH1B1 | CCAR2 | RAD21 |
| NFATC2 | SMARCE1 | RPL15 | CDCP1 | CAND1 |
| RPN2 | UBAP2L | ARPC4 | ACLY | TERF2 |
| POGZ | IMPDH2 | TRRAP | LASP1 | IPO7 |
| ABHD10 | CORO1B | DAP3 | SLTM | KIF22 |
| EIF2S2 | CAPN1 | SMN1 | NUP160 | ASPH |
| DOCK7 | PLRG1 | APOBEC3B | RBM8A | CWC22 |
| NCOA5 | MAGOH | GANAB | SUPT5H | CP |
| TFIP11 | HSD17B4 | NOP9 | RBM3 | LLGL2 |
| VCP | KRI1 | CPNE3 | PDP1 | PHC2 |
| STT3A | KIF4A | RBM7 | NUP107 | EZH2 |
| LASP1 | SLC25A11 | INO80 | ALDH1A3 | HSPA5 |
| ALDOC | ZNF24 | SERPINH1 | SF3A2 | DNM2 |
| CORO1C | KIFC1 | RHOG | MTA2 | ERC1 |
| CS | FTSJ3 | ARID1A | NACA | FLG2 |
| DDX52 | NOL6 | CDK9 | NUP153 | MAK16 |
| SLC25A6 | SRBD1 | PLS3 | RPS17 | RNPS1 |
| SMARCB1 | CDCP1 | TEAD1 | KPRP | NMNAT1 |
| MYO1D | RPN2 | ACLY | FOXK1 | SCAF11 |
| TCERG1 | TOP3A | NIPSNAP1 | SRRT | INTS12 |
| POLR2A | LUC7L2 | LIMA1 | CALU | LZIC |
| MFAP1 | NACA | RPS17 | ZNF207 | CLP1 |
| FOXK2 | CYC1 | CTBP2 | KHDRBS3 | BYSL |
| NDUFS2 | NDUFS3 | CFL1 | RPN2 | MYO3B |
| ZC3H18 | RBFOX2 | CALR | RPL29 | STAU1 |
| FTSJ3 | ACOT9 | ASPH | FEN1 | CKAP4 |
| RPL19 | HADHA | SLC25A4 | SBSN | WRN |
| NACA | SMARCB1 | DDX27 | TP53BP1 | TOX4 |
| POLDIP3 | SHMT2 | CORO1B | ZNF384 | RAB5C |
| VRK1 | MCM3 | SEH1L | SRSF5 | NUP37 |
| SET | CSTF3 | PHF2 | NDUFV1 | PSMD2 |
| NHP2 | RPS27 | UQCRFS1 | UBAP2L | HIP1R |
| RPS26 | PDLIM7 | FUS | GPI | SSR4 |
| SMARCA1 | TCF20 | PHLDB2 | RBFOX2 | ESRP1 |
| CMAS | EWSR1 | VARS1 | ELMO3 | S100A7 |
| AP2B1 | KPNA3 | CTCF | NUDT21 | GIGYF2 |
| TEAD1 | ATP5F1C | MTHFD1 | NIPBL | NDUFA12 |
| KPNA3 | DNMT1 | RBM3 | POLDIP3 | INTS8 |
| EWSR1 | ARPC4 | GTF3C3 | STT3A | PTPN1 |
| SAMM50 | MSN | RTCB | ALDOC | SFXN1 |
| ARPC4 | RBBP6 | DNTTIP2 | CYC1 | WDR75 |
| HADHB | AKAP12 | NQO1 | SFXN1 | PSMD3 |
| CHTOP | SRSF5 | MAFG | NOC3L | MACROH2A1 |
| RPS5 | DDX24 | NUDT21 | PSMB5 | USP39 |
| GTF3C4 | TRRAP | SRSF5 | CHTOP | PABPC4 |
| CHD9 | DDX47 | WDR5 | CDH3 | ABLIM1 |
| TERF2 | STT3A | RBM4B | SERPINB12 | NDUFV1 |
| PBRM1 | LLGL2 | CFAP20 | LUC7L2 | SERPINB3 |
| SHMT2 | GTF3C2 | LASP1 | RRBP1 | BCLAF1 |
| SRP14 | MYH10 | RPL36 | UQCRFS1 | EWSR1 |
| XAB2 | SON | NDUFA13 | RBM5 | KPNA3 |
| HTATSF1 | RTCB | ARF6 | RAB5C | H1-0 |
| WDR82 | KRT78 | POLR2A | S100A9 | RPS19BP1 |
| EHMT1 | CORO1C | SMARCA1 | PRDX2 | SPCS2 |
| KIF2A | RPL34 | NDUFB4 | SERBP1 | RHOG |
| XRCC1 | NFATC2 | DRAP1 | CSTF3 | SPATA5L1 |
| RPL36 | RANGAP1 | EWSR1 | EIF2S2 | RCL1 |
| RNPS1 | POGZ | SCAF11 | RAD18 | DNAJA1 |
| DHX30 | VRK1 | PIP | PSMA7 | SNRPA |
| ARPC2 | ATP1A1 | MRPL38 | CTBP2 | RB1 |
| NDUFS3 | TRIOBP | INTS2 | CALR | NIFK |
| SEPTIN11 | DDX1 | NSUN5 | MTHFD1 | CAVIN1 |
| SEPTIN7 | ARF4 | SMAD4 | TCERG1 | XAB2 |
| RTCB | YBX1 | E2F3 | EIF2S3 | RAC1 |
| CSNK2A1 | HK1 | NSD3 | STOML2 | ORC5 |
| TCF20 | EIF2S3 | SDAD1 | CNN3 | PHLDB2 |
| CDCP1 | POLR1G | MCM5 | ACOT9 | WTAP |
| SRRM1 | ZC3H14 | SSR4 | PSMC2 | BBX |
| GNL3 | DDX54 | MTCH2 | CSE1L | TRMT10C |
| CDK11B | DDOST | TOX4 | NDUFA13 | FKBP8 |
| NQO1 | DNTTIP1 | TOR1AIP1 | PSPC1 | CASP14 |
| LEMD3 | DLD | LLGL2 | MCM4 | SRRT |
| COX6C | HMCES | VRK1 | RPRD2 | SRPRB |
| RANGAP1 | SF3A2 | TRMT10C | VARS1 | GPRC5A |
| TRA2A | ZBTB20 | ZBTB7A | PLS3 | ORC4 |
| PSMA7 | LASP1 | SLC25A22 | SERPINH1 | RBM34 |
| PHF2 | RAI14 | SRRT | ADNP | IMP4 |
| NUP85 | PGAM1 | NFIA | TEAD1 | PDLIM5 |
| BRIX1 | COX6C | RPL7L1 | RPL36 | APEX1 |
| CSE1L | RAP1B | GLYR1 | RB1 | AZGP1 |
| RPL37A | ACAT1 | NFIX | PKP1 | NUP35 |
| DDB2 | ATP5PB | CPSF3 | SLC25A6 | PIP |
| RBM8A | RRP1 | MAGOH | RPS5 | NOL11 |
| KPNA2 | BRIX1 | CBX5 | ATP1A1 | LDHB |
| IMPDH2 | CDK11B | NCBP1 | UHRF1 | ACLY |
| MCM3 | SSB | CAPRIN1 | CYB5B | SAP18 |
| TRIOBP | PMPCB | SEPTIN11 | DDX1 | ZBTB7A |
| INO80 | POLR2A | GTF3C5 | SUGP2 | TPP1 |
| ARF4 | RBM26 | ANXA3 | RPL22L1 | MRPL11 |
| MAGOH | ABHD10 | RBM26 | EHMT2 | SUZ12 |
| MTHFD2 | RPL32 | INTS6 | PGAM1 | KPNA1 |
| CTCF | BSG | ZNF148 | NUP85 | RING1 |
| CHAMP1 | DSG1 | AFG3L2 | HARS1 | KNOP1 |
| HMCES | CYCS | NUMB | TXN | ABCF3 |
| GTF3C3 | MYL12B | HEATR1 | NCKAP1 | NSA2 |
| PSMC2 | NME2 | DDX47 | YTHDC1 | JUNB |
| MAP4 | FLG | SUZ12 | PCNA | SARNP |
| ATP5PB | GNAI1 | TUBG1 | CDC27 | HSD17B4 |
| CKAP4 | MRPL38 | EIF2S2 | FLG2 | JUND |
| ZNF207 | MRPS7 | CDC73 | MTDH | RHOC |
| CDC73 | EHMT2 | GPI | L3MBTL3 | WDR74 |
| DSG1 | EIF2S1 | CNN3 | TRA2A | CPOX |
| PCNA | UBA1 | KRAS | CORO1C | KRAS |
| NDUFA13 | AAAS | DDX1 | MCM6 | NR2C2 |
| CFL1 | PHC2 | LY75 | BSG | VDAC3 |
| SLC25A4 | CAPRIN1 | CYB5B | CKAP4 | ZMYM3 |
| PACSIN2 | MYH14 | CCT5 | MSH2 | SLU7 |
| AKAP8L | TRA2A | SERBP1 | RPS26 | EHMT1 |
| RAB7A | STIP1 | ZCCHC9 | MSN | DCAF7 |
| PRDX6 | EEF1A2 | UQCR10 | CYB5R3 | SNRNP70 |
| NUDT21 | SFXN1 | WDR1 | UBA1 | SMC2 |
| CSTF1 | RPL37A | PPFIBP1 | DNMT1 | KIF23 |
| RBM26 | AKAP8L | CLP1 | RRS1 | OCIAD1 |
| UTP18 | NDUFA13 | PEBP1 | CAPN1 | RACGAP1 |
| RAI14 | PFKL | DNAJB1 | SMC2 | PFKM |
| RRP1 | NDUFV1 | ZIC2 | PMPCB | UQCR10 |
| ELAVL3 | DAP3 | SPCS2 | UTP18 | DDX46 |
| ZC3H14 | UTP18 | CHCHD3 | EPS8 | DHX16 |
| ZNF24 | YY1 | MYH10 | KRAS | ARG1 |
| ZC3H4 | NIPSNAP1 | RING1 | NDUFB10 | LDHA |
| RPF2 | WDR1 | RAVER1 | RPS27 | SLC25A4 |
| PSMD2 | PHLDB2 | WRN | H2AC21 | PAF1 |
| IGF2BP2 | SMARCA1 | ZC3H11A | SHMT2 | PRDX1 |
| TOR1AIP1 | PHF2 | TP53BP1 | OCIAD1 | MTHFD1 |
| S100A4 | TOX4 | GIGYF2 | CAVIN1 | UTP20 |
| DDX1 | CAV1 | CYFIP1 | SEH1L | CTBP2 |
| HDAC2 | PCNA | PBRM1 | WDR1 | GNAI1 |
| H1-10 | PLS3 | FAU | G3BP1 | DDX19B |
| NDUFB10 | DDX39B | CCDC86 | IMPDH2 | ATAD1 |
| NFATC1 | ALDH1A3 | IPO7 | AKAP12 | LENG8 |
| SF3B6 | CDC73 | BAG2 | NOL6 | DNTTIP1 |
| DLD | SLC25A4 | PALLD | SNW1 | MYL6 |
| ACAT1 | NUDT21 | EIF2S1 | CAV1 | SLC25A22 |
| SEH1L | LEMD3 | RPS27L | SIN3A | ABT1 |
| DNAJB1 | MFAP1 | MAP4K4 | GDI2 | MMTAG2 |
| PALLD | KRAS | TRIM41 | DDX39B | PRMT1 |
| GNAI2 | CYFIP1 | FDFT1 | PALLD | AFG3L2 |
| CYFIP1 | SEH1L | TXNL1 | SLC25A11 | MRPL38 |
| KRT6B | GTF3C5 | MRTO4 | CDK11B | NOP14 |
| CYB5R3 | MISP | AGK | CDSN | WDR43 |
| RAB11A | GPI | MPG | ACTR3 | CDK12 |
| RANBP1 | CHTOP | TAOK1 | WDR82 | LYZ |
| YWHAB | MTHFD2 | ACOT7 | ARID1A | ALAD |
| SRP72 | INTS3 | MAP4 | NT5DC2 | LUC7L3 |
| DDX39B | AFG3L2 | MEN1 | LLGL2 | NSD3 |
| PHC2 | BBX | TMEM214 | SSR4 | RPL36AL |
| CAPRIN1 | HSPA4 | DARS1 | BAZ2A | TRIP6 |
| SNU13 | SEPTIN7 | CAV1 | TOX4 | NDUFB4 |
| PDS5B | SLC25A22 | ATP5MG | KRT80 | SETD1A |
| SSR4 | FOXK2 | ORC4 | POLR1G | EMG1 |
| CALD1 | SEPTIN11 | ZMYM4 | ILKAP | LBR |
| NPEPPS | NSUN5 | INTS3 | SMARCB1 | MEN1 |
| ZC3H11A | HARS1 | RAB7A | CPNE2 | CMSS1 |
| YTHDC1 | CCDC86 | SLIRP | MCM3 | RPLP2 |
| AP2A1 | CPSF3 | PDLIM7 | TRIOBP | EIF2S1 |
| RPL22 | PSMA7 | HCFC1 | ITPR3 | SYNE2 |
| DPF2 | DHX37 | NDUFB10 | SMARCE1 | SCAF8 |
| BAZ1B | MCM6 | CD44 | NSUN5 | NOB1 |
| HK1 | ZNF207 | HADHB | MAP4 | CPNE2 |
| SMARCD1 | MCM5 | TFCP2 | PSMA3 | INTS10 |
| VCL | EPRS1 | NOC3L | ARPC4 | SAMHD1 |
| TRAP1 | ARF3 | EEF1A2 | ZMYM4 | CCNK |
| GATAD2B | HEATR1 | RGPD3 | TRIM72 | TAF4 |
| HSPA4 | PEBP1 | DPF2 | PC | PHLDA2 |
| RPS7 | SMCHD1 | MSN | RPL22 | BEND3 |
| GATAD2A | FDFT1 | CEBPZ | RBM27 | EIF4E2 |
| DDX51 | PALLD | S100A9 | MRPS7 | OXA1L |
| ARF3 | SLC25A24 | DDX39B | TPM4 | TPR |
| FDFT1 | MAFG | LEMD3 | RANBP1 | DAD1 |
| PRORP | TRIM72 | FAF2 | ARF4 | SMARCD1 |
| SUGP2 | ZC3H11A | PSMB5 | PPHLN1 | PRPF4B |
| LBR | ARID1A | ABCF3 | PSMB1 | TNKS1BP1 |
| SLIRP | AP2B1 | RRP1 | ANXA3 | NDUFA6 |
| SND1 | DNAJA1 | PSMA6 | NCOA5 | CDYL |
| NUP54 | NDUFB10 | DIMT1 | BRD2 | SKI |
| KPRP | NQO1 | SLU7 | VAT1 | PSMC5 |
| TNPO1 | RPS24 | RPL30 | TERF2 | ZBTB7B |
| FAU | ARPC2 | RPS24 | RAE1 | SPATA5 |
| PSMA1 | SAMM50 | TBL1XR1 | CPSF3 | S100A8 |
| CDC27 | YES1 | ZNF207 | RRP1 | INTS7 |
| MCM5 | TP53BP1 | NELFA | RAB7A | FXR1 |
| RAC1 | NOP9 | EIF6 | SLC25A24 | BAZ2A |
| CBX5 | HDAC2 | EMG1 | CAPZB | SSRP1 |
| DHX37 | HTATSF1 | RNF40 | CHAMP1 | ORC2 |
| MVP | TOR1AIP1 | CALD1 | ACTR2 | ZNF207 |
| CCDC50 | RAB11A | IMPDH2 | KRI1 | TFAM |
| RPS27 | CDH3 | CEBPB | PSMA4 | ITGB4 |
| NCBP1 | SDAD1 | CDCA7L | ARPC2 | PPIL4 |
| RAB1B | SRRM1 | FLG | LAD1 | ATP2A2 |
| NSUN5 | FAM98A | TRIP6 | RPS28 | AIFM1 |
| SNW1 | SMN1 | MACROH2A2 | EEF1A2 | SART3 |
| SFSWAP | KPNA1 | AP2B1 | EMG1 | PHF3 |
| EPRS1 | CNN3 | EIF5AL1 | AKAP8L | KDM1A |
| TP53BP1 | NFIX | INTS7 | DYNLL1 | AGPAT5 |
| FEN1 | SRPRB | NUP35 | PPP1R9B | KAT7 |
| DDX47 | PDP1 | RMI1 | HMGN1 | SPTY2D1 |
| RAD21 | INTS6 | RNPS1 | GTF3C4 | WDR46 |
| MYH10 | CCDC50 | CNP | BOP1 | CAV1 |
| CDK9 | SSR4 | DDX31 | PRDX6 | RBMX2 |
| PHLDB2 | TRAP1 | SEPTIN7 | ZC3H18 | SEC22B |
| NOP9 | SFSWAP | SMCHD1 | MRPL11 | KPNA6 |
| STIP1 | BOP1 | GNAI1 | DIS3 | HDAC2 |
| SEC11A | KRT84 | RPL37A | SEC11A | PHGDH |
| TOP3A | CPNE2 | RSL24D1 | RPS15 | NDUFA13 |
| KRT78 | PSMD2 | MCM2 | ATP5PB | ZNF48 |
| CAVIN1 | TPM4 | ZC3H14 | RPS7 | CBLL1 |
| EEF1A2 | FAU | RO60 | ARGLU1 | FBXO11 |
| PSMD3 | FOXK1 | TNPO1 | CSNK2A1 | RRP7A |
| CCAR1 | ETF1 | TEAD4 | FLNC | PGK1 |
| HDGF | NUP43 | POLR2E | CSTF1 | SYPL1 |
| ESRP1 | DDX39A | CKAP4 | AZGP1 | DNMBP |
| DARS1 | RBM28 | NSA2 | SLC25A22 | SCRIB |
| TMA7 | WDR5 | DCD | MTCH2 | DNAJA3 |
| NIPSNAP1 | RAB1B | ARF3 | PLRG1 | IDH3A |
| RRS1 | CTBP2 | UTP14A | DHX30 | NELFA |
| DNTTIP1 | RPL19 | PABPN1 | CALD1 | CHCHD3 |
| RPS28 | RPL36 | FXR1 | RBBP7 | DGCR8 |
| CEBPB | RPL22 | LCN1 | TRAP1 | ZGPAT |
| JUND | RBM3 | SUN2 | CBARP | DPM1 |
| LENG8 | CFAP20 | TASOR2 | GNAI1 | PRORP |
| MCM2 | RRAS | LUC7L3 | TERF2IP | SMN1 |
| ETF1 | DRAP1 | YWHAB | RNF40 | SNTB2 |
| UBA1 | TNPO1 | GLG1 | LBR | RCC2 |
| MYL12B | LARP4 | NR2F1 | CTCF | CLK3 |
| MTX1 | CD44 | ZC3H18 | PSMD2 | TAF6 |
| AAAS | RANBP1 | FLOT1 | CFAP20 | ETV6 |
| CFAP20 | DNAJB6 | IDH1 | LCN1 | URB2 |
| PHGDH | RAE1 | POP1 | GLUD1 | DCXR |
| RPS24 | YWHAB | LBR | CAPZA1 | PML |
| LRWD1 | NUP35 | U2AF1 | UQCR10 | BRD2 |
| AIFM1 | FKBP4 | CSTF1 | SDAD1 | MED16 |
| TBL1XR1 | MTX1 | LUC7L2 | LUC7L3 | LGALS7 |
| SLC25A22 | SEC61A1 | SNW1 | FAU | CSTA |
| NDUFB4 | TPP1 | INTS13 | EIF6 | SCAF4 |
| GLUD1 | NR2C2 | YY1 | BRD4 | HBE1 |
| SLC25A10 | CAVIN1 | ARPC2 | PSMA6 | EED |
| RTN4 | SPOUT1 | TIA1 | MAP4K4 | DEK |
| DNAJB6 | RPF2 | HIRA | EIF4H | PRPF38A |
| PDLIM7 | RAVER1 | HDAC2 | PAK1IP1 | AQR |
| EIF5B | TFRC | CS | GSTO1 | SRBD1 |
| CAPZB | TBL1XR1 | PDHB | LYZ | MTCH1 |
| CWC22 | SUN1 | PSMA4 | ARF6 | IPO5 |
| PFKL | CFL1 | WDR76 | FLOT1 | EHD2 |
| KPNA1 | DLST | TUBA1A | PABPN1 | PCM1 |
| BRD2 | UQCR10 | SF3A2 | DNTTIP1 | VEZF1 |
| ACSL3 | EXOSC4 | PFKL | CAT | TBL2 |
| CAV1 | ORC2 | DNAJA1 | AFG3L2 | BAIAP2L1 |
| EPCAM | MVP | HARS1 | VCL | ZNF687 |
| DDX41 | ACSL5 | GLUD1 | GMPS | POLRMT |
| PARN | LBR | YARS1 | CSTF2 | CTR9 |
| TOMM22 | DIMT1 | PGAM1 | DARS1 | USP36 |
| GTF2F2 | GART | TRIM72 | POLR2A | TFPT |
| ACTN2 | TRIM41 | HDGF | LRWD1 | MPRIP |
| GSTO1 | BTF3 | ABT1 | TFIP11 | MPHOSPH10 |
| TPP1 | RPS7 | TBPL2 | RAB1A | MCM5 |
| SRPRB | SUN2 | MVP | SKI | KRT80 |
| DNTTIP2 | JPT2 | HMGN1 | CTSD | DHX33 |
| MORF4L1 | ARPC1B | ZNF462 | GTF3C3 | YWHAZ |
| SUZ12 | WRN | PABPC4 | DAP3 | METTL15 |
| ACADM | MRPL11 | DDX39A | RAVER1 | TOR1AIP1 |
| TMEM214 | RTN4 | SFSWAP | SLIRP | RAB7A |
| CD44 | ALDH1B1 | TWNK | COX6C | ZCCHC7 |
| DECR1 | G3BP1 | VEZF1 | PDIA6 | TUBB3 |
| SUN1 | TXNDC5 | NOB1 | CDK9 | SLX9 |
| NUP35 | ACOT7 | DECR1 | NCCRP1 | NACC1 |
| DAP3 | GLYR1 | NOL10 | RBM28 | ACSL3 |
| PML | CDCA7L | PSMC5 | PSMD11 | DMAP1 |
| CPSF4 | DCD | DNAJC8 | CNP | ZNF148 |
| TBCK | CCAR1 | MBNL1 | NDUFB4 | PSMD6 |
| YWHAQ | LAD1 | ATP5PB | OXA1L | CCDC86 |
| RBM28 | ITPR3 | DLD | CDC23 | DBNL |
| ABCF1 | PSMC5 | CAVIN1 | FAF2 | TK1 |
| WDR5 | COX20 | SCAI | PYCR2 | SPATS2L |
| CNP | RAD21 | XP32 | NDUFA6 | BAIAP2 |
| MPG | TOMM70 | EXOSC2 | RAD21 | NYNRIN |
| PSMA4 | RPL30 | ETF1 | FXR1 | PSMD8 |
| SAP18 | SART3 | FAM98B | ALDH3A2 | TPI1 |
| EXOSC4 | MACROH2A2 | DCXR | RPL37A | CNOT2 |
| ORC2 | CS | PDAP1 | SUN2 | UBP1 |
| SPCS2 | ACADM | CORO1C | IDH1 | CELF1 |
| MAFG | FLOT1 | NDUFS4 | DPF2 | ZNF146 |
| DIS3 | NDUFB4 | FOXC1 | GTF2F2 | ACADM |
| TRMT10C | WDR12 | MRPL45 | SART3 | MRPL37 |
| IDH1 | AP2A1 | PFN1 | CCAR1 | LTF |
| GLG1 | RPL7L1 | PYCR2 | HMCES | PSIP1 |
| ARF6 | ESRP1 | RPS28 | NUP43 | MRPS18B |
| TUBB3 | SEC11A | LRWD1 | NHP2 | RIF1 |
| SKI | NOB1 | RAB11A | PHC2 | ACSL5 |
| DLST | PHF8 | MORF4L1 | HSPH1 | GLG1 |
| EIF4H | SSR1 | ORC2 | BAG2 | NME3 |
| ZMYM4 | ETV6 | SMPD4 | MFAP1 | PRSS3 |
| TRIM41 | CBX5 | EHF | XRCC1 | CCAR2 |
| APOBEC3B | RPS28 | METAP2 | EHF | POLR2E |
| RAVER1 | VEZF1 | DDX19B | NPEPPS | RBM33 |
| CEBPZ | NUP37 | TEFM | CHD7 | TUBB2A |
| BPTF | TRIP6 | ANXA4 | NR2C2 | TCF20 |
| DIMT1 | CDK9 | CBFB | EIF3A | DYNLL2 |
| MTCH2 | CAPZB | HK1 | MISP | PSMC6 |
| BOP1 | ACTR2 | ACSL3 | RAI14 | CORO1B |
| INTS13 | CLIC1 | PUM1 | PRPF31 | ANAPC5 |
| TOMM70 | NFAT5 | ESRP1 | DLST | BCL7C |
| NDUFC2 | HMGN1 | MRPS23 | KRT15 | NOP53 |
| DRAP1 | ABCF1 | NFATC1 | DDX51 | CPT1A |
| HSPH1 | PRPF31 | S100A8 | YES1 | RHOT1 |
| DDX39A | NIPBL | GADD45GIP1 | JPT2 | BSG |
| PLIN3 | PDS5B | TJP3 | TPX2 | UBR5 |
| GNAI1 | TERF2 | TMEM43 | SRPRB | FEN1 |
| THOC2 | SIN3A | CPNE2 | CYFIP1 | USP10 |
| NSUN4 | S100A9 | LYN | ATP5MG | NOL7 |
| PPFIBP1 | LUC7L3 | AKAP12 | ZBTB20 | NUP88 |
| DNAJC8 | TEX10 | NTHL1 | IGF2BP2 | KRT4 |
| TRIP6 | PRDX2 | INTS10 | STMN1 | NOP10 |
| ATP5MG | MORF4L1 | ACADM | YWHAQ | HBS1L |
| FKBP4 | CDC23 | NOL7 | RING1 | POM121C |
| SDAD1 | DNAJB1 | SIN3A | ANAPC1 | MCM6 |
| NFIX | SLIRP | SEC61A1 | DNAJC8 | TNPO3 |
| TPM1 | CSTF1 | PTPN1 | MAFG | RPS6KA4 |
| RBM7 | GDI2 | PML | HSD17B10 | COIL |
| TARS1 | TAOK1 | KRT4 | ZC3H14 | ATXN2L |
| RAB13 | MTCH2 | GNAI3 | MRPL38 | NOL8 |
| KRT80 | GLG1 | DLST | PIP | CNOT9 |
| NDUFA6 | PSMC1 | UQCRQ | CRNKL1 | THAP11 |
| PHF5A | ATP5MG | MSANTD4 | PBRM1 | UQCRFS1 |
| CKAP5 | ANGEL1 | PSMD2 | MYO6 | SRP68 |
| CCDC47 | AIFM1 | TFRC | GSTP1 | ISY1 |
| POLR1A | PTBP3 | POLR1B | PFKM | CHTF18 |
| RO60 | GTF2F2 | GNL2 | ACADM | ZCCHC8 |
| NSD3 | CDYL | EIF4E | TPP1 | MCU |
| G3BP1 | TRMT10C | CWC22 | MTX1 | NME1 |
| SNRPB2 | PUM1 | MMTAG2 | PSMC5 | MRPS26 |
| ITPR3 | DDX19B | AP2M1 | DSC3 | MAGT1 |
| CDC16 | ZC3H18 | C14orf93 | CD2BP2 | MTMR2 |
| VEZF1 | RAB35 | PSMC1 | NDUFA12 | GFPT1 |
| CPSF3 | EIF4E | BBX | RPS24 | CYB5R1 |
| ANAPC1 | SPCS2 | NOP16 | TARS1 | ACACA |
| EZH2 | THOC2 | PHGDH | SEPTIN10 | TPM4 |
| PSMC4 | PSMA3 | TEX10 | PHLDB2 | NFRKB |
| CKMT1A | C19orf53 | WDR74 | RAD23B | FUS |
| DMAP1 | SLU7 | PELP1 | DDX39A | SLC16A3 |
| MRPL11 | MEN1 | WDR43 | RAB2A | BPTF |
| HNRNPLL | PRDX6 | EZH2 | PRKCSH | TMEM11 |
| PSMC5 | SNW1 | PDIA6 | TUBG1 | SSR3 |
| DCD | SKI | TOMM22 | CS | TP53BP1 |
| SSB | FAF2 | SFMBT1 | GATAD2B | RALB |
| CDH3 | FABP5 | KRT13 | SFSWAP | ATP5MG |
| YARS1 | ACSL3 | C11orf98 | SDHB | RRP8 |
| BTF3 | PAICS | EIF2S3 | CLIC1 | NUMB |
| EHD2 | BZW1 | PAK1IP1 | SPCS2 | EEF1A2 |
| CSNK1A1 | PARN | NUP43 | RPL36AL | USP6NL |
| DNAJA1 | CAPZA1 | SLC25A10 | PLIN3 | ADD1 |
| FLNC | USP7 | AP2A1 | TFRC | SUPT6H |
| RHOG | LRWD1 | COIL | AGPAT5 | PATZ1 |
| PSMB1 | NELFA | CLOCK | MRPS22 | CCT6A |
| UQCRQ | CEBPZ | NDUFA6 | EZH2 | RBM4B |
| SLU7 | URB1 | H1-1 | TNPO1 | TAF6L |
| S100A8 | CDC27 | RAB13 | ORC2 | PPFIA1 |
| NOC4L | IPO7 | RNF2 | IPO7 | DSC2 |
| ALDH1B1 | RGPD3 | ACSL5 | PFKL | PRDX2 |
| NELFA | SUGP2 | MGST3 | ARPC5L | AATF |
| GSTP1 | PLIN3 | PTCD3 | BAIAP2 | RAB1B |
| EHF | NDUFS4 | DDX56 | NUDC | SLC25A24 |
| G6PD | TFCP2 | FXR2 | AP2M1 | GANAB |
| PSMD6 | ABCF3 | GSTO1 | DNAJA1 | LRWD1 |
| SART3 | POLR2E | MRPL16 | CDC16 | TJP2 |
| PABPC4 | PDIA6 | BEND3 | LSM4 | GATAD2B |
| PAF1 | DCXR | FABP5 | INTS3 | INO80 |
| EIF3E | AGK | MRPL37 | RBM7 | TUBA1C |
| TUBG1 | CRNKL1 | BRD2 | CBX1 | ACTN1 |
| TEFM | PELP1 | HDAC6 | MBD2 | SPECC1L |
| PPAN | RHOG | DNAJA3 | NIPSNAP1 | DECR1 |
| RAB5C | RO60 | PNKP | TXNL1 | EDC4 |
| ESF1 | RPS27L | RALB | GPD2 | GGCT |
| MRPS23 | LYN | NIP7 | RPS27L | SRP72 |
| ZBTB20 | YTHDC1 | DDX55 | TOP3A | RO60 |
| ARHGDIA | NFRKB | C1QBP | HEATR1 | NFIX |
| RPS27L | NKRF | ABCF2 | TFCP2 | AKAP8L |
| POLR2E | VAMP3 | GATAD2B | CMAS | DCAF13 |
| RGPD3 | PHF5A | SPOUT1 | TBL1XR1 | STOML2 |
| MEN1 | TMEM214 | RTN4 | PRDX4 | TCP1 |
| TPM4 | ALDH3A2 | SEC11A | EIF5B | CBX4 |
| PTCD3 | RAB5C | SUGP1 | PDCD6 | NDUFB5 |
| CAMK2D | RDX | BMI1 | MRTO4 | DDX42 |
| HDLBP | ILKAP | CLPX | SNRPB2 | CCT7 |
| PAK1IP1 | IDH3B | AATF | FKBP3 | NDUFB10 |
| SLC25A24 | C14orf93 | DNAJA2 | CD109 | VAPA |
| LAD1 | YWHAQ | TASOR | PAICS | EIF5AL1 |
| CCDC86 | FOXC1 | CTBP1 | NUP54 | MRPL9 |
| GIGYF2 | RAB2A | SND1 | ETF1 | C11orf98 |
| EXOSC2 | TK1 | RCOR1 | DMAP1 | MGST3 |
| VAMP5 | NOP14 | DHX38 | RBBP6 | GNA11 |
| MRTO4 | HOXB6 | PHF8 | TCF20 | FOXC1 |
| UTP14A | PSMD3 | ESF1 | COX20 | SLTM |
| ZBTB43 | NPEPPS | NOL11 | TRMT10C | STRBP |
| PDHB | GATAD2B | SAP30BP | DRAP1 | MCM2 |
| SCAI | CALD1 | NOC4L | YY1 | SNRPF |
| SEPTIN10 | RAB13 | LSM4 | DECR1 | MRPS22 |
| CRNKL1 | GATAD2A | KPNA6 | ATP2A2 | EIF2AK2 |
| SMPD4 | C5orf24 | CTSZ | JUNB | PHC3 |
| POMZP3 | S100A6 | CHAF1A | PPFIBP1 | CCDC137 |
| USP7 | DMAP1 | SLX9 | HYOU1 | SEC23B |
| RHOT1 | ARG1 | UTP4 | YBX1 | RAB2B |
| NOP16 | MPRIP | PSMD6 | SURF6 | RAB11A |
| CSRP1 | PUM3 | SEC61B | NKRF | EPHA7 |
| NUDC | PSMD6 | TUBB4A | NME3 | DDX39B |
| PHF3 | MCM2 | ALDH1A1 | PUM3 | UTP23 |
| PDCD6 | PSMD11 | EMILIN1 | FAM98B | ERLIN1 |
| PDP1 | SND1 | MOGS | ORC4 | TPX2 |
| SRCAP | BRD2 | STT3B | PLXNA2 | UPF1 |
| ATP2A2 | PDAP1 | IMP4 | MORF4L1 | HADHA |
| ALDH3A2 | EIF4A2 | ORC1 | DPYSL2 | TMCC3 |
| GPD2 | KPRP | ATP1B3 | HEXIM1 | GRHL2 |
| DCXR | CBX1 | TUBB3 | XP32 | XPO4 |
| ITGB1 | RCL1 | LYZ | WDR12 | MGA |
| HEATR1 | MPG | PRDX2 | ERH | CDSN |
| PTPN1 | CTBP1 | PSME3 | HSPE1 | TEFM |
| PDIA6 | CWC22 | NRF1 | STT3B | STRN3 |
| BAIAP2 | SAMHD1 | RAB1B | INTS13 | GNAI3 |
| SPTY2D1 | RBM4B | RPS19BP1 | PHGDH | ANAPC4 |
| NFIA | U2AF1 | ACTR2 | YWHAH | CPNE7 |
| RAD18 | NDUFA7 | NME3 | TOMM22 | CCDC71L |
| HEXIM1 | EIF3A | TOMM70 | DDX31 | CSNK2A2 |
| LSM4 | C11orf98 | EIF5B | CSRP1 | HSD17B11 |
| EHD1 | UTP14A | AIFM1 | NOB1 | BLMH |
| GMPS | PTCD3 | NXF1 | C11orf98 | SAMM50 |
| MRPS22 | TBRG4 | EHD2 | SERPINC1 | SEPTIN2 |
| PGM5 | EIF3B | SMARCD1 | ERP29 | SF3B4 |
| SEC61A1 | RPL36AL | CPNE7 | ACSL5 | GLIPR2 |
| FKBP3 | RNF2 | NUP54 | DCXR | ONECUT3 |
| DDX19B | CSRP1 | ERCC2 | CHAF1A | PTCD3 |
| PDAP1 | TASOR2 | NUCKS1 | RAB11A | MVP |
| VAPA | CNP | ZC3HC1 | BAZ1B | SCD |
| MGST3 | G6PD | HMG20B | TRIP6 | LEMD2 |
| ERLIN1 | MTAP | ZNF768 | CELF1 | STAG1 |
| CAP1 | ORC4 | ALMS1 | MEN1 | HLA-A |
| TJP3 | HDLBP | NDUFA11 | CAP1 | CENPF |
| EPS8 | BAIAP2 | INO80C | HDLBP | LUC7L |
| CTBP1 | PYCR2 | YES1 | ACTR1A | SLC3A2 |
| WDR43 | FAM98B | WDR75 | HNRNPLL | CS |
| RAD23B | MGST3 | RPL32 | INTS6 | SFMBT1 |
| TALDO1 | MMTAG2 | TPX2 | VAPA | ESYT2 |
| ABLIM1 | GSN | RBM28 | RCL1 | TFAP2D |
| CSNK2A2 | TJP3 | ZBTB7B | PABPC4 | MAP4 |
| TMEM43 | SEC61B | PLEKHA5 | MAPRE1 | YBX3 |
| EIF3B | DECR1 | RHOC | DNAJA3 | RBBP6 |
| FOXC1 | FOXJ3 | PAF1 | ABCF1 | LLGL1 |
| DPYSL2 | COPA | THOC2 | CCDC86 | ZBTB10 |
| WDR12 | NOM1 | ARL1 | AIFM1 | ARPC5L |
| NUP37 | MAFK | ERLIN1 | WRN | PLEKHG3 |
| RCL1 | RPS15 | GSTP1 | DDX50 | PSMD4 |
| PSMA3 | UQCRQ | EIF3A | EIF4A2 | LZTS2 |
| RPRD1A | INTS13 | IDH3B | NDUFB3 | API5 |
| CELF1 | ZIC2 | RAD21 | NFATC1 | ARL6IP6 |
| PDIA5 | PDCD6 | SYPL1 | MVP | LASP1 |
| COX20 | DAZAP1 | EHD1 | WTAP | ZBTB2 |
| GADD45GIP1 | NDUFB5 | VAPA | RTN4 | PARN |
| EIF4A2 | NSD3 | COPA | LEMD3 | SETX |
| UPF1 | GADD45GIP1 | RCL1 | CUL1 | IQGAP1 |
| TOE1 | SCAF11 | CDYL | CCDC137 | TOP3A |
| TPX2 | GSTP1 | ABLIM1 | PAF1 | NTHL1 |
| TFPT | RHOT1 | GATAD2A | NFATC2 | NUP54 |
| NCBP3 | RAP2B | CHD6 | UTP14A | CCT4 |
| DHCR7 | ERCC2 | SMARCAL1 | MTCH1 | WIZ |
| FAM98B | IMP3 | PRPF38A | CBX5 | IDH1 |
| ARPC5 | PHGDH | CEP131 | LYN | ZNF384 |
| DRG1 | INTS10 | MGA | PTPN1 | YWHAQ |
| NDUFS4 | MLEC | INTS14 | PGD | RBM15B |
| C11orf98 | SEPTIN10 | GDI2 | MGST3 | MKRN2 |
| TRMT1L | FOSL2 | HOXB4 | MPRIP | LYN |
| CD109 | TUBA1A | MBD2 | POF1B | RAB5A |
| RPS15 | CELF1 | NMNAT1 | U2AF1 | PNO1 |
| TFRC | IARS2 | SEPTIN10 | SENP3 | TKT |
| TUBA1A | LSM4 | CXXC1 | CPSF4 | ABCF2 |
| SUMO2 | DPF2 | DR1 | RHOT1 | PSMB1 |
| LARP7 | WDR43 | TCEA1 | SNIP1 | RAB14 |
| LETM1 | RAB14 | MRPL9 | UPF1 | PSMD7 |
| LAS1L | NDUFA6 | ARPC5 | GATAD2A | VIRMA |
| RFX5 | POLR1A | ENTR1 | UBE2V1 | MLEC |
| NDUFA4 | RBMX2 | RPRD1B | LTA4H | MDH2 |
| SCAF11 | TFAP2B | MPHOSPH10 | ATP1B3 | MRPS14 |
| PCID2 | NUP188 | PUM3 | FARSB | UTP11 |
| HIRA | SPATA5L1 | SLC16A3 | INTS10 | KDM1B |
| NME3 | MYC | URB1 | ARPC5 | LRPPRC |
| PSMB6 | CDC16 | PRPF31 | ANXA4 | LRCH3 |
| HBS1L | FXR2 | PKP4 | CTR9 | STT3A |
| EIF3CL | ANAPC1 | YWHAQ | NUP37 | RBM19 |
| RBM42 | HIP1R | OGT | SSB | CAPZA2 |
| TFCP2 | HSD17B10 | CSRP1 | SAP30BP | POP4 |
| NSA2 | TBPL2 | MACROD1 | SUZ12 | RTN4 |
| RAP2C | NOP16 | MRPL4 | MLEC | CISD1 |
| MBD2 | LETM1 | CAPZA1 | ZNF609 | APOC3 |
| CHAF1A | DDX31 | KMT2A | EXOSC4 | FNBP4 |
| TASOR2 | TRMT1L | TBL2 | TOMM70 | IWS1 |
| GART | GNA11 | PCNA | BLM | RTCB |
| CTIF | RBM7 | MORF4L2 | SLU7 | APOBEC3B |
| ANAPC2 | NUP54 | ATRX | MARK2 | CLASP2 |
| HIP1R | RBM15B | MRM2 | WDR75 | BANP |
| STMN1 | PSMA4 | ADD3 | RPL32 | PYCR2 |
| FXR2 | RALA | EPS8 | EXOSC2 | SRPK2 |
| MRPL16 | BYSL | SLC25A24 | DDX10 | ACTR8 |
| SLX9 | PABPC4 | ITPR3 | ANP32B | SMC5 |
| ZNF644 | JUND | CTR9 | SEC61A1 | CAT |
| NDUFA12 | PPFIBP1 | NUP50 | ACSL3 | ITPR3 |
| AHDC1 | GMPS | FKBP8 | GLIPR2 | PSMD14 |
| PUM3 | PSMA6 | ATP2A2 | RBM15B | ZKSCAN1 |
| FARSB | BTAF1 | MRPL11 | GRSF1 | UFD1 |
| NRF1 | MRPS18B | HDGFL2 | COTL1 | THOC2 |
| BZW2 | NOL10 | LONP1 | PHF5A | UTP6 |
| ADD3 | PLCB3 | SPCS3 | EHD1 | KIF18A |
| ELF1 | PCGF2 | EXOSC6 | NDUFS4 | ARL6IP5 |
| RPL7L1 | RPL35 | GSR | NACC1 | GATAD2A |
| PSMB7 | MGST1 | JUND | RPL7L1 | TIMM50 |
| ZNF768 | SERPINB12 | MPHOSPH8 | KPNA1 | KLF5 |
| MRPS18A | CTR9 | TRAP1 | ETFB | RAB10 |
| ABCF3 | WTAP | USP3 | DAZAP1 | CTDSPL2 |
| MAFF | DNAJC8 | RRP15 | MT-ATP6 | PSMD1 |
| FLOT1 | C1QBP | INTS5 | SPOUT1 | DROSHA |
| NKRF | TWNK | GRHL2 | FKBP8 | KRT23 |
| PPP1R9B | COTL1 | NPEPPSL1 | PALM | CCT2 |
| WRN | CAT | COX7A2 | NDUFB5 | UQCRQ |
| TASOR | WDR18 | MGST1 | NDUFA9 | PSMC3 |
| NUCKS1 | LYST | MRPS2 | DIMT1 | ZNF609 |
| SLFN5 | DRG1 | CRNKL1 | HIP1R | DNAJB1 |
| SNRPF | MRPS23 | TAF2 | GSN | HEXIM1 |
| PSMD1 | PTPN1 | DNAJB6 | MRPL16 | EXOSC2 |
| SEC61B | UPF1 | CDC16 | SAP18 | KRT7 |
| PDCD6IP | PCID2 | NOL6 | RALA | TASOR2 |
| GSN | ARPC5L | RAP1A | TOE1 | TMED7 |
| MTHFD1L | NDUFA9 | SRPRA | SERPINB3 | WRNIP1 |
| CPNE7 | YARS1 | LAMP1 | FKBP4 | NT5E |
| NDUFS7 | NDUFS7 | HOXB9 | GPRC5A | UTP15 |
| RNF40 | EPS8 | TIGD2 | THOC6 | GALE |
| MAOA | STMN1 | UCHL5 | STAG1 | REEP4 |
| MACROH2A2 | VCL | RPS6KA4 | TIA1 | HDAC6 |
| CUL1 | WDR75 | TK1 | GTF2F1 | EIF3A |
| POLR1B | CSNK1D | CCNK | SSR1 | CYB5B |
| IDH3B | MRPL13 | CREB1 | RAP2A | PARD3 |
| RBM33 | MAP2K3 | MYO6 | CEBPZ | NLE1 |
| SYPL1 | ATP1B3 | EIF3E | SCD | RHOF |
| EIF4G1 | RBBP7 | TARS1 | MRPL19 | AP1B1 |
| GFPT1 | ERLIN1 | NOM1 | MYOF | TASOR |
| MAGT1 | CXXC1 | NIFK | ZNF148 | NACA |
| NIFK | CREB1 | NOP14 | TEFM | COPA |
| EXOSC3 | RBM33 | GTF2F1 | VPS35 | RNGTT |
| GPRC5A | GLIPR2 | SAP18 | ERLIN1 | TAF1D |
| MRPL3 | ONECUT3 | OXSR1 | NELFA | CEP55 |
| NDUFB3 | ZNF609 | THAP11 | TCEA1 | PSMA7 |
| SMN1 | GPD2 | GTF2F2 | GADD45GIP1 | GOLGA2 |
| ETFA | ATP2A2 | PRSS3 | PTGES3 | DARS1 |
| NFIL3 | CETN2 | JUN | WDR5 | HSP90B1 |
| SCD | CHD6 | MT-ATP6 | NOL10 | P3H4 |
| SORD | MRPS34 | EIF3CL | PSMD8 | BTAF1 |
| RPL32 | EHF | IMP3 | NOMO2 | VPS72 |
| WDR55 | PSMD8 | PFKM | ACOT7 | CPNE8 |
| GNL2 | SAP30BP | WAPL | AP2S1 | NCOR2 |
| FOSL1 | ACTR8 | RBM15B | CDCA7L | ALDOC |
| FARSA | KIF23 | YBX3 | GIGYF2 | STAG2 |
| NARS1 | DIS3 | PPAN | NXF1 | PKP2 |
| CLP1 | SORD | MAGT1 | DRG1 | CCNT2 |
| KPNA6 | NRF1 | RRAS2 | MDN1 | SPATS2 |
| RBM4B | PRKAR1A | TXN | TASOR2 | FCF1 |
| PPIL4 | YWHAH | BCL7C | RHOC | MAPRE1 |
| MBD3 | RAB6A | AP5M1 | GART | MED27 |
| NDUFA11 | PLD3 | NKRF | VASP | RDH13 |
| DDX56 | LTA4H | DSC1 | LETM1 | PKP4 |
| MTCH1 | MRPL3 | TRIM4 | IDH3B | KPNA4 |
| ACTR2 | MOGS | SUPT6H | SUGP1 | C8orf33 |
| UQCR10 | S100A8 | PHLDA2 | AP2A2 | PLD2 |
| NOM1 | MRPL19 | VRK2 | ABCF3 | YWHAB |
| FLG | TENT4B | CLIC1 | ARHGDIA | TAF15 |
| SMC2 | DNAJA3 | CCAR1 | PDAP1 | CLOCK |
| FDPS | KIF2A | PDIA4 | ANP32A | VAPB |
| ANP32B | ARPC5 | MLEC | PSMB6 | GTPBP6 |
| SEC22B | GSR | TFDP1 | TMEM214 | CHD7 |
| DCAF7 | FLG2 | SSR3 | EIF3CL | ERBIN |
| PSME1 | PPAN | KIF2A | G6PD | ABI2 |
| MMTAG2 | METAP2 | H2AC20 | EXOSC6 | CAD |
| POLR2H | TMA16 | PSME1 | RDX | NUFIP1 |
| ORC4 | INTS5 | CBLL1 | DNM3 | CALD1 |
| PTBP3 | EHD2 | SPATA5L1 | CWC22 | NCBP2 |
| HLA-A | SEC22B | SF3B6 | CAPZA2 | ZC3HAV1 |
| SNIP1 | POLR1C | MAZ | NDUFA4 | PWWP3A |
| TBL2 | KPNA6 | TGIF1 | MOGS | P4HB |
| NCKAP1 | VPS35 | USP10 | DDX19B | POF1B |
| NFRKB | DDX10 | VAT1 | SMPD4 | MED1 |
| NOMO2 | NSF | SSB | TBRG4 | BRMS1 |
| PPP3CA | INTS12 | DCAF7 | UTP25 | EPRS1 |
| CNOT1 | RPL15 | ANAPC2 | SYPL1 | USP30 |
| BUD13 | EHD1 | GTF2B | CHEK2 | KANK2 |
| EIF4E2 | UFD1 | VCL | AP2A1 | ZC3H11A |
| COX7A2 | PSME1 | ZNF512B | PSMD12 | UHRF1 |
| SCO2 | NUP88 | AHDC1 | NDUFS7 | PLCD3 |
| UTP20 | EDF1 | ZNF609 | EIF4E | ZDHHC5 |
| DDX31 | EIF3D | SCO2 | CBFB | ACADSB |
| UFD1 | CPSF4 | DNMBP | ETV6 | ACSL4 |
| NDUFA7 | MAGT1 | ANO2 | WDR43 | EXOC2 |
| GDI2 | TGM3 | MPP7 | NUP35 | HNRNPU |
| RHOC | NFATC1 | NCBP3 | USP7 | H4C1 |
| PGD | LYZ | LLPH | GNA11 | HNRNPM |
| PSMD7 | RMI1 | CTDSPL2 | TMEM43 | H2AZ2 |
| LAMP1 | CAP1 | MAK16 | ACADVL | KRT14 |
| STRAP | PGD | PSMA2 | FXR2 | MYBBP1A |
| PRPF38A | NAMPT | PRRC2A | KIF2A | GTPBP4 |
| SLC16A3 | EXOSC6 | CDC37 | RAC1 | NOP58 |
| C14orf93 | CHAF1A | OGDHL | MRPL4 | NUP93 |
| PNKP | ABLIM1 | ZFP91 | IDH2 | RPL31 |
| SAMHD1 | POP1 | ABI1 | PSMA2 | NOLC1 |
| OGT | MRPL45 | LAD1 | ESF1 | DKC1 |
| LTA4H | ZNF185 | SOX4 | DPM1 | HMGA1 |
| FOXJ3 | SMPD4 | ESYT2 | TAF6L | RPL27A |
| ARPC5L | GPRC5A | FKBP3 | ZBTB43 | RPS15A |
| NUP50 | COIL | PHC3 | MRPL45 | SYMPK |
| PRKCSH | SYPL1 | ZCCHC8 | PSMB4 | PRPF40A |
| ANXA11 | VASP | DYNLL2 | BCCIP | RBM39 |
| NAMPT | RAB18 | MTMR2 | NSA2 | CEBPZ |
| MGST1 | TERF2IP | RECQL | SLC25A10 | PKM |
| TAF4 | AGPAT5 | ERC1 | ZBTB7B | PBRM1 |
| TECR | ABCF2 | TIMM50 | TRMT1L | TARDBP |
| HSPE1 | ACADVL | GMPS | PLCB3 | NUP85 |
| NCBP2 | ATAD1 | HEXIM1 | MTHFD1L | HSP90AB1 |
| GSR | WRNIP1 | BAZ2B | MYL6 | SLC25A3 |
| S100A13 | COX7A2 | TERF2IP | TEX10 | CDC27 |
| MRPL19 | SRP68 | TRIM33 | WDR18 | SMARCB1 |
| ETFB | NOC4L | TNKS1BP1 | METAP2 | RPL38 |
| GLIPR2 | GRWD1 | TRIM25 | UFD1 | LAS1L |
| WDR74 | MPHOSPH8 | ABCF1 | IRF2BPL | SAFB |
| PELP1 | HMG20B | TMCC3 | MRPS26 | LIG3 |
| IDH2 | TALDO1 | HACD3 | PRPF38A | KIFC1 |
| CTSZ | PAF1 | CUL1 | POLR2H | ANAPC1 |
| MED23 | TBL2 | EIF3B | TUBA1A | TRA2A |
| ATP12A | HSPH1 | GLIPR2 | LGALS7 | ZNF24 |
| STAG1 | PNO1 | HOXC10 | CNOT1 | RAP1B |
| MRPS18B | ADD1 | ARF5 | COPA | RPL13A |
| COIL | PRPF38A | SPATA5 | SCAF11 | ALDOA |
| G3BP2 | ECH1 | SLC25A12 | EIF4G1 | DLST |
| COPA | MCCC2 | VPS4B | PSMD1 | PSMC2 |
| BYSL | INTS8 | PRKD2 | RNF2 | RRP15 |
| QARS1 | NDUFS5 | ANP32B | BZW1 | DDX49 |
| EIF3D | RHOC | RAB6B | TJP3 | PSMC1 |
| GNPDA1 | APOBEC3B | BMS1 | PPAN | TBL1XR1 |
| DENR | WAPL | MRPL19 | NCBP2 | SUB1 |
| CDCA5 | HDGFL2 | TALDO1 | S100A13 | SEH1L |
| FBXO11 | USP6NL | HSD17B11 | PPP3CA | ARID2 |
| HMG20B | RAC1 | HNRNPLL | CDC37 | ERI1 |
| PSMB3 | CD109 | CBX4 | COIL | CDK11A |
| HOXB8 | NDUFC2 | PSMD12 | SETD1A | FOXK1 |
| RSL24D1 | PSMB3 | FOXJ3 | DHCR7 | SURF6 |
| CLPX | CUL1 | DHX33 | PHF2 | SUGP1 |
| THOC6 | MPP7 | TBRG4 | NCLN | PABPN1 |
| MED16 | UTP4 | RBM22 | STRAP | ARID1B |
| SDHA | DDX49 | CHD1 | FBXL12 | PLRG1 |
| NAP1L1 | DDX56 | BZW2 | OLA1 | IDH3B |
| CLIC1 | ZNF687 | INPP5K | MAK16 | RRAS2 |
| MACROD1 | HOXA9 | OCIAD1 | PSMD6 | RACK1 |
| TJP2 | PPP3CB | EIF2AK2 | NIP7 | BLM |
| RPRD1B | SOX4 | CRX | SF3B6 | NDUFS7 |
| ACTR5 | DPYSL2 | MAFK | NOL7 | PUM1 |
| ERAL1 | MDH1 | GALE | SUMO3 | CHAMP1 |
| INTS5 | SRM | HOXB8 | MRPL41 | PRDX4 |
| DHX33 | NSA2 | SERPINB12 | NFIA | YY1 |
| ACOT7 | ZNF146 | RPP30 | MAGT1 | BMI1 |
| MPP7 | FKBP3 | RAC1 | SAMHD1 | DNAJB6 |
| ZNF462 | MRPL54 | SAMHD1 | CLINT1 | ACTR1A |
| RACGAP1 | DNM3 | RRP7A | PUM1 | SPTAN1 |
| ANXA4 | NOL7 | RSAD1 | EIF3E | POP7 |
| CHD1 | KCNH7 | LLGL1 | NUP188 | TUBB6 |
| ALDH1A1 | PRDX4 | NDUFS5 | RPS12 | CCT8 |
| PAXBP1 | PICALM | ABLIM3 | NSD3 | MPP7 |
| RPRD2 | ESF1 | PATZ1 | INTS14 | ARL1 |
| ZIC2 | CEP131 | MYC | NELFCD | CLPX |
| IDH3A | NELFB | ZMYND11 | SNRPF | SMC4 |
| NYNRIN | IDH3A | ZNF644 | NOP14 | RALA |
| SETD1A | MT-ATP6 | RAP2A | YME1L1 | EXOSC6 |
| HDGFL2 | SETX | PNO1 | SRP68 | SMARCA1 |
| CHAF1B | VAPA | NOMO2 | DOCK7 | ZC3H4 |
| MOGS | SAP130 | TAF4B | MRPL9 | MRPS23 |
| WDR75 | RPRD1B | MED23 | PHF6 | DHX8 |
| HSD17B12 | LIN7C | SPTY2D1 | ITGB1 | FARSA |
| HARS1 | MRPL4 | RPA3 | SP3 | TMEM209 |
| KRT4 | DHCR7 | PRKAA1 | SEC22B | NCOA5 |
| CIRBP | EPHA2 | KAT8 | SREK1 | NKAP |
| CCDC137 | NCLN | SP1 | PML | PSMB5 |
| RAB8B | DYNLL2 | IDH3A | GNAI3 | AKAP2 |
| MRPL43 | SLX9 | FAM162A | PDCD6IP | GPX8 |
| CPT1A | TPX2 | CHTF18 | TK1 | TAF7 |
| CLINT1 | TMEM43 | PIAS1 | PSMD13 | RPP38 |
| PSMD11 | ACTR5 | MTF2 | CLP1 | CTBP1 |
| PSMC3 | MAK16 | MSL2 | AP3B1 | RNF2 |
| TRIM25 | PAXBP1 | ABCE1 | SPATA5L1 | INO80B |
| CETN2 | BZW2 | SEC22B | NDUFS5 | ZKSCAN8 |
| ZNF512B | TUBA4A | ZNF787 | NRF1 |  |
| METAP2 | NACC1 | KDM1B | NOL11 |  |
| VAT1 | NUP50 | ZNF687 | SRPRA |  |
| SPCS3 | MRPL22 | NELFCD | RPF1 |  |
| TWNK | RPS6KA4 | HSPA12A | DDB2 |  |
| RAB10 | EIF3G | SETD1A | ITGB4 |  |
| E2F3 | CTSD | PLD3 | TBPL2 |  |
| PFKM | SLC16A3 | DCUN1D4 | MED6 |  |
| KIF23 | NCBP3 | PAICS | SLX9 |  |
| BBX | RSL24D1 | CDC23 | RAP1A |  |
| BRD4 | GNAI3 | CDCA5 | MCM2 |  |
| PSMD4 | CHD9 | KRT78 | H2AC20 |  |
| CXXC1 | CLPX | PSMA5 | PFDN2 |  |
| DNAJA3 | MED23 | ITGA3 | WDR55 |  |
| TK1 | SMARCD1 | G6PD | EIF2AK2 |  |
| MRPL23 | MRPL41 | HBS1L | PAXBP1 |  |
| SEC23B | ZGPAT | GFPT1 | DR1 |  |
| RAB8A | ITGA3 | EXOSC4 | NCBP3 |  |
| RUNX3 | PKP1 | FKBP2 | LAMP2 |  |
| NDUFB5 | MACROD1 | BAIAP2L1 | USP14 |  |
| SERPINC1 | RPF1 | CKAP5 | VRK2 |  |
| NUP188 | STAG1 | AK2 | EDF1 |  |
| MINK1 | INTS14 | ILKAP | USP10 |  |
| YTHDF3 | ABCE1 | PSMD4 | ADD1 |  |
| ERCC3 | CSNK2A1 | PRKCSH | AGK |  |
| ABCE1 | NELFCD | SRP9 | G3BP2 |  |
| LUC7L3 | HIRA | RCN2 | RPLP2 |  |
| CLASP1 | ADD3 | SUMO1 | ABLIM1 |  |
| VPS4B | CPNE7 | NTPCR | IGF2BP3 |  |
| PSMA2 | CP | FARSB | RCN2 |  |
| MAP4K4 | TIGD2 | RBBP5 | TUBA4A |  |
| ANP32A | HNRNPLL | MRPL23 | BUD13 |  |
| GLE1 | NIFK | MRPS14 | HACD3 |  |
| YWHAH | RCOR1 | BUD31 | ERAL1 |  |
| VRK2 | ACAT2 | PRKACA | FAM162A |  |
| METTL15 | ALDH1A1 | DSTN | IWS1 |  |
| DPM1 | NCOR2 | AZGP1 | CPNE7 |  |
| PIP | CHTF18 | ITGB1 | HDGFL2 |  |
| ATAD1 | RECQL | PSMD11 | CTSZ |  |
| RPS19BP1 | THOC6 | RAB10 | CCNK |  |
| NIPSNAP2 | ABT1 | BZW1 | TXNDC5 |  |
| MRPL48 | EIF3E | RAB35 | SGPL1 |  |
| GLOD4 | EED | MKRN2 | NFRKB |  |
| RBPJ | MCCC1 | EXOSC8 | PRPF4B |  |
| WAPL | TRIM33 | GLE1 | NUP88 |  |
| SRM | MTCH1 | RAB2B | PPIL4 |  |
| ZNF609 | GFPT1 | STT3A | MCCC1 |  |
| PRKACA | ACAD9 | PCNP | LEO1 |  |
| GTF2B | TMCC3 | HIP1R | PSMC1 |  |
| MAZ | PSMD12 | ACTR5 | UTP20 |  |
| UBE2NL | MYO6 | TUBB6 | NOP16 |  |
| LLPH | UBE2E2 | ARL6IP4 | RPS19BP1 |  |
| STK39 | RACGAP1 | MBD3 | TMED9 |  |
| MRPL9 | SERPINC1 | RBBP7 | METAP1 |  |
| NOP10 | ZC3H4 | LEMD2 | MRPS5 |  |
| PHF21A | ETFB | NDUFB9 | SRSF4 |  |
| PIAS4 | PKP4 | PRDX4 | SSR3 |  |
| NIP7 | VPS4B | WDR12 | CKAP5 |  |
| TIA1 | XAB2 | SERPINC1 | FOXJ3 |  |
| TMA16 | NIPSNAP2 | NOP53 | RECQL |  |
| VPS72 | GLUD1 | PRORP | FLOT2 |  |
| C1orf174 | NT5E | MED16 | CASZ1 |  |
| NOL10 | THOC5 | UGDH | EED |  |
| CCNK | INTS2 | USF1 | IARS1 |  |
| ERCC2 | CTPS1 | NUDT16L1 | CHMP1A |  |
| TXN | CCDC137 | CAAP1 | MRPL20 |  |
| ELF3 | INTS7 | DIS3 | RO60 |  |
| ATP1B3 | PURA | ANAPC1 | MRPL43 |  |
| PSMD8 | YBX3 | TUBA1C | CXXC1 |  |
| MAPT | GTF2F1 | ANAPC4 | PPP1R12A |  |
| AKAP17A | TAF6 | CSTF2 | ABCE1 |  |
| YWHAG | SMARCAL1 | RDH13 | NGDN |  |
| BAZ2B | DDB2 | SRM | LAS1L |  |
| MKRN2 | MRPL9 | SDHA | NFIX |  |
| RING1 | PML | NGDN | ZIC2 |  |
| GDAP1 | MRPL18 | KDM2A | RBMXL1 |  |
| PPWD1 | NARS1 | WBP4 | POLR1C |  |
| SPATA5L1 | USP3 | COX15 | BCL7C |  |
| AFF1 | SGF29 | MRPL27 | NUP50 |  |
| RBM22 | RBMS1 | FARSA | NUCKS1 |  |
| IGF2BP3 | AQR | PLOD1 | HLA-B |  |
| PAICS | NIP7 | VPS35 | GOT1 |  |
| CEP131 | SREK1 | RFX1 | TRIM25 |  |
| ZC3HAV1 | MBD2 | SETX | LIN7C |  |
| RRP7A | SLC25A12 | ETFA | APMAP |  |
| NUP88 | CKMT1A | METTL15 | CIRBP |  |
| ETV6 | C17orf80 | CBX1 | NARS1 |  |
| CHTF18 | RAD23B | MRPL15 | RRP15 |  |
| DDX55 | STK39 | YME1L1 | POP7 |  |
| RECQL | ZC3HC1 | P3H4 | SLC16A3 |  |
| INO80C | ZNF462 | CAT | CNN2 |  |
| ITGB4 | MRPL16 | PHF3 | MTHFD2 |  |
| LONP1 | AZGP1 | NUP188 | YBX3 |  |
| EXOSC6 | LAMP1 | MAP2K3 | PHF21A |  |
| APMAP | ANP32B | TECR | BZW2 |  |
| DDX10 | E2F3 | THOC6 | BTAF1 |  |
| MRPS27 | USP10 | MAPRE1 | SEC13 |  |
| PCF11 | SLFN5 | RPLP2 | BMS1 |  |
| PHF23 | ANP32A | IARS1 | ZC3HC1 |  |
| HSD17B11 | RALB | LAS1L | TECR |  |
| MEF2D | POLR2H | ERAL1 | SMN1 |  |
| USP3 | ZNF644 | POLR1A | CACYBP |  |
| IARS1 | ALDH2 | MARS1 | URB1 |  |
| IWS1 | NCBP2 | FOSL2 | MRPL54 |  |
| LAMP2 | RCN2 | ZGPAT | AFF1 |  |
| LEMD2 | ARF5 | JRKL | CRABP2 |  |
| SRPRA | METTL15 | PSMD14 | FKBP2 |  |
| MRPS14 | TMED10 | RPL26L1 | ARL1 |  |
| CHCHD6 | RNF20 | SLC9A3R1 | RNF20 |  |
| C17orf80 | MAPRE1 | KIF4B | UQCRQ |  |
| PRPF4B | CIRBP | CCDC137 | DNAJA2 |  |
| VPS35 | PHLDA2 | TAF3 | YTHDF3 |  |
| GALE | RAI1 | NARS1 | CTBP1 |  |
| NMNAT1 | CMSS1 | ETFB | DHX33 |  |
| UCHL5 | MRPS18A | CAPZA2 | WBP11 |  |
| ERI1 | APMAP | SMPDL3B | MRPS27 |  |
| P3H4 | PHF21A | LARP4B | CDYL |  |
| RRP36 | TMEM109 | WRNIP1 | MCCC2 |  |
| FKBP8 | PSMA2 | APMAP | EXOSC3 |  |
| SEC24B | BCCIP | BRD4 | TBL2 |  |
| RRP15 | ACTR1A | MRPL43 | AQR |  |
| PACSIN3 | DDX55 | SERPINB3 | VPS4B |  |
| NDUFS5 | TLN1 | NACC1 | SRCAP |  |
| CHD6 | FKBP8 | PCID2 | PSMC6 |  |
| DNAJB11 | CNOT1 | HLA-A | YWHAG |  |
| ZNF148 | EXOSC3 | ZKSCAN1 | TOP1MT |  |
| SMC5 | MARS1 | ZNF16 | SLC3A2 |  |
| USP5 | PFKM | NFAT5 | LUC7L |  |
| YBX3 | PSMD1 | BTAF1 | NUDT5 |  |
| CISD1 | CBLL1 | NPM3 | PHLDA2 |  |
| DYNLL2 | RING1 | S100A13 | ATP5MK |  |
| OGDH | LONP1 | LETM1 | UCHL5 |  |
| CHD5 | RPS12 | PPIL4 | RALB |  |
| SFMBT1 | ME1 | PHF21A | CMSS1 |  |
| MCRS1 | RRP36 | THOC1 | CAD |  |
| RIF1 | CASP14 | RDX | DSTN |  |
| EPHA2 | ZCCHC8 | TGM3 | CD59 |  |
| TIGD2 | KRT4 | MED12 | USP6NL |  |
| CRABP2 | KAT7 | PURA | LARP7 |  |
| ATP5F1EP2 | AJUBA | RIOX2 | WDR46 |  |
| SP1 | NXF1 | UPF1 | KNOP1 |  |
| TBC1D10B | MRPS2 | ATXN2L | KRT23 |  |
| FNBP4 | NR2F1 | BYSL | FRG1 |  |
| UTP25 | ILK | POU2F1 | DNAJC11 |  |
| POU2F1 | TRIM4 | NDUFB3 | PSMD14 |  |
| ALKBH5 | HSD17B11 | SMC5 | ABCF2 |  |
| MPHOSPH10 | CKAP5 | RARS1 | SUN1 |  |
| PIAS1 | ZBTB43 | MRPS5 | CAMK2D |  |
| LSM12 | PLEKHA5 | ATAD3B | MTMR2 |  |
| VASP | EMILIN1 | MSL1 | APEH |  |
| USP14 | CCNK | MED27 | MVD |  |
| ENTR1 | SAP18 | NCCRP1 | MOV10 |  |
| SNTB2 | EZH2 | NDUFA9 | ZCCHC8 |  |
| MAVS | BAZ2B | EIF3G | BUD31 |  |
| AQR | PSMA1 | C5orf24 | MRPL3 |  |
| RNF20 | VPS72 | PLCB3 | PLD3 |  |
| TMEM33 | ANXA4 | CWF19L1 | QARS1 |  |
| ZCCHC8 | HOXB4 | EYA3 | TMCC3 |  |
| FLG2 | MRPS21 | MRPL48 | AHDC1 |  |
| IMP3 | DOCK7 | DBNL | CLPX |  |
| POP7 | SMC2 | MED1 | POLR1B |  |
| URB1 | MTMR2 | PSMD7 | HSD17B11 |  |
| CPNE1 | MRM2 | MARCKSL1 | PPP2CB |  |
| LARP4 | VAPB | YWHAG | TAF6 |  |
| CBFB | POP7 | NUP58 | TMEM109 |  |
| EIF3L | SNRPF | UTP23 | GLYR1 |  |
| RCN2 | CDCA5 | SPATS2L | MRPS18A |  |
| ATP6V1A | TUBB6 | UTP20 | DNMBP |  |
| PYGL | CSDE1 | APOO | SLFN5 |  |
| TEX10 | TCEA1 | PPP3CB | UBE2E2 |  |
| RPLP2 | RRP15 | UTP11 | LLGL1 |  |
| IRF2BP1 | UACA | LIN7C | ATP6V1A |  |
| PSMD12 | RUNX3 | BCKDHA | RBMS1 |  |
| NGDN | BPTF | MPND | AK2 |  |
| KLF16 | SDHB | TJP2 | SMARCAL1 |  |
| STRBP | IRF2BPL | HSD17B10 | PSMD7 |  |
| MRPL24 | EIF1AX | STRBP | NIFK |  |
| ABT1 | TUBB4A | KRT80 | ETFA |  |
| NOL11 | PDIA5 | NAA15 | ZNF768 |  |
| RARS1 | CSTA | PRRC2C | DLAT |  |
| MSL2 | ARL1 | CNDP2 | MBNL1 |  |
| GNAI3 | NMNAT1 | SLC16A1 | LLPH |  |
| NOP14 | JUN | ZKSCAN4 | CD9 |  |
| TMCC3 | MTHFD1L | NKAP | HIRA |  |
| LYZ | NUDT5 | PCCA | E2F3 |  |
| PLP2 | SLC16A1 | KRT9 | PSMC4 |  |
| KLF5 | CISD1 | H1-3 | KLF16 |  |
| CD9 | DHCR24 | SYNCRIP | HSPA12A |  |
| CTPS1 | MOV10 | PDCD11 | CLIC4 |  |
| USP6NL | TSFM | U2SURP | MDH1 |  |
| ATIC | CRABP2 | CHD2 | MRPS34 |  |
| MAL2 | MRPL15 | SRRM2 | C14orf93 |  |
| ACAT2 | MRPL20 | PRSS1 | SORD |  |
| FAM169A | WDR76 | RPL17 | PRSS3 |  |
| NOL7 | SFN | RFC2 | MBD3 |  |
| PPP1R8 | AIFM2 | NCOA5 | SERPINA12 |  |
| FKBP2 | LLGL1 | SPTAN1 | FAM169A |  |
| RBBP7 | KTN1 | LDHA | FOSL1 |  |
| CAT | PRPF4B | TUBB2A | JUND |  |
| THOC1 | FBXO11 | XPC | PICALM |  |
| RBM34 | SRPRA | IQGAP1 | SPTY2D1 |  |
| ERP29 | PSME3 | CDC5L | CEP131 |  |
| GTF2H4 | DENR | KHDRBS3 | IMP4 |  |
| INTS2 | RPP30 | FUBP1 | PLEKHA5 |  |
| BUD31 | NUCKS1 | DEK | PPIG |  |
| RALA | CHD1 | RBM15 | RPP30 |  |
| CREB1 | PIP | GAR1 | MPHOSPH8 |  |
| MAFK | CHD5 | KRI1 | CAV3 |  |
| TMX1 | RBM22 | APEX1 | ERCC3 |  |
| NPM3 | TMEM33 | VDAC3 | KIF23 |  |
| EIF1AX | FAM162A | RPL19 | IL18 |  |
| FAM162A | NOP10 | DDX24 | OGDHL |  |
| UBP1 | FOSL1 | ALDH1A3 | RAC2 |  |
| MRPS2 | STAU1 | KRT17 | MED23 |  |
| RALB | SPATA5 | RPS27 | GGH |  |
| SLC9A3R1 | PTGES2 | PSPC1 | INTS5 |  |
| PLEKHA5 | CTDSPL2 | RRBP1 | ALKBH5 |  |
| SCAF8 | RARS1 | SKI | LACRT |  |
| RHOF | TAF4 | BSG | MRPS14 |  |
| PPP2CB | ZNF768 | SMARCB1 | WAPL |  |
| CPNE2 | HLA-A | ACAT1 | PTPN11 |  |
| LUC7L | ZBTB7B | ANXA5 | PNP |  |
| ARL8B | HSD17B12 | DNMT1 | DMKN |  |
| COTL1 | VIRMA | RAB5C | FARSA |  |
| ARF5 | APEH | SLC25A11 | CSNK2A2 |  |
| MYH14 | SEC23B | ZBTB43 | COX15 |  |
| TGM3 | SYNGR2 | CDH3 | CYRIB |  |
| OLA1 | CAD | MFAP1 | PSMB2 |  |
| RNF2 | ZNF48 | CYB5R3 | ERCC2 |  |
| ZFP91 | THAP11 | NR2C2 | SLC25A4 |  |
| SRSF4 | RTCA | CARD10 | PDCD10 |  |
| C19orf53 | TPM1 | CPSF4 | TMEM209 |  |
| WDR76 | PSMD7 | MCM3 | TLN1 |  |
| TMEM109 | ZNF362 | KPNA1 | MRPS12 |  |
| UTP4 | ZBTB7A | MCM6 | SELENOH |  |
| ZC3H13 | ARHGAP35 | TPM4 | ACTR8 |  |
| CDC23 | ZRANB2 | SHMT2 | SP1 |  |
| CSDE1 | SRSF4 | SAMM50 | MED16 |  |
| TUBA1C | DNAJA2 | PDS5B | MT-CO1 |  |
| MRPL47 | CDC42BPB | UBA1 | TMED7 |  |
| NACC1 | MKRN2 | ZNF185 | KAT5 |  |
| BCAS2 | LRPAP1 | PCF11 | RRP7A |  |
| MRPL58 | XPO4 | UFD1 | UBE2L5 |  |
| ERH | PARD3 | WTAP | DNAJB11 |  |
| EXOSC9 | ARHGDIA | ANXA11 | SLC38A2 |  |
| ZBTB7B | LIMS1 | RANBP1 | PRRC2A |  |
| CTSD | PRSS3 | NDUFS7 | EPPK1 |  |
| PKP4 | PCNP | NDUFB5 | TPD52L2 |  |
| DSTN | BMS1 | SP3 | MED1 |  |
| HOXA9 | MAOB | ERI1 | ATXN2L |  |
| MRPS28 | PHC3 | KATNAL2 | SLC9A3R1 |  |
| S100A9 | PSMD14 | DAPK3 | EPB41L2 |  |
| PPME1 | TUBA1C | RBMS1 | ARCN1 |  |
| DCAF13 | PDHA1 | EED | THAP11 |  |
| PRDX4 | DUSP11 | FKBP4 | MPHOSPH10 |  |
| UGDH | CSNK2A2 | ARPC1B | TAF9B |  |
| LLGL1 | IARS1 | DHCR7 | RNH1 |  |
| MSANTD4 | MAFF | LARP7 | EIF3G |  |
| CWF19L1 | PLP2 | ARPC5L | CHRAC1 |  |
| H2AC20 | SP1 | HSD17B12 | MRPL15 |  |
| CBX4 | ISYNA1 | KIF23 | INTS2 |  |
| ABLIM3 | TUBB3 | IWS1 | EXOSC8 |  |
| MTMR2 | FARSB | PAXBP1 | DLG1 |  |
| TIMM50 | COPB2 | RHOT1 | NUMB |  |
| MACF1 | HSPA12A | SNRPB2 | PURA |  |
| JUN | ESPN | PICALM | CGGBP1 |  |
| ZC3HC1 | PRKACA | PDP1 | SAP130 |  |
| CEBPG | TIMM50 | MRPS22 | EXOSC9 |  |
| NIBAN2 | GNA13 | TOE1 | BAIAP2L1 |  |
| CAD | NDUFA2 | CYB5R1 | PPIH |  |
| PLCD3 | RPA3 | SART3 | NT5E |  |
| PRRX2 | EIF5B | AFF4 | TFDP1 |  |
| UBE2E2 | SCO2 | TMED7 | PHC3 |  |
| ZRANB2 | WDR55 | PDHA1 | ARL6IP5 |  |
| MRPL2 | IFITM3 | AURKB | CPNE1 |  |
| KAT8 | MEF2D | FBXO11 | SF3B4 |  |
| PHC3 | PTPN11 | NT5DC2 | MYH14 |  |
| PITX2 | MYOF | GIPC1 | ITGA6 |  |
| MRM2 | MRPS14 | CEP170 | ILK |  |
| EBP | ZFP91 | ZNF319 | TRIM33 |  |
| ALDH7A1 | ANP32E | MCCC2 | IGKC |  |
| DNAJC7 | BUD13 | FOSL1 | ZMYND8 |  |
| CSTF2 | VAT1 | ZC3H13 | IDH3A |  |
| STAG2 | SGPL1 |  | PHF23 |  |
| SLC38A2 | HDAC6 |  | MAOB |  |
| TUBB2A | DCUN1D5 |  | CSNK1A1 |  |
| DNAJC11 | MRPL23 |  | CDK8 |  |
| MTAP | YME1L1 |  | RBBP5 |  |
| SP3 | MED16 |  | ATIC |  |
| IMP4 | GOLGA2 |  | SLC9A3R2 |  |
| MRPL4 | SERPINB3 |  | CDC42BPB |  |
| PDCD10 | STAG2 |  | HAL |  |
| INTS8 | ASNS |  | METTL15 |  |
| NUDT5 | PATZ1 |  | ESYT2 |  |
| MYOF | TMED7 |  | AFF4 |  |
| ABI1 | LSM6 |  | CBLL1 |  |
| TFDP1 | CBFB |  | GM2A |  |
| INTS14 | SLC38A2 |  | KDM2A |  |
| CHMP2B | GLE1 |  | ONECUT2 |  |
| ANP32E | CHAF1B |  | TPM1 |  |
| VIRMA | PPIF |  | HBS1L |  |
| ZNF362 | CSTF2 |  | GLOD4 |  |
| TAF9B | ARCN1 |  | ANAPC4 |  |
| RBMX2 | KNOP1 |  | URB2 |  |
| MRPL27 | RHEB |  | MARCKSL1 |  |
| TRIM33 | BCL7C |  | NAMPT |  |
| CASK | CDX2 |  | ZNF787 |  |
| APEH | PPFIA1 |  | RBM47 |  |
| TMED7 | DNAJB11 |  | ZRANB2 |  |
| MRPS12 | MRPL17 |  | ATP1B1 |  |
| HACD3 | SDHA |  | CLASP1 |  |
| ESYT2 | PPME1 |  | CYB5R1 |  |
| ACAD9 | TMEM209 |  | PTCD3 |  |
| IRF2BPL | GGCT |  | ZFP91 |  |
| PLD3 | ULK1 |  | ACAD9 |  |
| SELENOH | C1D |  | SMC5 |  |
| MYC | TST |  | SCAI |  |
| CACYBP | MSANTD4 |  | VAMP3 |  |
| NDUFA9 | SETD1A |  | EIF3H |  |
| RPA3 | SMPDL3B |  | DNAJC7 |  |
| GOLGA2 | USP5 |  | CREB1 |  |
| ZMYND8 | TSN |  | GIPC1 |  |
| CTR9 | RBM47 |  | ME1 |  |
| MRPL17 | ITGB4 |  | VIRMA |  |
| SRP9 | PTRH2 |  | UACA |  |
| EXOSC8 | RBMS2 |  | MAFK |  |
| SAP30BP | UGDH |  | CDC73 |  |
| EIF2AK2 | LEO1 |  | OXSR1 |  |
| CAPZA2 | YWHAG |  | ERC1 |  |
| CUL4B | BIN3 |  | DSC2 |  |
| PCNP | MRPL43 |  | NOM1 |  |
| ZNF787 | WDR74 |  | TUBB6 |  |
| LEO1 | DNM1L |  | PSMB3 |  |
| PICALM | ABI1 |  | NR2F6 |  |
| MCCC1 | EIF3I |  | VAPB |  |
| SERPINB3 | ALDH7A1 |  | THOC2 |  |
| ZBTB7A | CLOCK |  | SPATA5 |  |
| SAP130 | RIOX2 |  | TWF2 |  |
| RBM47 | DNAJC19 |  | GALE |  |
| EIF3I | PYGB |  | GNA13 |  |
| SENP3 | MRPL27 |  | DHX8 |  |
| INTS4 | PNKP |  | TST |  |
| MARK2 | NOL11 |  | INTS4 |  |
| RBBP5 | SELENOH |  | SP100 |  |
| THOC5 | ANAPC2 |  | SSU72 |  |
| MRPL14 | CAMK2D |  | POU2F1 |  |
| DDX6 | ZNF787 |  | MRPL24 |  |
| ERC1 | ERC1 |  | TMED10 |  |
| MRPL15 | DBNL |  | VWA8 |  |
| MRPS26 | NGDN |  | ANAPC2 |  |
| SPATA5 | CAPZA2 |  | DBNL |  |
| ARL6IP4 | CLIC4 |  | RARS1 |  |
| YME1L1 | P3H4 |  | LTF |  |
| SRP68 | RMDN3 |  | ATP6V0D1 |  |
| RDH13 | POU2F1 |  | DCUN1D4 |  |
| SLBP | TSPYL1 |  | SERPINB1 |  |
| MRPS10 | EIF4G1 |  | PTBP3 |  |
| ARMC10 | HSPE1 |  | LSM12 |  |
| SFN | ARVCF |  | STK24 |  |
| SLC3A2 | AK2 |  | GLE1 |  |
| BIN3 | RBMXL1 |  | PHF8 |  |
| FRG1 | PPP1R12A |  | THOC1 |  |
| MARS1 | KAT8 |  | SERPINB6 |  |
| APOO | BCKDK |  | RUNX1 |  |
| ZC3H15 | DHX16 |  | MCU |  |
| TAF3 | IMP4 |  | APOO |  |
| PSME3 | URB2 |  | MKRN2 |  |
| NSMCE3 | PCF11 |  | PCF11 |  |
| KTN1 | BRD1 |  | NELFE |  |
| EP400 | TAGLN2 |  | SNTB2 |  |
| TMED10 | TAF2 |  | TMEM11 |  |
| MLEC | POR |  | PIN4 |  |
| ACP1 | SNIP1 |  | DTD1 |  |
| PSMD13 | PSMB6 |  | BYSL |  |
| PFDN2 | ERP29 |  | PPA1 |  |
| MOV10 | NR2F6 |  | SPIN1 |  |
| ATP13A1 | OXSR1 |  | CPA4 |  |
| ARVCF | POLR1B |  | AGFG1 |  |
| TMEM209 | CDK8 |  | FGD6 |  |
| BCL7C | YTHDF3 |  | PRRX2 |  |
| NOL9 | THOC1 |  | ABLIM3 |  |
| METAP1 | S100A13 |  | ARID2 |  |
| MSL1 | CPNE1 |  | STRBP |  |
| PDHA1 | POLR1F |  | GOLGA2 |  |
| RPF1 | LEMD2 |  | PARD3 |  |
| ACTR1A | ANAPC4 |  | PTGES2 |  |
| GAL3ST4 | NPM3 |  | SYF2 |  |
| KAT7 | PIK3C2A |  | MEF2D |  |
| TAF2 | EIF2B4 |  | RHOF |  |
| MDN1 | KRT80 |  | GDAP1 |  |
| CYB5R1 | NUP214 |  | POLR2C |  |
| CLOCK | RNH1 |  | COPB1 |  |
| GPKOW | SLC12A2 |  | PSMC3 |  |
| LANCL1 | JRKL |  | CCDC12 |  |
| PAPSS1 | RHOF |  | ATAD1 |  |
| TST | GULP1 |  | UTP23 |  |
| CDK8 | QPCTL |  | SCAF8 |  |
| DNAJC9 | PPP1R18 |  | XAB2 |  |
| PSMC6 | EPB41L2 |  | SYTL1 |  |
| ACTR8 | DSC2 |  | MRPL58 |  |
| SLC12A2 | MARCKSL1 |  | MRPL48 |  |
| DSC2 | GIPC1 |  | TUBA1C |  |
| ESYT1 | SP3 |  | SETX |  |
| WBP11 | LANCL1 |  | GSDMA |  |
| PCGF2 | FRG1 |  | ISY1 |  |
| SUGP1 | GDAP1 |  | MELTF |  |
| NUP214 | IDH3G |  | CTDSPL2 |  |
| MEX3D | PSAT1 |  | TENT4B |  |
| SYF2 | SLC3A2 |  | MSL1 |  |
| CEP170 | NPLOC4 |  | BPTF |  |
| KDM2A | ARL6IP5 |  | FLII |  |
| WRNIP1 | WBP4 |  | PKP4 |  |
| CBLL1 | MRPS26 |  | TAF2 |  |
| CORO1A | RPP38 |  | ZNF362 |  |
| DSC1 | LARS1 |  | CDCA5 |  |
| PRRC2A | TRAM1 |  | GTF2B |  |
| CTDSPL2 | BRD4 |  | KLK5 |  |
| ACOT13 | OGT |  | EIF4B |  |
| ARID2 | CACTIN |  | LAP3 |  |
| CGGBP1 | NOL9 |  | FOSL2 |  |
| SERPINB1 | MEX3D |  | INTS7 |  |
| EIF6 | GLOD4 |  | ITGA3 |  |
| ZNF48 | ESYT1 |  | ATP6V1G1 |  |
| TNPO3 | KIF4B |  | SNX2 |  |
| EIF4B | POLR2C |  | SLC12A2 |  |
| COPB1 | ARL6IP6 |  | EIF3F |  |
| BTAF1 | EEF1B2 |  | TBC1D24 |  |
| RTRAF | RHOT2 |  | ZZZ3 |  |
| MED12 | OAS3 |  | PTMA |  |
| SPCS1 | RAD23A |  | CCDC71L |  |
| WBP4 | MAX |  | ZNF462 |  |
| MLF2 | LAS1L |  | MED12 |  |
| AURKB | PRRX2 |  | HGS |  |
| CENPV | SEC23A |  | P3H4 |  |
| MMS19 | UTP25 |  | ABCD3 |  |
| FAM120A | TNPO3 |  | ZGPAT |  |
| ZCCHC9 | ZC3H13 |  | MGA |  |
| ATP6V1B2 | PDCD6IP |  | NDRG1 |  |
| MARCKSL1 | CYP51A1 |  | SMAD4 |  |
| ARL6IP5 | ARMC10 |  | TRAM1 |  |
| CSRP2 | AIMP1 |  | EPB41 |  |
| RNH1 | GALE |  | TSN |  |
| LYN | LSM12 |  | MCRS1 |  |
| UBE2L3 | SFMBT1 |  | IVD |  |
| DNMBP | FAM169A |  | SARS2 |  |
| ILK | EIF3L |  | RRAS2 |  |
| PIN4 | MGA |  | PMPCA |  |
| ORC1 | EMC2 |  | SLC25A13 |  |
| MRPL20 | TFB2M |  | SRC |  |
| EPB41L2 | CEP170 |  | USP48 |  |
| MRRF | WDR46 |  | KPNA4 |  |
| ALDH2 | CCDC12 |  | SLC16A1 |  |
| SCARB1 | ELF1 |  | MRPL18 |  |
| GDI1 | SUGT1 |  | ALDH2 |  |
| RAB5A | BRMS1 |  | EFHD2 |  |
| PTGES3 | ZC3HAV1 |  | IGF2R |  |
| POLR1C | IGF2BP3 |  | SNX3 |  |
| DNAJC19 | GABPA |  | CUSTOS |  |
| LCOR | MRPL48 |  | CAPNS1 |  |
| NOSIP | ATP6V1B2 |  | GFPT1 |  |
| SPATS2L | ZKSCAN1 |  | ABI2 |  |
| KAT2A | NCCRP1 |  | RIF1 |  |
| UBE2Q1 | GLS |  | FYTTD1 |  |
| NXF1 | MED12 |  | GTPBP6 |  |
| SEPTIN8 | ERCC3 |  | CWF19L1 |  |
| SMAD4 | SLC25A13 |  | KRT77 |  |
| EDF1 | NT5DC2 |  | PRMT5 |  |
| MAX | SNTB2 |  | ZSCAN29 |  |
| EPB41 | ISY1 |  | PRDX5 |  |
| HDAC6 | CEBPG |  | UMPS |  |
| PRCC | KIAA1671 |  | PLOD1 |  |
| ORC3 | RFX5 |  | BCAP31 |  |
| KDM1B | DYNLT2 |  | HAGH |  |
| MED1 | MRPL58 |  | KRT14 |  |
| C7orf50 | SPCS1 |  | DHX9 |  |
| RPP38 | TNKS1BP1 |  | RBM12B |  |
| PPL | TIA1 |  | RAN |  |
| BMP2K | PSMC3 |  | HRNR |  |
| MGA | SYF2 |  | RPS13 |  |
| POR | SCAF1 |  | GNB1 |  |
| NT5E | TM7SF2 |  | KRT74 |  |
| BLVRA | ZMYM3 |  | EIF4A3 |  |
| CACTIN | PALM |  | RPL7A |  |
| NASP | ARID1B |  | HP1BP3 |  |
| SSU72 | DHX8 |  | SNRPD2 |  |
| CDK2 | YARS2 |  | SF3A1 |  |
| PKP2 | SLC9A3R1 |  | H1-0 |  |
| AFF4 | PRRC2A |  | DBT |  |
| RPS21 | MPHOSPH10 |  | GTF2I |  |
| TBC1D24 | BMP2K |  | SNRPD1 |  |
| ATAD3B | SCAF8 |  | PGK1 |  |
| SNX2 | LYPLAL1 |  | TRA2B |  |
| CLK3 | ETFA |  | CDCA2 |  |
| CNOT2 | CLK3 |  | PPP1CA |  |
| CAST | PCCA |  | ACIN1 |  |
| ECI2 | SYNE2 |  | SRSF2 |  |
| SPIN1 | TAF1L |  | SF3A3 |  |
| MRPL22 | MED1 |  | SNRNP40 |  |
| TRAM1 | ABCD3 |  | RFC3 |  |
| TXNRD1 | NOSIP |  | RBM25 |  |
| TBCD | THYN1 |  | VDAC3 |  |
| SRC | PRMT5 |  | ITPRID2 |  |
| ISY1 | ATF2 |  | GOT2 |  |
| ESPN | KMT2A |  | SNRPA |  |
| ZNF280C | SDF4 |  | S100A4 |  |
| JRKL | ZDHHC5 |  | DDX42 |  |
| SCRIB | SUPT7L |  | ABHD10 |  |
| CCNT1 | IVD |  | CTTN |  |
| RNGTT | SLC25A36 |  | APOBEC3C |  |
| ASCC3 | GALK1 |  | P4HA1 |  |
| ATF2 | PLD2 |  | SMARCD2 |  |
| GGH | SMARCAD1 |  | PRPF3 |  |
| TCF25 | SERPINE2 |  | GCN1 |  |
| P3H1 | AHSA1 |  | SMCHD1 |  |
| SHMT1 | FBXO28 |  | EHD2 |  |
| CBR4 | SCYL1 |  | SON |  |
| DLG1 | KLF13 |  | CFL1 |  |
| ELMO3 | SPECC1L |  | EIF2S1 |  |
| KLF12 | WDR6 |  | FTSJ3 |  |
| SMARCAD1 | KRT18 |  | VRK1 |  |
| RSBN1L | H1-0 |  | PDLIM7 |  |
| CCNB2 | RPS4X |  | RREB1 |  |
| ZNF281 | SUPT16H |  | FOXK2 |  |
| SLC16A1 | CNTNAP4 |  | RBM26 |  |
| INO80B | AHCTF1 |  | NOP9 |  |
| UBN2 | GTF2I |  | HSPA4 |  |
| HLA-B | PRPF19 |  | ZNF24 |  |
| ISYNA1 | MYO1C |  | ESRP1 |  |
| PURA | CHERP |  | POP1 |  |
| ABI2 | RPL23 |  | TALDO1 |  |
| PLOD1 | ACIN1 |  | HDAC2 |  |
| ADH5 | SNRNP70 |  | RBM4B |  |
| PREP | RPL18 |  | ZC3H11A |  |
| WDR46 | PPIA |  | PSME3 |  |
| NFATC3 | RBM25 |  | EHMT1 |  |
| ZNF516 | NFIC |  | RHOG |  |
| NUFIP2 | PNN |  | EIF3B |  |
| ZZZ3 | SLC2A1 |  | GNL2 |  |
| LRRC41 | RPS10 |  | PARN |  |
| SOX12 | PPP1R9B |  | TUBB3 |  |
| FAR1 | PFKP |  | ARID1B |  |
| RSBN1 | FEN1 |  | MRPS23 |  |
| BMI1 | XPO1 |  | ARPC1B |  |
| CLTA | GOT2 |  | PGM5 |  |
| PLS1 | RPS17 |  | NOC4L |  |
| SNX1 | NUP160 |  | PELP1 |  |
| SMC4 | DDX18 |  | ASPH |  |
| TNKS1BP1 | TPI1 |  | ABT1 |  |
| ARHGAP18 | ESYT2 |  | MRPS18B |  |
| PLD2 | RNPS1 |  | INTS8 |  |
| ERO1A | SLC25A6 |  | HMG20B |  |
| ACADVL | MTHFD1 |  | WRNIP1 |  |
| KMT2A | RBM8A |  | APOBEC3B |  |
| CLUAP1 | CYB5R3 |  | VEZF1 |  |
| HARS2 | RRS1 |  | NPM3 |  |
| AMOTL1 | RAB7A |  | PHF3 |  |
| MAOB | SNU13 |  | KPNA6 |  |
| PNPT1 | PSMB5 |  | RAB14 |  |
| OSBPL8 | H2AC20 |  | MPP7 |  |
| RRAS | PSMC2 |  | SDHA |  |
| CDKN2AIP | GTF3C3 |  | REEP6 |  |
| LUZP1 | DARS1 |  | MMTAG2 |  |
| GALK1 | SF3B6 |  | RBM33 |  |
| SPECC1L | ANXA3 |  | PPA2 |  |
| HAGH | DDX27 |  | UGDH |  |
| DHX9 | NCBP1 |  | WDR76 |  |
| HP1BP3 | IGF2BP2 |  | TFAP2B |  |
| PDCD11 | ZMYM4 |  | IL36G |  |
| H2BC12L | LENG8 |  | TIMM50 |  |
| MYBBP1A | GIGYF2 |  | MGST1 |  |
| PPP1R12A | RNF40 |  | ADD3 |  |
| SLTM | RRP7A |  | TMEM201 |  |
| KRT14 | IDH1 |  | KAT7 |  |
| LDHA | CLP1 |  | RFX1 |  |
| OAT | ALKBH5 |  | CHMP4B |  |
| TMPO | CYB5B |  | C5orf24 |  |
| MSH6 | EIF6 |  | SEC23B |  |
| UBTF | EMG1 |  | RIOX2 |  |
| PRPF40A | HDGF |  | EIF4G2 |  |
| UQCRC1 | TASOR |  | DDX55 |  |
| CCT6A | DNMBP |  | TMED2 |  |
| RPL5 | TEFM |  | NDUFB9 |  |
| NUP205 | CEBPB |  | CAST |  |
| COX4I1 | MRPS22 |  | SUPT20H |  |
| HELLS | SNRPB2 |  | SYNE2 |  |
| RPL22L1 | STT3B |  | DGCR8 |  |
| VARS1 | NTHL1 |  |  |  |
| RPL12 | NDUFA11 |  |  |  |
| API5 | EPCAM |  |  |  |
| RPL18 | TXN |  |  |  |
| KPNA4 | TOE1 |  |  |  |
| KRT13 | ERAL1 |  |  |  |
| MTA1 | DHX38 |  |  |  |
| NUP107 | SUGP1 |  |  |  |
| PWP2 | RPS19BP1 |  |  |  |
| GOT2 | NUMB |  |  |  |
| PABPN1 | RAB10 |  |  |  |
| STOML2 | CAPN2 |  |  |  |
| GTF3C5 | TRIR |  |  |  |
| ACLY | SMAD4 |  |  |  |
| OXA1L | MSL1 |  |  |  |
| ASPH | MRPL2 |  |  |  |
| ADD1 | EPB41 |  |  |  |
| OCIAD1 | NSUN4 |  |  |  |
| NDUFV1 | MCRS1 |  |  |  |
| YBX1 | ANXA11 |  |  |  |
| TOX4 | MTPAP |  |  |  |
| HMGN1 | KLF16 |  |  |  |
| TRIM72 | PPIL4 |  |  |  |
| PRDX2 | MAP1S |  |  |  |
| LUC7L2 | ENTR1 |  |  |  |
| PMPCB | COPB1 |  |  |  |
| TRIM71 | CPT1A |  |  |  |
| PRPF31 | CHD3 |  |  |  |
| BZW1 | RCOR3 |  |  |  |
| RPL15 | EIF4B |  |  |  |
| TEAD4 | LARP7 |  |  |  |
| GRHL2 | NFIA |  |  |  |
| PLS3 | FER |  |  |  |
| CBX1 | NELFE |  |  |  |
| INTS6 | PRCC |  |  |  |
| GNA11 | ORC1 |  |  |  |
| AGK | ATAD3B |  |  |  |
| RPL36AL | TBCD |  |  |  |
| TFAP2B | USP14 |  |  |  |
| C5orf24 |  |  |  |  |
| GTF2F1 |  |  |  |  |
| C1QBP |  |  |  |  |
| KANK2 |  |  |  |  |
| DNAJA2 |  |  |  |  |
| DYNLL1 |  |  |  |  |
| MT-ATP6 |  |  |  |  |
| RMI1 |  |  |  |  |
| ATP1B1 |  |  |  |  |
| ITGA3 |  |  |  |  |
| ZGPAT |  |  |  |  |
| SSR3 |  |  |  |  |
| AGPAT5 |  |  |  |  |
| PSMC1 |  |  |  |  |
| PSMA5 |  |  |  |  |
| SUPT6H |  |  |  |  |
| UNC45A |  |  |  |  |
| DHX16 |  |  |  |  |
| RBM15B |  |  |  |  |
| NELFCD |  |  |  |  |
| STAU1 |  |  |  |  |
| TENT4B |  |  |  |  |
| ARPC1B |  |  |  |  |
| CMSS1 |  |  |  |  |
| SDHB |  |  |  |  |
| MYO6 |  |  |  |  |
| AK2 |  |  |  |  |
| PSMD14 |  |  |  |  |
| NELFE |  |  |  |  |
| KLF13 |  |  |  |  |
| MEPCE |  |  |  |  |
| RBMS1 |  |  |  |  |
| TCF7L1 |  |  |  |  |
| THAP11 |  |  |  |  |
| NCLN |  |  |  |  |
| BRMS1 |  |  |  |  |
| UACA |  |  |  |  |
| BMS1 |  |  |  |  |
| DDX20 |  |  |  |  |
| SERPINB6 |  |  |  |  |
| TUBB6 |  |  |  |  |
| GIPC1 |  |  |  |  |
| EEF1B2 |  |  |  |  |
| UNG |  |  |  |  |
| BTF3L4 |  |  |  |  |
| COQ8A |  |  |  |  |
| PRKD2 |  |  |  |  |
| BCKDHA |  |  |  |  |
| EGR1 |  |  |  |  |
| TM7SF2 |  |  |  |  |
| MPRIP |  |  |  |  |
| PTGES2 |  |  |  |  |
| NCCRP1 |  |  |  |  |

| **Supplementary Table 2. The proteins pulled down by each of the five fragments of the PTGS2 promotor (F1, F2, F3, F4 and F5)** | | | | |
| --- | --- | --- | --- | --- |
| F1（1-498bp） | F2（499-981bp） | F3（982-1052bp） | F4（1053-1434bp） | F5（1435-2000bp） |
| DTD1 | NDUFB9 | PQBP1 | SIX4 | TOP1MT |
| MED24 | USP48 | FLII | THOC3 | USP34 |
| ATF1 | EVPL | EPPK1 | UCHL5 | HOMEZ |
| CUL3 | THOC3 | STK24 | TAF3 | PPP2CB |
| AARS1 | PKP1 | AGFG1 | SPCS3 | EIF4H |
| MED6 | PPA1 | MRPS25 | G3BP2 | CLINT1 |
| UBE2I | SH3PXD2B | MRPL39 | DSTN | SERPINB1 |
| CUSTOS | MCCC2 | LRRC1 | RFX1 | BCAS2 |
| PRKAA1 | SIX4 | CTNNBL1 | NAP1L1 | ZSCAN29 |
| AP2S1 | UMPS | AP3B1 | MRPS5 | RPS26 |
| TMEM201 | GOT1 | SUMO3 | DDX6 | XP32 |
| L3MBTL2 | SMPDL3B | RAC2 | CAAP1 | DR1 |
| LSM3 | YARS2 | FLOT2 | CDC37 | RAP1A |
| USP34 | SLC25A13 | EIF4G2 | USP48 | VRK2 |
| FYTTD1 | SCAF1 | KRT79 | SRSF11 | MAZ |
| MBOAT7 | LSR | MSX1 | TXNL1 | OGDHL |
| MCTS1 | CAAP1 | KRT84 | APOO | HACD3 |
| ONECUT2 | MVD | JPT2 | TFDP1 | ABLIM3 |
| REEP6 | SUPT20H | TXNDC5 | YWHAE | RAP2A |
| SP100 | CDCA2 | VAMP3 | PC | PRKAA1 |
| RPS26 | SRSF11 | RAB2A | XRCC1 | MSL2 |
| EIF4H | MDH1 | DAZAP1 | CTCF | DCUN1D4 |
| FLNC | SERBP1 | MRPS34 | TARS1 | FKBP2 |
| NDUFA4 | PGAM1 | PRKAR1A | MRTO4 | SRP9 |
| NDUFB3 | SMCHD1 | MRPL54 | SCAI | EXOSC8 |
| PPP3CA | RBM3 | DNM3 | GSTO1 | COX15 |
| TECR | ANXA3 | MRPL18 | CTSZ | PLOD1 |
| IDH2 | PHF6 | BCCIP | PSMA5 | TECR |
| CLINT1 | ILKAP | RPS12 | TRIM25 | NDUFB3 |
| VRK2 | PLCB3 | ME1 | PRKCSH | CWF19L1 |
| MAZ | PSMA6 | PTPN11 | ITGB1 | AFF4 |
| AFF1 | EIF4E | SGPL1 | EIF3CL |  |
| PHF23 | FOSL2 | ARCN1 | ZNF512B |  |
| LAMP2 | TCEA1 | TSN | NOMO2 |  |
| PACSIN3 | TERF2IP | PTRH2 | RBBP5 |  |
| PYGL | MRPL45 | CLIC4 | BUD31 |  |
| MRPL24 | CDC37 | RMDN3 | MBD3 |  |
| MSL2 | EIF3G | RBMXL1 | GTF2B |  |
| CD9 | TBPL2 | NR2F6 | NDUFB9 |  |
| ATIC | GRSF1 | ABCD3 | SUPT20H |  |
| FKBP2 | TBRG4 | PRMT5 | MAT2A |  |
| PPP2CB | FABP5 | IVD | H3-3A |  |
| OLA1 | MRPL41 | SLC25A36 | H2BC12L |  |
| BCAS2 | TLN1 | AHSA1 | ANXA6 |  |
| ERH | TUBA4A |  | GNB1 |  |
| EXOSC9 | COX5B |  | HMGA2 |  |
| CWF19L1 | RFX1 |  | AP2M1 |  |
| ABLIM3 | FAM98A |  | ACTR3 |  |
| DNAJC7 | OXSR1 |  | SPTBN1 |  |
| PDCD10 | LYPLAL1 |  | HMGB3 |  |
| TAF9B | ATP6V1G1 |  | PDHB |  |
| HACD3 | MAT2A |  | CNN2 |  |
| SRP9 | H3-3A |  | SRSF2 |  |
| EXOSC8 | GNB1 |  | SERBP1 |  |
| MARK2 | ANXA6 |  | ANXA5 |  |
| MRPL14 | AP2M1 |  | SLC25A10 |  |
| ZC3H15 | SPTBN1 |  | PDIA3 |  |
| ACP1 | HMGA2 |  | CASZ1 |  |
| METAP1 | ACTR3 |  | COX4I1 |  |
| GPKOW | SRSF2 |  | PFN1 |  |
| CGGBP1 | RREB1 |  | TEAD1 |  |
| SERPINB1 | CNN2 |  | XPC |  |
| RTRAF | NFAT5 |  | RUNX1 |  |
| CSRP2 | HMGB3 |  | MECP2 |  |
| PTGES3 | PFN1 |  | RREB1 |  |
| SEPTIN8 | PDIA3 |  | PA2G4 |  |
| PPL | CASZ1 |  | AHCY |  |
| SSU72 | ANXA5 |  | CCT5 |  |
| AFF4 | AHCY |  | APOBEC3C |  |
| SNX2 | MSN |  | VCP |  |
| CAST | P4HA1 |  | TOMM20 |  |
| SPIN1 | YWHAE |  | S100A4 |  |
| TXNRD1 | PA2G4 |  | PDIA4 |  |
| SRC | ST13 |  | CPNE3 |  |
| GGH | APOBEC3C |  | SET |  |
| DLG1 | XPC |  | HCFC1 |  |
| ELMO3 | SSR1 |  | CALR |  |
| PLOD1 | PC |  | CAPN1 |  |
| ZZZ3 | HCFC1 |  | SHMT2 |  |
| AMOTL1 | POLR1F |  | PDLIM7 |  |
| OSBPL8 | ING4 |  | ATP5F1C |  |
| HAGH | RUNX1 |  | DNMT1 |  |
| DYNLL1 | CYCS |  | ARPC4 |  |
| ATP1B1 | HSD17B10 |  | MSN |  |
| MEPCE | GPI |  | AKAP12 |  |
| SERPINB6 | CPNE3 |  | CORO1C |  |
|  | DNMT1 |  | NFATC2 |  |
|  | CCT5 |  | VRK1 |  |
|  | RAE1 |  | DDX1 |  |
|  | CAPN1 |  | HK1 |  |
|  | AKAP12 |  | EIF2S3 |  |
|  | PDIA4 |  | DLD |  |
|  | EIF2S3 |  | HMCES |  |
|  | EIF5A |  | PGAM1 |  |
|  | MECP2 |  | COX6C |  |
|  | ATP5F1C |  | CDK11B |  |
|  | CALR |  | SSB |  |
|  | WDR1 |  | ABHD10 |  |
|  | PEBP1 |  | RPL32 |  |
|  | NFATC2 |  | CYCS |  |
|  | ABHD10 |  | UBA1 |  |
|  | VCP |  | MYH14 |  |
|  | CORO1C |  | STIP1 |  |
|  | VRK1 |  | WDR1 |  |
|  | SET |  | PLS3 |  |
|  | AP2B1 |  | CDC73 |  |
|  | TEAD1 |  | GPI |  |
|  | ARPC4 |  | HSPA4 |  |
|  | SHMT2 |  | SEPTIN11 |  |
|  | XRCC1 |  | HARS1 |  |
|  | ARPC2 |  | PEBP1 |  |
|  | SEPTIN11 |  | SMCHD1 |  |
|  | CDK11B |  | FDFT1 |  |
|  | COX6C |  | MAFG |  |
|  | DDB2 |  | TRIM72 |  |
|  | MTHFD2 |  | AP2B1 |  |
|  | CTCF |  | ARPC2 |  |
|  | HMCES |  | FAM98A |  |
|  | CDC73 |  | PDP1 |  |
|  | CFL1 |  | TRAP1 |  |
|  | PRDX6 |  | FAU |  |
|  | S100A4 |  | ETF1 |  |
|  | DDX1 |  | RBM3 |  |
|  | NFATC1 |  | DRAP1 |  |
|  | DLD |  | CD44 |  |
|  | CYB5R3 |  | RANBP1 |  |
|  | RANBP1 |  | RAE1 |  |
|  | NPEPPS |  | FKBP4 |  |
|  | AP2A1 |  | TFRC |  |
|  | HK1 |  | CFL1 |  |
|  | VCL |  | EXOSC4 |  |
|  | TRAP1 |  | GART |  |
|  | HSPA4 |  | TRIM41 |  |
|  | FDFT1 |  | BTF3 |  |
|  | SLIRP |  | ARPC1B |  |
|  | SND1 |  | G3BP1 |  |
|  | KPRP |  | ACOT7 |  |
|  | FAU |  | CCAR1 |  |
|  | PSMA1 |  | LAD1 |  |
|  | CBX5 |  | TOMM70 |  |
|  | STIP1 |  | MACROH2A2 |  |
|  | CCAR1 |  | AP2A1 |  |
|  | HDGF |  | SSR1 |  |
|  | CEBPB |  | CBX5 |  |
|  | ETF1 |  | ACTR2 |  |
|  | UBA1 |  | CLIC1 |  |
|  | GLUD1 |  | NFAT5 |  |
|  | SLC25A10 |  | HMGN1 |  |
|  | PDLIM7 |  | ABCF1 |  |
|  | EIF5B |  | MORF4L1 |  |
|  | GTF2F2 |  | SLIRP |  |
|  | GSTO1 |  | GDI2 |  |
|  | MORF4L1 |  | GTF2F2 |  |
|  | CD44 |  | EIF4E |  |
|  | ABCF1 |  | PRDX6 |  |
|  | CNP |  | FABP5 |  |
|  | MPG |  | PAICS |  |
|  | PSMA4 |  | BZW1 |  |
|  | EXOSC4 |  | RGPD3 |  |
|  | MAFG |  | PLIN3 |  |
|  | DIS3 |  | PDIA6 |  |
|  | TRIM41 |  | AGK |  |
|  | TOMM70 |  | RDX |  |
|  | DRAP1 |  | ILKAP |  |
|  | HSPH1 |  | C14orf93 |  |
|  | PLIN3 |  | C5orf24 |  |
|  | DNAJC8 |  | SND1 |  |
|  | FKBP4 |  | PDAP1 |  |
|  | TPM1 |  | EIF4A2 |  |
|  | TARS1 |  | CBX1 |  |
|  | PHF5A |  | MPG |  |
|  | CKAP5 |  | TBRG4 |  |
|  | G3BP1 |  | CSRP1 |  |
|  | PSMC4 |  | CNP |  |
|  | HNRNPLL |  | G6PD |  |
|  | SSB |  | SEC61B |  |
|  | YARS1 |  | FOXJ3 |  |
|  | BTF3 |  | MAFK |  |
|  | CSNK1A1 |  | ZIC2 |  |
|  | GSTP1 |  | PDCD6 |  |
|  | EHF |  | GSTP1 |  |
|  | G6PD |  | SEPTIN10 |  |
|  | EIF3E |  | FOSL2 |  |
|  | ARHGDIA |  | LSM4 |  |
|  | RGPD3 |  | HSD17B10 |  |
|  | CAMK2D |  | TBPL2 |  |
|  | HDLBP |  | LETM1 |  |
|  | LAD1 |  | PSMA4 |  |
|  | MRTO4 |  | GMPS |  |
|  | ZBTB43 |  | PSMA6 |  |
|  | PDHB |  | PLCB3 |  |
|  | SCAI |  | DNAJC8 |  |
|  | SEPTIN10 |  | TWNK |  |
|  | USP7 |  | NDUFA9 |  |
|  | CSRP1 |  | YARS1 |  |
|  | NUDC |  | EPS8 |  |
|  | PDCD6 |  | VCL |  |
|  | PDP1 |  | ATP1B3 |  |
|  | SRCAP |  | CREB1 |  |
|  | GPD2 |  | GPD2 |  |
|  | ITGB1 |  | EHF |  |
|  | PDIA6 |  | DIS3 |  |
|  | LSM4 |  | SORD |  |
|  | GMPS |  | NRF1 |  |
|  | PGM5 |  | PLD3 |  |
|  | FKBP3 |  | MRPL19 |  |
|  | PDAP1 |  | ARPC5 |  |
|  | CAP1 |  | GSR |  |
|  | EPS8 |  | METAP2 |  |
|  | RAD23B |  | POLR1C |  |
|  | TALDO1 |  | VPS35 |  |
|  | TMEM43 |  | RPL15 |  |
|  | DPYSL2 |  | PSME1 |  |
|  | PSMA3 |  | NFATC1 |  |
|  | RPRD1A |  | CAP1 |  |
|  | PDIA5 |  | PGD |  |
|  | EIF4A2 |  | CHAF1A |  |
|  | NCBP3 |  | MRPL45 |  |
|  | DHCR7 |  | TERF2IP |  |
|  | ARPC5 |  | ACADVL |  |
|  | CD109 |  | COX7A2 |  |
|  | TFRC |  | HMG20B |  |
|  | LETM1 |  | TALDO1 |  |
|  | RFX5 |  | HSPH1 |  |
|  | HIRA |  | MCCC2 |  |
|  | PSMB6 |  | NDUFS5 |  |
|  | EIF3CL |  | HDGFL2 |  |
|  | MBD2 |  | CUL1 |  |
|  | CHAF1A |  | SRM |  |
|  | GART |  | FKBP3 |  |
|  | STMN1 |  | PICALM |  |
|  | MRPL16 |  | MT-ATP6 |  |
|  | ZNF644 |  | MRPL4 |  |
|  | FARSB |  | DHCR7 |  |
|  | NRF1 |  | TMEM43 |  |
|  | BZW2 |  | ACTR5 |  |
|  | ADD3 |  | BZW2 |  |
|  | ELF1 |  | TUBA4A |  |
|  | MRPS18A |  | NUP50 |  |
|  | NUCKS1 |  | EIF3G |  |
|  | SLFN5 |  | NCBP3 |  |
|  | SEC61B |  | MED23 |  |
|  | PDCD6IP |  | ITGA3 |  |
|  | MTHFD1L |  | PKP1 |  |
|  | RNF40 |  | MACROD1 |  |
|  | MACROH2A2 |  | ABCE1 |  |
|  | CUL1 |  | HIRA |  |
|  | POLR1B |  | ADD3 |  |
|  | EIF4G1 |  | HNRNPLL |  |
|  | MRPL3 |  | ALDH1A1 |  |
|  | ETFA |  | RECQL |  |
|  | SORD |  | THOC6 |  |
|  | RPL32 |  | EIF3E |  |
|  | WDR55 |  | MCCC1 |  |
|  | FOSL1 |  | TRIM33 |  |
|  | NARS1 |  | PSMD12 |  |
|  | MBD3 |  | SERPINC1 |  |
|  | NDUFA11 |  | ETFB |  |
|  | ACTR2 |  | VPS4B |  |
|  | ANP32B |  | GLUD1 |  |
|  | PSME1 |  | PURA |  |
|  | SNIP1 |  | GTF2F1 |  |
|  | NOMO2 |  | SMARCAL1 |  |
|  | COX7A2 |  | DDB2 |  |
|  | SCO2 |  | NARS1 |  |
|  | GDI2 |  | RBMS1 |  |
|  | PGD |  | MBD2 |  |
|  | STRAP |  | C17orf80 |  |
|  | C14orf93 |  | RAD23B |  |
|  | OGT |  | ZC3HC1 |  |
|  | LTA4H |  | ZNF462 |  |
|  | FOXJ3 |  | MRPL16 |  |
|  | NUP50 |  | ANP32B |  |
|  | PRKCSH |  | E2F3 |  |
|  | NAMPT |  | ZNF644 |  |
|  | HSPE1 |  | ALDH2 |  |
|  | GSR |  | RCN2 |  |
|  | S100A13 |  | CIRBP |  |
|  | MRPL19 |  | RAI1 |  |
|  | ETFB |  | APMAP |  |
|  | CTSZ |  | PHF21A |  |
|  | MED23 |  | PSMA2 |  |
|  | G3BP2 |  | MARS1 |  |
|  | QARS1 |  | MRPS2 |  |
|  | EIF3D |  | CKAP5 |  |
|  | DENR |  | ZBTB43 |  |
|  | CDCA5 |  | EMILIN1 |  |
|  | HMG20B |  | PSMA1 |  |
|  | PSMB3 |  | ANXA4 |  |
|  | THOC6 |  | CDCA5 |  |
|  | SDHA |  | TCEA1 |  |
|  | NAP1L1 |  | JUN |  |
|  | CLIC1 |  | MTHFD1L |  |
|  | MACROD1 |  | NUDT5 |  |
|  | ACTR5 |  | SLC16A1 |  |
|  | ACOT7 |  | TSFM |  |
|  | ZNF462 |  | MRPL15 |  |
|  | ANXA4 |  | WDR76 |  |
|  | ALDH1A1 |  | SRPRA |  |
|  | ZIC2 |  | PSME3 |  |
|  | HDGFL2 |  | DENR |  |
|  | HARS1 |  | NUCKS1 |  |
|  | CIRBP |  | FAM162A |  |
|  | MRPL43 |  | FOSL1 |  |
|  | TRIM25 |  | RARS1 |  |
|  | ZNF512B |  | SRSF4 |  |
|  | METAP2 |  | DNAJA2 |  |
|  | VAT1 |  | PCNP |  |
|  | SPCS3 |  | MAOB |  |
|  | TWNK |  | PDHA1 |  |
|  | E2F3 |  | IARS1 |  |
|  | BRD4 |  | SP1 |  |
|  | MRPL23 |  | FARSB |  |
|  | SERPINC1 |  | HSPA12A |  |
|  | YTHDF3 |  | EIF5B |  |
|  | ERCC3 |  | SCO2 |  |
|  | ABCE1 |  | ZFP91 |  |
|  | CLASP1 |  | VAT1 |  |
|  | VPS4B |  | MRPL23 |  |
|  | PSMA2 |  | CBFB |  |
|  | ANP32A |  | GLE1 |  |
|  | GLE1 |  | CSTF2 |  |
|  | YWHAH |  | DNAJB11 |  |
|  | MRPL48 |  | SDHA |  |
|  | GLOD4 |  | SMPDL3B |  |
|  | SRM |  | UGDH |  |
|  | GTF2B |  | YWHAG |  |
|  | STK39 |  | MRPL43 |  |
|  | PHF21A |  | ALDH7A1 |  |
|  | TIA1 |  | RIOX2 |  |
|  | ATP1B3 |  | ZNF787 |  |
|  | YWHAG |  | POU2F1 |  |
|  | GDAP1 |  | HSPE1 |  |
|  | IGF2BP3 |  | AK2 |  |
|  | PAICS |  | PPP1R12A |  |
|  | RECQL |  | KAT8 |  |
|  | APMAP |  | OXSR1 |  |
|  | MRPS27 |  | POLR1B |  |
|  | MEF2D |  | THOC1 |  |
|  | IARS1 |  | S100A13 |  |
|  | SRPRA |  | POLR1F |  |
|  | C17orf80 |  | NPM3 |  |
|  | VPS35 |  | JRKL |  |
|  | UCHL5 |  | EPB41L2 |  |
|  | NDUFS5 |  | MARCKSL1 |  |
|  | DNAJB11 |  | GIPC1 |  |
|  | USP5 |  | SP3 |  |
|  | CRABP2 |  | BRD4 |  |
|  | SP1 |  | OGT |  |
|  | UTP25 |  | MRPL48 |  |
|  | POU2F1 |  | MED12 |  |
|  | ALKBH5 |  | SLC25A13 |  |
|  | VASP |  | TIA1 |  |
|  | USP14 |  | SLC9A3R1 |  |
|  | RNF20 |  | ETFA |  |
|  | CPNE1 |  | KMT2A |  |
|  | CBFB |  | GOT2 |  |
|  | RCN2 |  | CYB5R3 |  |
|  | ATP6V1A |  | H2AC20 |  |
|  | PSMD12 |  | ANXA3 |  |
|  | KLF16 |  | RNF40 |  |
|  | RARS1 |  | HDGF |  |
|  | ACAT2 |  | CEBPB |  |
|  | FAM169A |  | STT3B |  |
|  | PPP1R8 |  | NDUFA11 |  |
|  | THOC1 |  | SMAD4 |  |
|  | ERP29 |  | KLF16 |  |
|  | BUD31 |  | COPB1 |  |
|  | CREB1 |  |  |  |
|  | MAFK |  |  |  |
|  | NPM3 |  |  |  |
|  | FAM162A |  |  |  |
|  | MRPS2 |  |  |  |
|  | SLC9A3R1 |  |  |  |
|  | COTL1 |  |  |  |
|  | MYH14 |  |  |  |
|  | ZFP91 |  |  |  |
|  | SRSF4 |  |  |  |
|  | WDR76 |  |  |  |
|  | TMEM109 |  |  |  |
|  | CSDE1 |  |  |  |
|  | MRPL58 |  |  |  |
|  | CTSD |  |  |  |
|  | DSTN |  |  |  |
|  | UGDH |  |  |  |
|  | H2AC20 |  |  |  |
|  | JUN |  |  |  |
|  | ZC3HC1 |  |  |  |
|  | PRRX2 |  |  |  |
|  | UBE2E2 |  |  |  |
|  | ZRANB2 |  |  |  |
|  | KAT8 |  |  |  |
|  | ALDH7A1 |  |  |  |
|  | CSTF2 |  |  |  |
|  | DNAJC11 |  |  |  |
|  | SP3 |  |  |  |
|  | MRPL4 |  |  |  |
|  | NUDT5 |  |  |  |
|  | MYOF |  |  |  |
|  | TFDP1 |  |  |  |
|  | ZNF362 |  |  |  |
|  | TRIM33 |  |  |  |
|  | APEH |  |  |  |
|  | MRPS12 |  |  |  |
|  | ACAD9 |  |  |  |
|  | IRF2BPL |  |  |  |
|  | PLD3 |  |  |  |
|  | SELENOH |  |  |  |
|  | CACYBP |  |  |  |
|  | NDUFA9 |  |  |  |
|  | PCNP |  |  |  |
|  | ZNF787 |  |  |  |
|  | PICALM |  |  |  |
|  | MCCC1 |  |  |  |
|  | SAP130 |  |  |  |
|  | INTS4 |  |  |  |
|  | RBBP5 |  |  |  |
|  | DDX6 |  |  |  |
|  | MRPL15 |  |  |  |
|  | SFN |  |  |  |
|  | FRG1 |  |  |  |
|  | MARS1 |  |  |  |
|  | APOO |  |  |  |
|  | TAF3 |  |  |  |
|  | PSME3 |  |  |  |
|  | PFDN2 |  |  |  |
|  | PDHA1 |  |  |  |
|  | TST |  |  |  |
|  | CDK8 |  |  |  |
|  | SLC12A2 |  |  |  |
|  | WBP11 |  |  |  |
|  | NUP214 |  |  |  |
|  | SYF2 |  |  |  |
|  | EIF4B |  |  |  |
|  | COPB1 |  |  |  |
|  | MED12 |  |  |  |
|  | MARCKSL1 |  |  |  |
|  | RNH1 |  |  |  |
|  | ILK |  |  |  |
|  | PIN4 |  |  |  |
|  | MRPL20 |  |  |  |
|  | EPB41L2 |  |  |  |
|  | MRRF |  |  |  |
|  | ALDH2 |  |  |  |
|  | GDI1 |  |  |  |
|  | POLR1C |  |  |  |
|  | NOSIP |  |  |  |
|  | SMAD4 |  |  |  |
|  | EDF1 |  |  |  |
|  | EPB41 |  |  |  |
|  | PRCC |  |  |  |
|  | TBC1D24 |  |  |  |
|  | MRPL22 |  |  |  |
|  | TRAM1 |  |  |  |
|  | JRKL |  |  |  |
|  | SLC16A1 |  |  |  |
|  | HLA-B |  |  |  |
|  | PURA |  |  |  |
|  | PREP |  |  |  |
|  | ACADVL |  |  |  |
|  | KMT2A |  |  |  |
|  | MAOB |  |  |  |
|  | RRAS |  |  |  |
|  | H2BC12L |  |  |  |
|  | PPP1R12A |  |  |  |
|  | COX4I1 |  |  |  |
|  | GOT2 |  |  |  |
|  | HMGN1 |  |  |  |
|  | TRIM72 |  |  |  |
|  | BZW1 |  |  |  |
|  | RPL15 |  |  |  |
|  | PLS3 |  |  |  |
|  | CBX1 |  |  |  |
|  | AGK |  |  |  |
|  | TFAP2B |  |  |  |
|  | C5orf24 |  |  |  |
|  | GTF2F1 |  |  |  |
|  | DNAJA2 |  |  |  |
|  | MT-ATP6 |  |  |  |
|  | ITGA3 |  |  |  |
|  | PSMA5 |  |  |  |
|  | ARPC1B |  |  |  |
|  | SDHB |  |  |  |
|  | AK2 |  |  |  |
|  | NELFE |  |  |  |
|  | RBMS1 |  |  |  |
|  | NCLN |  |  |  |
|  | GIPC1 |  |  |  |
|  | EEF1B2 |  |  |  |
|  | TM7SF2 |  |  |  |
|  | PTGES2 |  |  |  |

| **Supplementary Table 3. The 62 transcription factors in regulating PTGS2 expression based on the screen set threshold of log2FC > 1** | | | | | | | | | | | |
| --- | --- | --- | --- | --- | --- | --- | --- | --- | --- | --- | --- |
| Dataset ID | Target Gene | TF | Knock-Method | Tissue Type | Biosample Name | Profile ID | Platform | Mean Expr. of Control | Mean Expr. of Treat | Fold Change | Log2FC |
| DataSet_01_005 | PTGS2 | POU5F1 | siRNA | Bone_marrow | GBS6 | GSE12320 | GPL570 | 3.76667 | 1.83333 | 0.48673 | -1.03882 |
| DataSet_01_010 | PTGS2 | TP53 | shRNA | Dermal_fibroblasts | iPS cells | GSE13334 | GPL4133 | 50.19091 | 191.7727 | 3.82087 | 1.9339 |
| DataSet_01_012 | PTGS2 | FLI1 | shRNA | Bone_marrow | WE68 | GSE14543 | GPL96 | 4.99488 | 25.17151 | 5.03946 | 2.33327 |
| DataSet_01_015 | PTGS2 | FLI1 | shRNA | Bone_marrow | STA-ET-1 | GSE14543 | GPL96 | 4.99779 | 38.611 | 7.7256 | 2.94965 |
| DataSet_01_017 | PTGS2 | ERG | siRNA | Umbilical_vein | HUVEC | GSE14801 | GPL570 | 148.075 | 784.075 | 5.29512 | 2.40466 |
| DataSet_01_021 | PTGS2 | STAT1 | shRNA | Upper_aerodigestive_tract | SCC61 | GSE15845 | GPL6244 | 205.0739 | 100.2529 | 0.48886 | -1.0325 |
| DataSet_01_022 | PTGS2 | RUNX1 | siRNA | Haematopoietic_and_lymphoid_tissue | HTB58 | GSE16238 | GPL570 | 114.6914 | 50.18663 | 0.43758 | -1.19238 |
| DataSet_01_029 | PTGS2 | ATM | siRNA | Embryo_kidney | HEK293 | GSE1676 | GPL201 | 39.13333 | 11.21667 | 0.28663 | -1.80275 |
| DataSet_01_030 | PTGS2 | RELA | siRNA | Embryo_kidney | HEK293 | GSE1676 | GPL201 | 39.13333 | 16.91667 | 0.43228 | -1.20995 |
| DataSet_01_031 | PTGS2 | TP53 | siRNA | Embryo_kidney | HEK293 | GSE1676 | GPL201 | 39.13333 | 13.25 | 0.33859 | -1.56241 |
| DataSet_01_039 | PTGS2 | TP63 | shRNA | Mammary_gland | MCF10A | GSE20286 | GPL571 | 28.12888 | 61.46998 | 2.1853 | 1.12783 |
| DataSet_01_050 | PTGS2 | STAT6 | siRNA | Lung | NCI-H460 | GSE25942 | GPL6884 | 99.05372 | 253.1862 | 2.55605 | 1.35392 |
| DataSet_01_061 | PTGS2 | GATA2 | siRNA | Endothelium | HMVEC | GSE28304 | GPL570 | 75.85 | 34.775 | 0.45847 | -1.1251 |
| DataSet_01_070 | PTGS2 | HOXD9 | siRNA | Central_nervous_system | U87 | GSE28618 | GPL570 | 55.11445 | 23.9988 | 0.43544 | -1.19947 |
| DataSet_01_079 | PTGS2 | MITF | siRNA | Skin | A375 | GSE31534 | GPL570 | 10.32733 | 26.24761 | 2.54157 | 1.34572 |
| DataSet_01_103 | PTGS2 | HNF1B | shRNA | Ovary | RMG2 | GSE37290 | GPL570 | 29.10994 | 12.45877 | 0.42799 | -1.22435 |
| DataSet_01_110 | PTGS2 | HOXA1 | siRNA | Lung_fibroblast | IMR90 | GSE37690 | GPL13607 | 0.03506 | 2.85642 | 81.4826 | 6.34842 |
| DataSet_01_113 | PTGS2 | GABPA | siRNA | Lung | A549 | GSE38332 | GPL570 | 690.3432 | 1976.812 | 2.86354 | 1.5178 |
| DataSet_01_122 | PTGS2 | FOXM1 | siRNA | Central_nervous_system | U87 | GSE40051 | GPL570 | 168.8106 | 1623.502 | 9.6173 | 3.26563 |
| DataSet_01_127 | PTGS2 | ZNF254 | siRNA | Gastrointestinal_tissue | GIST-T1 | GSE40080 | GPL5175 | 780.1837 | 267.4212 | 0.34277 | -1.5447 |
| DataSet_01_130 | PTGS2 | ZNF708 | siRNA | Gastrointestinal_tissue | GIST-T1 | GSE40080 | GPL5175 | 780.1837 | 346.651 | 0.44432 | -1.17033 |
| DataSet_01_139 | PTGS2 | POLR3A | siRNA | Mammary_gland | MCF7 | GSE42239 | GPL570 | 28.05 | 13.6 | 0.48485 | -1.04439 |
| DataSet_01_140 | PTGS2 | SOX4 | shRNA | Parotid_gland | ACC3 | GSE4225 | GPL96 | 205.5819 | 551.4843 | 2.68255 | 1.42361 |
| DataSet_01_168 | PTGS2 | STAT3 | siRNA | Peripheral_blood | CD4+ T cells | GSE46333 | GPL14550 | 6.00088 | 2.3962 | 0.39931 | -1.32442 |
| DataSet_01_173 | PTGS2 | ESRRG | siRNA | Placenta_tissue | Trophoblast cells | GSE46463 | GPL10558 | 134.3471 | 275.5751 | 2.05122 | 1.03648 |
| DataSet_01_183 | PTGS2 | TP63 | shRNA | Bladder | UC14 | GSE48124 | GPL10558 | 744.0367 | 136.8679 | 0.18395 | -2.4426 |
| DataSet_01_190 | PTGS2 | GABPA | shRNA | Prostate | C4-2B | GSE49083 | GPL10558 | 7.38836 | 11352.93 | 1536.6 | 10.5855 |
| DataSet_01_191 | PTGS2 | GABPA | shRNA | Prostate | LNCaP | GSE49083 | GPL10558 | 7.28859 | 5426.709 | 744.549 | 9.54022 |
| DataSet_01_198 | PTGS2 | TP63 | siRNA | Head_and_neck | SCC-1 | GSE4975 | GPL96 | 605.4 | 101.6 | 0.16782 | -2.57499 |
| DataSet_01_201 | PTGS2 | TP63 | siRNA | Head_and_neck | SCC-012 | GSE4975 | GPL570 | 148 | 68.8 | 0.46486 | -1.10512 |
| DataSet_01_202 | PTGS2 | TP63 | siRNA | Head_and_neck | SCC-6 | GSE4975 | GPL570 | 1173.1 | 282.55 | 0.24086 | -2.05375 |
| DataSet_01_207 | PTGS2 | MITF | shRNA | Skin | COLO829 | GSE50649 | GPL6244 | 29.82396 | 136.5997 | 4.58022 | 2.19542 |
| DataSet_01_208 | PTGS2 | FOXP1 | siRNA | Haematopoietic_and_lymphoid_tissue | OCI-Ly10 | GSE51382 | GPL570 | 2.89807 | 0.4993 | 0.17229 | -2.53713 |
| DataSet_01_209 | PTGS2 | FOXP1 | siRNA | Haematopoietic_and_lymphoid_tissue | OCI-Ly1 | GSE51382 | GPL570 | 2.80104 | 0.54497 | 0.19456 | -2.36172 |
| DataSet_01_210 | PTGS2 | FOXP1 | siRNA | Haematopoietic_and_lymphoid_tissue | OCI-Ly7 | GSE51382 | GPL570 | 4.62012 | 0.37088 | 0.08028 | -3.6389 |
| DataSet_01_212 | PTGS2 | EGR3 | shRNA | Prostate | M12 | GSE52108 | GPL570 | 294.6279 | 82.83106 | 0.28114 | -1.83066 |
| DataSet_01_219 | PTGS2 | ATM | shRNA | Mammary_gland | MCF10A | GSE54268 | GPL10558 | 1481.014 | 476.4431 | 0.3217 | -1.6362 |
| DataSet_01_224 | PTGS2 | HIF1A | shRNA | Liver | HuH7 | GSE55212 | GPL14951 | 1482.683 | 641.3396 | 0.43255 | -1.20905 |
| DataSet_01_226 | PTGS2 | KLF2 | shRNA | Bone_marrow | RPMI8226 | GSE55667 | GPL570 | 19.33128 | 6.97923 | 0.36103 | -1.4698 |
| DataSet_01_229 | PTGS2 | ESR2 | siRNA | Mammary_gland | BT549 | GSE57379 | GPL11532 | 62.6829 | 127.4096 | 2.03261 | 1.02333 |
| DataSet_01_236 | PTGS2 | FOXC2 | siRNA | Lymph | Lymphatic endothelial cells | GSE60152 | GPL6244 | 671.2005 | 262.3847 | 0.39092 | -1.35507 |
| DataSet_01_237 | PTGS2 | MYC | shRNA | Soft_tissue | HT1080 | GSE60558 | GPL16686 | 14.89318 | 79.20612 | 5.31828 | 2.41096 |
| DataSet_01_253 | PTGS2 | TWIST1 | shRNA | Stomach | CAF32 | GSE62738 | GPL13607 | 259.4225 | 3751.053 | 14.4592 | 3.85392 |
| DataSet_01_254 | PTGS2 | TWIST1 | shRNA | Stomach | CAF | GSE62740 | GPL13607 | 182.6199 | 801.8564 | 4.39085 | 2.1345 |
| DataSet_01_257 | PTGS2 | YBX1 | shRNA | Mammary_gland | MDA | GSE63563 | GPL10558 | 74.55433 | 471.1576 | 6.31965 | 2.65985 |
| DataSet_01_260 | PTGS2 | SOX4 | shRNA | Mammary_gland | MDA-MB231 | GSE63957 | GPL10904 | 18.77225 | 9.23006 | 0.49169 | -1.02419 |
| DataSet_01_281 | PTGS2 | MSX1 | siRNA | Tooth | Dental Pulp Cells | GSE69992 | GPL17077 | 1.51595 | 0.61229 | 0.4039 | -1.30793 |
| DataSet_01_283 | PTGS2 | AHR | siRNA | Haematopoietic_and_lymphoid_tissue | THP-1 | GSE70200 | GPL16686 | 326.7991 | 72.91734 | 0.22313 | -2.16407 |
| DataSet_01_293 | PTGS2 | POSTN | shRNA | Brain | GSC272 | GSE73071 | GPL570 | 22.55753 | 165.7092 | 7.34607 | 2.87697 |
| DataSet_01_295 | PTGS2 | TFAP4 | siRNA | Brain | BE(2)-C | GSE74626 | GPL570 | 5.31539 | 1.38484 | 0.26053 | -1.94046 |
| DataSet_01_304 | PTGS2 | ETV5 | siRNA | Soft_tissue | hADSC | GSE75692 | GPL10558 | 1035.226 | 2391.483 | 2.31012 | 1.20796 |
| DataSet_01_313 | PTGS2 | OVOL2 | siRNA | Corneal_epithelial | Corneal epithelial cells | GSE79251 | GPL14550 | 0.12194 | 0.04122 | 0.33804 | -1.56472 |
| DataSet_01_315 | PTGS2 | ARNT | siRNA | Upper_aerodigestive_tract | HSC3 | GSE80347 | GPL2895 | 103.9145 | 960.9971 | 9.24796 | 3.20914 |
| DataSet_01_318 | PTGS2 | ELK3 | shRNA | Mammary_gland | MDA-MB231 | GSE83325 | GPL15207 | 17.71979 | 7.03056 | 0.39676 | -1.33364 |
| DataSet_01_324 | PTGS2 | MYC | siRNA | Colon | HCT116 | GSE87693 | GPL20844 | 29.85584 | 116.9479 | 3.91709 | 1.96978 |
| DataSet_01_325 | PTGS2 | TP63 | shRNA | Immortalized_keratinocyte | HaCaT | GSE88832 | GPL6244 | 341.4663 | 1273.156 | 3.7285 | 1.89859 |
| DataSet_01_328 | PTGS2 | TP63 | shRNA | Immortalized_keratinocyte | HaCaT | GSE88861 | GPL6244 | 341.4663 | 1273.156 | 3.7285 | 1.89859 |
| DataSet_01_333 | PTGS2 | HOXC6 | siRNA | Prostate | LNCaP | GSE9182 | GPL96 | 5.45 | 11.55 | 2.11927 | 1.08356 |
| DataSet_01_345 | PTGS2 | ARID1A | shRNA | Uterus | iEEC16 | GSE86572 | GPL10558 | - | - | 5.73192 | 2.51902 |
| DataSet_01_360 | PTGS2 | SOX17 | siRNA | Umbilical_vein | HUVEC | GSE37395 | GPL10332 | - | - | 0.38153 | -1.39012 |
| DataSet_01_362 | PTGS2 | GTF2B | siRNA | Cervix | HeLa | GSE48847 | GPL4133 | - | - | 2.12582 | 1.08802 |
| DataSet_02_132 | PTGS2 | AGO2 | shRNA | Haematopoietic_and_lymphoid_tissue | K562 | ENCSR495YSS | - | 0.025 | 0.005 | 0.2 | -2.32193 |

**Original images**


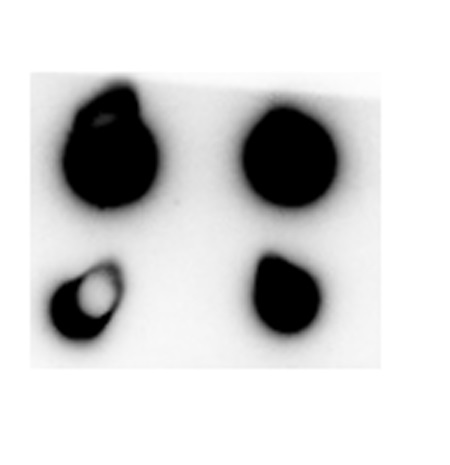


Biotin labeling efficiency assay using an anti-biotin antibody conjugated by HRP with ECL Kit


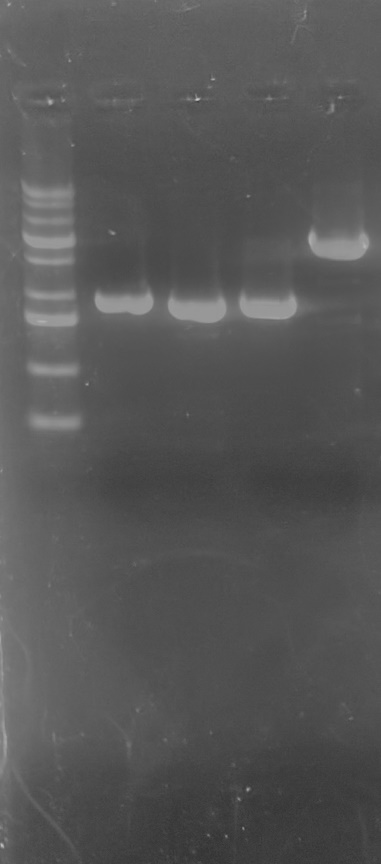


The production of the four biotin-labeled sequences was validated by 1% agarose gel eletrophoresis.
